# Targeted Molecular Construct for Bioorthogonal Theranostics of PD-L1-Expressing Cancer Cells

**DOI:** 10.1021/jacsau.2c00328

**Published:** 2022-07-01

**Authors:** Shiao Y. Chow, Asier Unciti-Broceta

**Affiliations:** Cancer Research UK Edinburgh Centre, Institute of Genetics and Cancer, University of Edinburgh, Crewe Road South, Edinburgh EH4 2XR, U.K.

**Keywords:** bioorthogonal, IEDDA cycloaddition, tumor targeting, tumor labeling, targeted chemotherapy

## Abstract

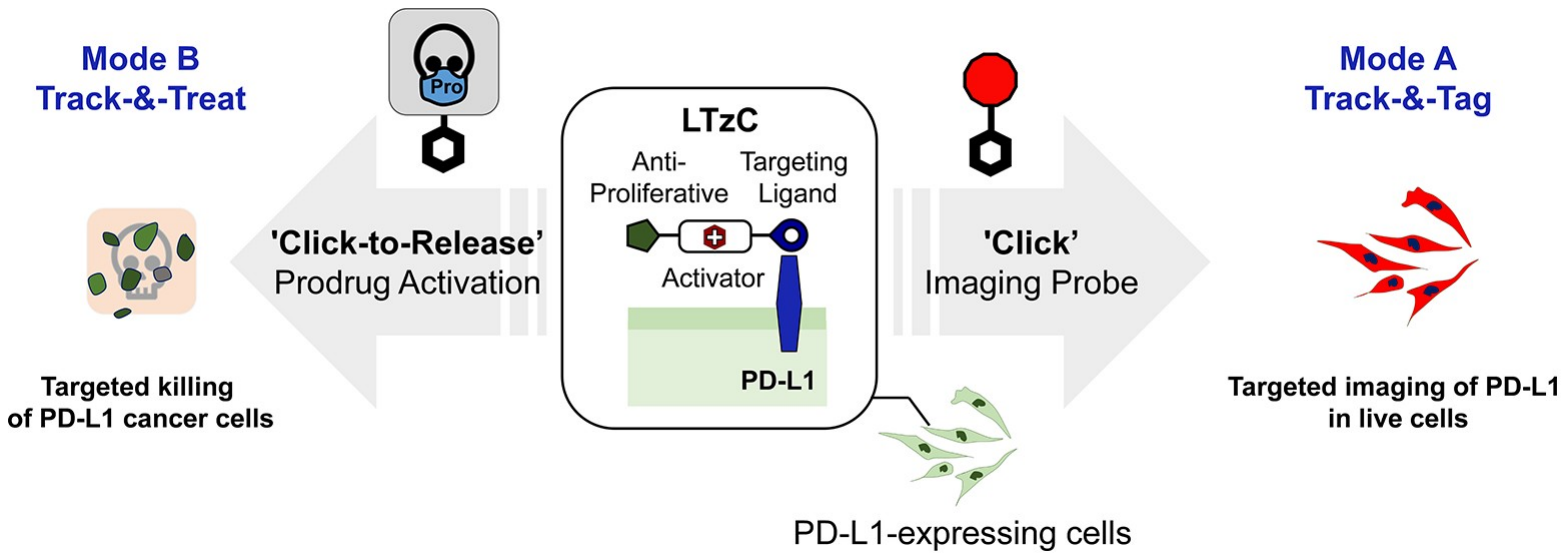

Molecular targeting of tumor-overexpressed oncoproteins can improve the selectivity and tolerability of anticancer therapies. The immunoinhibitory membrane protein programmed death ligand 1 (PD-L1) is highly expressed on certain tumor types, which masks malignant cells from T cell recognition and creates an optimal environment for the cancer to thrive and spread. We report here a ligand-tetrazine conjugate (**LTzC**) armed with a PD-L1 small molecule inhibitor to selectively target PD-L1-expressing cancer cells and inhibit PD-L1 function and conjugated to a tetrazine module and a lipoyl group to incorporate bioorthogonal reactivities and an oxidative stress enhancer into the construct. By pairing **LTzC** with an imaging probe, we have established a “track-&-tag” system for selective labeling of PD-L1 both on and in living cells using click chemistry. We have further shown the specificity and versatility of **LTzC** by click-to-release activation of prodrugs and selective killing of PD-L1-expressing breast cancer cells, offering a new multimodal approach to “track-&-treat” malignant cells that are capable of evading the immune system.

## Introduction

**Figure 1:**
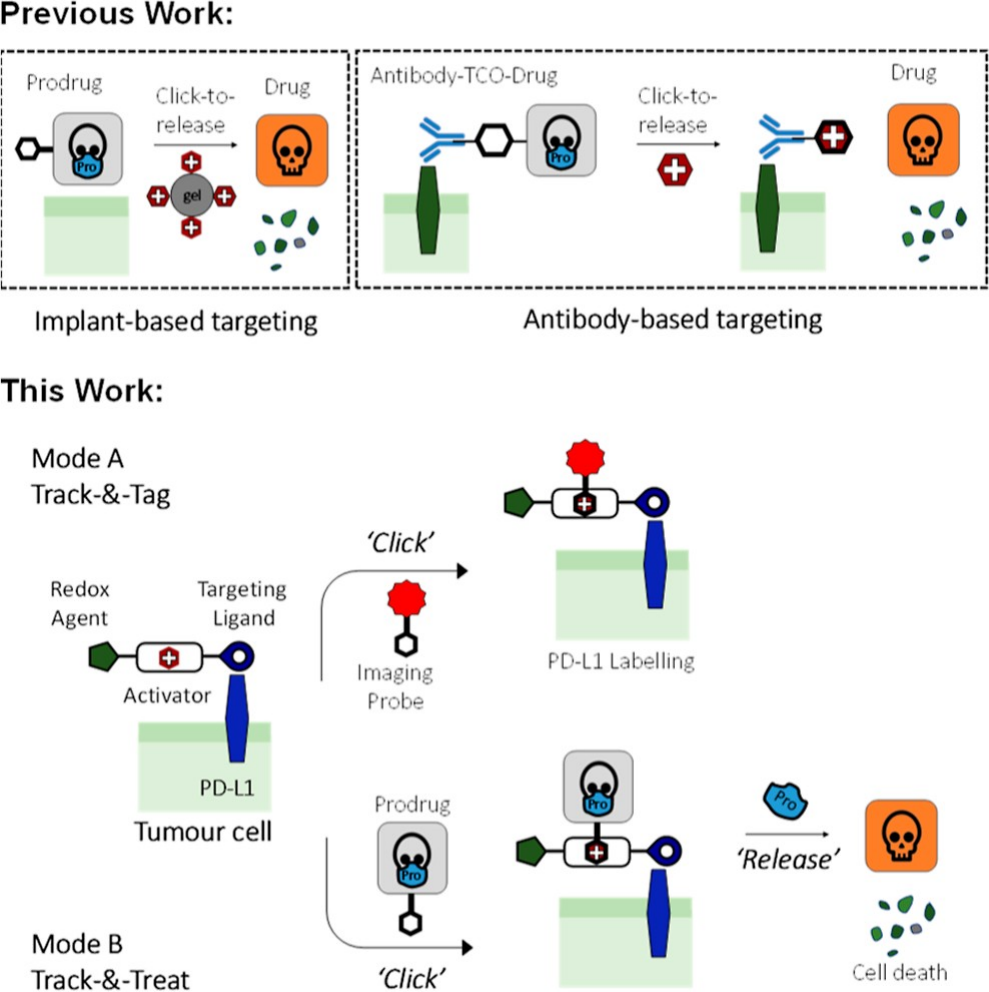
Top: cancer-targeted “click-to-release” prodrug activation strategies reported by Mejia Oneto^6^ (left) and Robillard^4^ (right). Bottom: small molecule-based targeting multimodal strategy reported in this work.

Chemotherapy—as a single agent or in combination—continues to play a pivotal role in the treatment of malignant tumors, especially in advanced stages. Aiming to improve its efficacy and tolerability, recent years have witnessed increased research efforts to develop methods that concentrate the cytotoxic action of chemotherapeutics at the cancer site.^1−3^ Click-to-release strategies are one of those methods.^4−10^ Because of the exquisite chemoselectivity and fast kinetics of the inverse electron demand Diels–Alder (IEDDA) cycloaddition, this reaction not only has been extensively used as a bioconjugation tool in chemical biology, biotechnology, and diagnostics^11−14^ but also facilitates superb control over bioorthogonal dissociative processes. Through the exploitation of bond cleavage reactions triggered by a single IEDDA cycloaddition, Robillard, Chen, Mejia Oneto, and others have shown the efficacy of this strategy to cleave antibody–drug conjugates on demand^15−17^ or uncage masked bioactive molecules in vitro and in vivo (Figure 1, top).^4−10,18−25^

Upregulation of immune checkpoint proteins such as PD-L1 on the surface of cancer cells results in T cell suppression and tumor escape from immune surveillance. Overexpression of PD-L1 is found across many tumor types, including metastatic triple negative breast cancer (TNBC).^26−28^ Targeting immune checkpoints with monoclonal antibodies has revolutionized the treatment of several difficult-to-treat cancers,^28^ whereas small molecule inhibitors of PD-L1 have also shown promising pro-immune antitumor effects in preclinical models.^29^ Nonetheless, despite their success, immunotherapy is in most cases insufficient to eradicate metastatic tumors in patients with advanced cancer, even in combination with chemotherapy. This is partly due to the harm caused by cytotoxic drugs to immune cells, which limits the efficacy of the combined anticancer treatment.

To exploit the benefits and tackle the drawbacks of current immuno–chemotherapy combinations, we envisioned the design of a new strategy to enable cancer cell targeting and treatment in a poly-pharmacological manner by assembling a PD-L1 small molecule inhibitor, BMS-202,^30^ with a “clickable” tetrazine moiety and a natural oxidative stressor.^31^ The BMS-202-derived module of the conjugate would serve both as a tumor-homing motif and an inhibitor of cancer immune evasion, while the integration of a tetrazine group aimed to enable labeling with imaging agents and for click-to-release activation of therapeutics (Figure 1, bottom). Based on the breast cancer antiproliferative activity of lipoic acid (a.k.a. thioctic acid)^31^ and the redox role of lipoylation on metabolic enzymes,^32^ this group was incorporated at the end of the construct to deliver potential additive anticancer effects to the targeted cancer cells.

## Results and Discussion

### Design and Synthesis of **LTzC**

**Figure 2:**
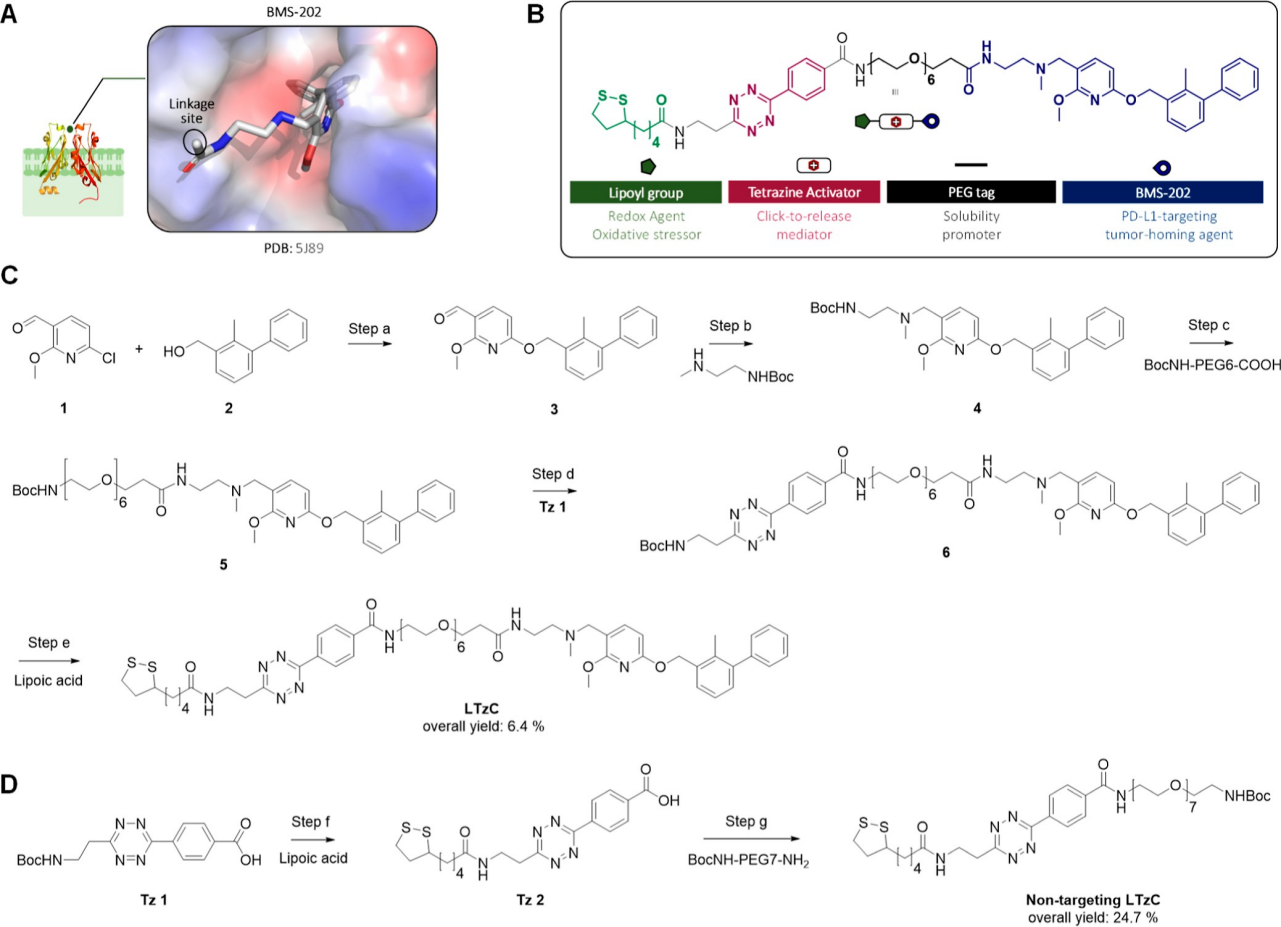
(A,B) Structural and functional design of a **LTzC**. The construct comprises: (a) BMS-202-derived end (blue) to target PD-L1-overexpressing cells; (b) PEG-linker (black), as a solubility tag; (c) a tetrazine (magenta) for IEDDA reactions; and (d) dithiolane ring (green), as a redox agent. BMS-202 attachment strategy was identified using molecular modelling studies (PDB: 5J89). (C) Synthesis of **LTzC**. (a) Pd(OAc)_2_, Xphos, CsCO_3_, toluene, 80 °C, 24 h, 47%. (b) *tert*-Butyl (2-(ethylamino)ethyl)carbamate, sodium triacetoxyborohydride, DCE, room temperature, 24 h, 53%. (c) (i) 1 N HCl in dioxane, 2 h; (ii) Amberlyst A-21, DCM, 30 min; (iii) BocNH-PEG6-COOH, BOP, DIPEA, DMF, r.t., overnight, 88%. (d) (i) 1 N HCl in dioxane, 2 h; (ii) Amberlyst A-21, DCM, 30 min; (iii) **Tz 1**, BOP, DIPEA, r.t., overnight, 62%. (e) (i) 1 N HCl in dioxane, 2 h; (ii) lipoic acid, BOP, DIPEA, DMF, r.t., overnight, **LTzC** = 47%. Overall yield = 6.4%. (D) Synthesis of **Non-targeting LTzC**. (f) (i) TFA/DCM (1:1), 30 min; (ii) lipoic acid, BOP, DIPEA, DMF, r.t., overnight, 59%. (g) Boc(NH)-PEG7-NH_2_, BOP, DIPEA, DMF, r.t., overnight, 42%. Overall yield = 24.7%.

After analyzing the co-crystal structure of BMS-202 bound to PD-L1^30^ (Figure 2A), we identified the acetamide terminal of the ligand as the optimal conjugation site. As the acetamide end is solvent-exposed, we postulated that using this site as a growing vector on BMS-202 would impose minimal impact on the key binding interactions essential for the targeting of PD-L1 (Figure S1). The central design of the proposed heterofunctionalized tetrazine, named **L**igand **T**etra**z**ine **C**onjugate (**LTzC**), in analogy to other multifunctional chimeric molecules, comprised: (i) BMS-202 as a cancer-targeting motif and pro-immune effector, (ii) an internal tetrazine moiety as the bioorthogonal clickable module, and (iii) a terminal lipoyl group to promote redox-mediated antiproliferative effects (Figure 2B). The synthesis of **LTzC** was performed following the strategy described in Figure 2C. First, BMS-202 precursor **3** was synthesized via palladium-catalyzed C–O cross-coupling between aryl chloride **1** and benzyl alcohol **2**. Introduction of the diamine linker was performed through reductive amination of **3** with *tert*-butyl-2-(ethylamino)ethylcarbamate using sodium triacetoxy borohydride to yield intermediate **4**. Standard Boc-deprotection and amide coupling conditions were used to introduce the heterofunctionalized BocNH-PEG6-COOH spacer to yield **5**. A subsequent Boc-deprotection and amide coupling with tetrazine building block 1 (**Tz 1**) yielded intermediate **6**. A final Boc-deprotection followed by amide coupling with lipoic acid yielded the final **LTzC**. A control construct (**non-targeting LTzC**) was prepared via Boc-deprotection of **Tz 1**, amide coupling with lipoic acid, and coupling with BocNH-PEG7-NH_2_ (Figure 2D).

### Bioorthogonal Reactivity of **LTzC**

**Figure 3:**
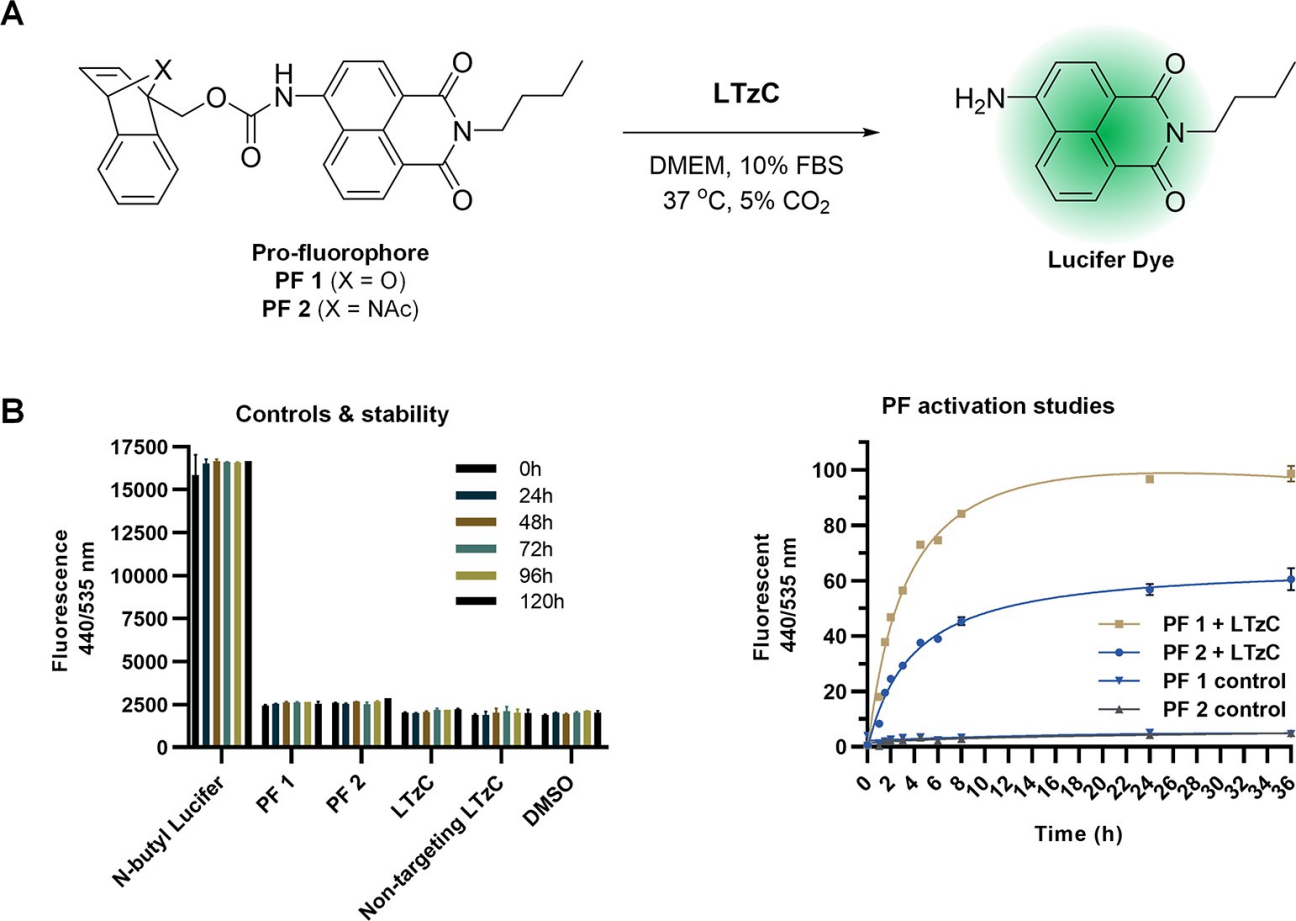
(A) Click-to-release conversion of pro-fluorophores **PF 1** or **PF 2** (10 μM) to free Lucifer dye by reaction with **LTzC** (10 μM) under physiological conditions. Dye release was quantified by fluorescence (λ_ex_ = 440; λ_em_ = 535). (B) Left: control/stability studies. Reagents were incubated separately and fluorescence monitored for 120 h. Right: study of fluorescence generation over time. **PF 1** and **PF 2** were incubated without (control) or with **LTzC** and monitored for 36 h. The data are average of triplicates, and the error bars indicate standard deviations.

We first developed a fluorescent assay for the functional assessment of **LTzC** and its building blocks to perform IEDDA reactions under physiological conditions (Figure 3). As reactive partners, we chose norbornadiene-based chemical groups.^33^ Although *trans*-cyclooctyne (TCO)-based groups are the most widely used reactive partners for tetrazines due to its superior reaction kinetics,^34^ benzonorbornadienes (BNBD) are more synthetically accessible than TCO and display exceptional stability in biological media.^33^ Two novel BNBD-protected pro-fluorophores of the green-emitting dye *N*-butyl-Lucifer,^35^**PF 1** (7-oxa-BNBD-Lucifer) and **PF 2** (7-acetyl-7-aza-BNBD-Lucifer), were synthesized using a modified protocol from a previous report^33^ and used as IEDDA reaction substrates (Figure 3A). Here, the BNBD group functions as a tetrazine-sensitive masking group to quench the dye and facilitate restoration of the fluorophore upon release of the electron-donating amino group. Control experiments demonstrated that the masking strategy successfully quenched the fluorescent properties of **PF 1** and **PF 2**, displaying fluorescent emission equivalent to the DMSO control (baseline level, Figure 3B). Notably, the pro-fluorophores were exceptionally stable under physiological conditions [Dulbecco’s modified Eagle media (DMEM) media supplemented with 10% FBS at 37 °C], with no change in fluorescence activity observed over 5 d, indicating that this masking strategy is optimal for live cell experimentation.

Next, the activation of **PF 1** and **PF 2** by **LTzC** was assessed under the same conditions, and the generated fluorescence emission was monitored using a microplate reader. Rather than using an excess of one of the reactive partners, as it is typically done in most click-to-release studies,^15−25^ the reaction components were introduced in equimolecular quantities. As shown in Figure 3B (right panel), a slower activation rate was observed in the reaction of **PF 2** with the **LTzC**, which suggests that the bulkier acetamide moiety of **PF 2** may impose unfavorable steric hindrance for the initial cycloadditive process with the tetrazine module of the **LTzC**. A similar trend was observed in the activation of **PF 1** and **PF 2** by **non-targeting LTzC** (see Figure S2 in the Supporting Information). Analysis of the conversion studies by HPLC (Figure S3 and S4) confirmed that the initial cycloaddition reaction is the rate-limiting step for **PF 2** activation. Nevertheless, and gratifyingly, quantitative click-to-release activation of **PF 1** was completed within 24 h, with >50% conversion occurring during the first 3 h. Spectrophotometric analysis of **LTzC** disappearance (λ_abs_ = 520 nm) over time by reaction with two BNBD derivatives (7-oxa-BNBD **8** and 7-acetyl-7-aza-BNBD **9**, see Supporting Information) at a range of concentrations was used to generate the kinetics plots and calculate the second order reaction rate constants: 0.055 M^–1^ s^–1^ for 7-oxa-BNBD and 0.017 M^–1^ s^–1^ for 7-acetyl-7-aza-BNBD (Figure S5). The calculated rates of the cycloaddition reaction between **LTzC** and BNBD are consistent with the literature.^33^ Although the reaction is slower than that of Tz/TCO pairs, it is faster than many other bioorthogonal reactive partners.^34^ All these results, which highlight the stability of the pro-fluorophores and the reactivity displayed by **LTzC** toward 7-oxa-BNBN-masked substrates at stoichiometric concentrations and physiological conditions, reinforced our plan to investigate this chemistry for both bioconjugation (protein labeling) and click-to-release (prodrug activation) applications.

### Mode A: “Track-&-Tag” of PD-L1-Expressing Breast Cancer Cells

**Figure 4:**
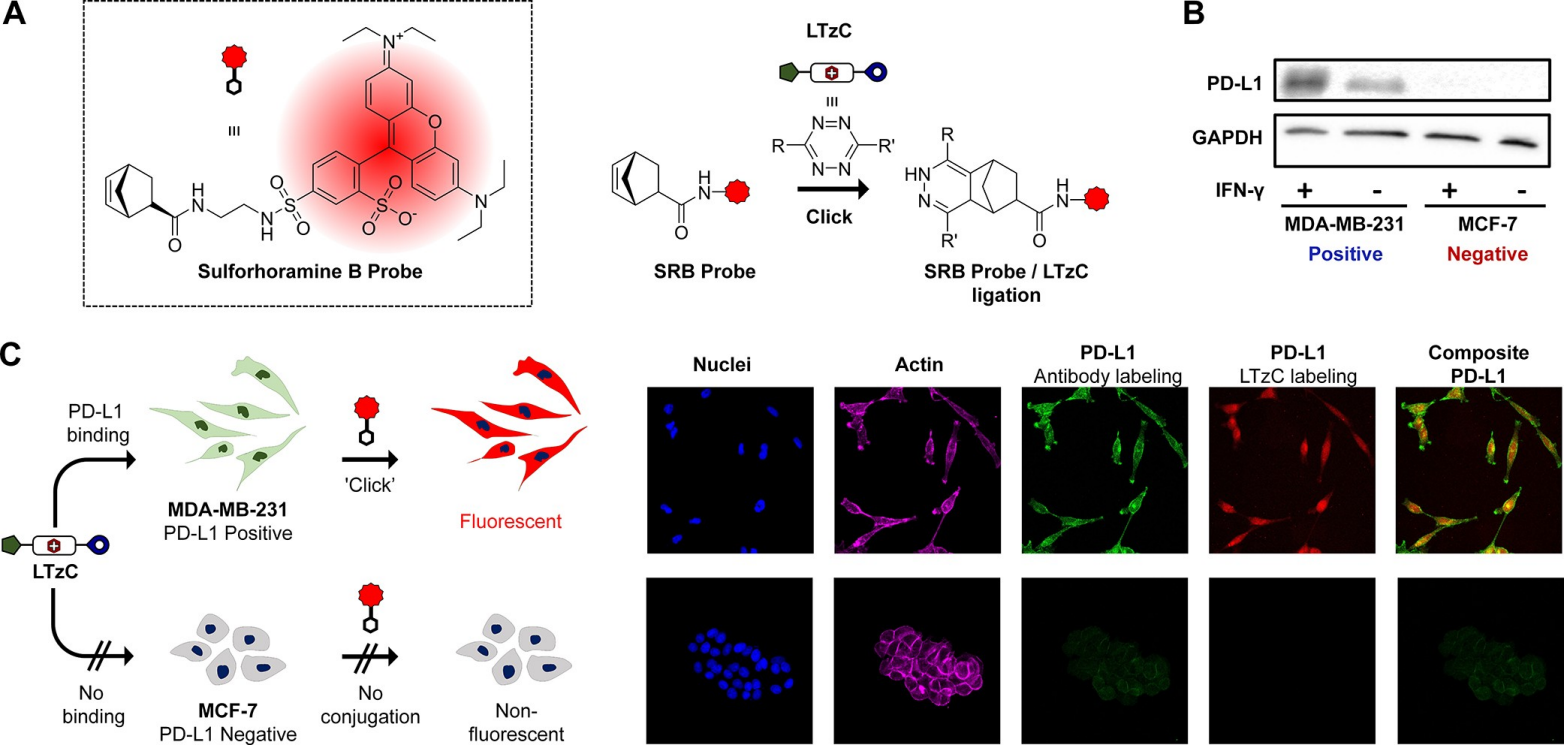
Schematic strategy and confocal imaging of **LTzC**/**SRB probe** bioconjugation method. (A) Structure of the fluorescent *exo*-norbornene-tagged sulforhodamine B probe (**SRB probe**) and ligation strategy with **LTzC**. (B) Western blot analysis of PD-L1 expression in MDA-MB-231 and MCF-7 in the presence and absence of IFN-γ (activator of PD-L1 expression). (C) Targeted bioorthogonal labeling of PD-L1 proteins in PD-L1-positive and -negative cell lines. Cells were first treated with **LTzC** (3 μM) for 4 h to enable targeted binding of PD-L1, followed by removal of unbound **LTzC** and addition of **SRB Probe** (100 nM) for 24 h. Unbound **SRB probe** was removed, and the cells were fixed for staining and imaging experiments. Nuclei were stained by Hoechst 33342; actin was stained using Phalloidin-Alexa Fluor 647. PD-L1 was labeled using antibody-labeling (Human PD-L1 Mab/Alexa Fluor 488 Goat anti-Rabbit IgG) post-fixation to study co-localization of its fluorescent signals with bioorthogonal-labeling (**LTzC**/**SRB Probe**) in MDA-MD-231 cells (merge).

Current detection methods of PD-L1 are typically based on immunohistochemistry and immunofluorescence techniques using biologics-derived modalities.^36,37^ While the specificity of monoclonal antibodies (mAb) for PD-L1 is out of question, the need to use a secondary mAb with low membrane penetrability limits the detection capacity of the method to the outer side of the plasma membrane in live cell assays. Consequently, the presence of cytoplasmic and organelle localized PD-L1 (nuclear translocation of PD-L1 plays an important role in its regulatory functions^38^), including the fraction of PD-L1 that is internalized upon interaction with the PD-L1 mAb, cannot be detected by standard antibody-based techniques in live cells. Encouraged by the biocompatibility and reactivity of **LTzC**, we explored the use of our “track-&-tag” system for the bioorthogonal labeling of PD-L1 in live breast cancer cells. We prepared a norbornene-tagged fluorescent probe based on sulforhodamine B (**SRB probe**) to enable click conjugation to tetrazine-functionalized **LTzC** (Figure 4A). This was designed to enable fluorescent labeling of PD-L1-targeting **LTzC** after ligand–protein binding through an IEDDA reaction to form an irreversible dihydropyridazine species and, by association, enabling the fluorescent mapping of PD-L1 protein. Here, the *exo*-norbornene bioorthogonal tag was selected as the dienophile due to its superior reactivity with tetrazines compared to the *endo* counterpart.^39^

Targeted bioorthogonal labeling of PD-L1 was carried out with the PD-L1-expressing TNBC cell line MDA-MB-231, using the non-PD-L1-expressing ER + breast cancer cell line MCF-7 as the negative control. Enhancement of PD-L1 expression in MDB-MB-231 cell line was achieved via IFN-γ treatment, based on reported in vitro models^40^ and validated using western blot analysis (Figure 4B). As expected, the same treatment in MCF-7 cells did not yield any change in PD-L1 expression. The bioorthogonal labeling of PD-L1 was then carried out by an initial treatment of cancer cells with **LTzC** for 4 h, followed by multiple washings with PBS to remove unbound **LTzC** species. Fresh DMEM media were added and the **SRB Probe** incubated overnight to form the **LTzC-SRB probe** conjugates. Multiple washings with PBS were performed to remove free and unreacted **SRB probe**. Finally, cells were fixed and a secondary PD-L1 detection method using immunofluorescence staining was performed and visualized using confocal fluorescence microscopy (see Figure S6, for the full panel of control studies). In agreement with our expectations, bioorthogonal-labeling of PD-L1 via **LTzC-SRB probe** conjugation was observed only in MDA-MB-231 cells, while no fluorescent emission was observed in MCF-7 negative control (see Figure 4C). Similar PD-L1 expression profiles were observed using the secondary immunofluorescence detection. These studies strongly support that **LTzC** selectively binds to PD-L1. Consistent with the literature,^41^ the images obtained with our “track-&-tag” method shows the presence of PD-L1 inside the cell, suggesting that an important fraction of these surface receptors is internalized upon binding to **LTzC**. This also demonstrates that the IEDDA conjugation can take place inside the cells without the need for permeabilization by cell fixing. Next, live cell studies were performed to image MDA-MB-231 cells without fixation and thereby compare the results of our bioorthogonal labeling strategy with the standard immunostaining method. Strikingly, the images clearly show that, while immunostaining detects PD-L1 only at the cell surface of MDA-MB-231 cells, the deployment of the **LTzC/SRB probe** pair demonstrates that a significant proportion of PD-L1 proteins are internalized by the interaction with the **LTzC** (Figure S7).

**Figure 5:**
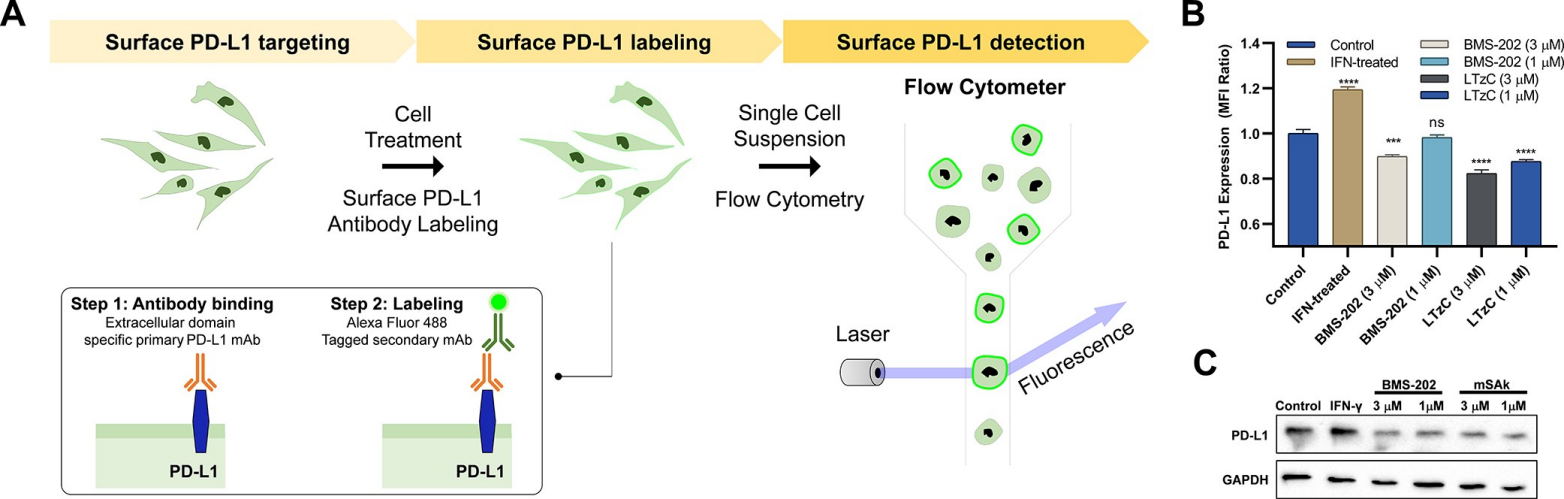
PD-L1 cell surface and total protein expression after IFN-gamma, BMS-202, or **LTzC** treatment in MDB-MB-231 cell line. (A) Workflow of flow cytometry measurement of surface PD-L1 using antibody-labeling. (B) Quantification of surface PD-L1 via flow cytometry. Data are represented as mean fluorescent intensity ratio of control cells vs treated cells (*n* = 3). (C) Western Blot analysis of total PD-L1 expression (*n* = 3). Representative western blots are provided for each data set.

Encouraged by the live cells results, we then investigated the effect of **LTzC** and unmodified BMS-202 on the surface and total expression levels of PD-L1. Flow cytometry quantification of surface PD-L1 after immunostaining and analysis of total PD-L1 expression by western blot were performed after treatment with the compounds at 1 and 3 μM (see Figure 5A,B). High levels of surface PD-L1 were observed in IFN-γ stimulated MDB-MB-231 cells, while a significant decrease of surface PD-L1 was observed in cells treated with BMS-202 (10% reduction) or **LTzC** (18% reduction) at 3 μM concentration. Total protein analysis revealed a similar trend (Figure 5C), indicating that both BMS-202 and **LTzC** promote internalization and subsequent degradation of PD-L1. Importantly, these results strongly suggest that the capacity of cancer cells to inhibit T cell activity by PD-1/PD-L1 interactions will be reduced by direct treatment with **LTzC**, which would consequently decrease immune response escape.

### Mode B: “Track-&-Treat” Metastatic Breast Cancer Cells

**Figure 6:**
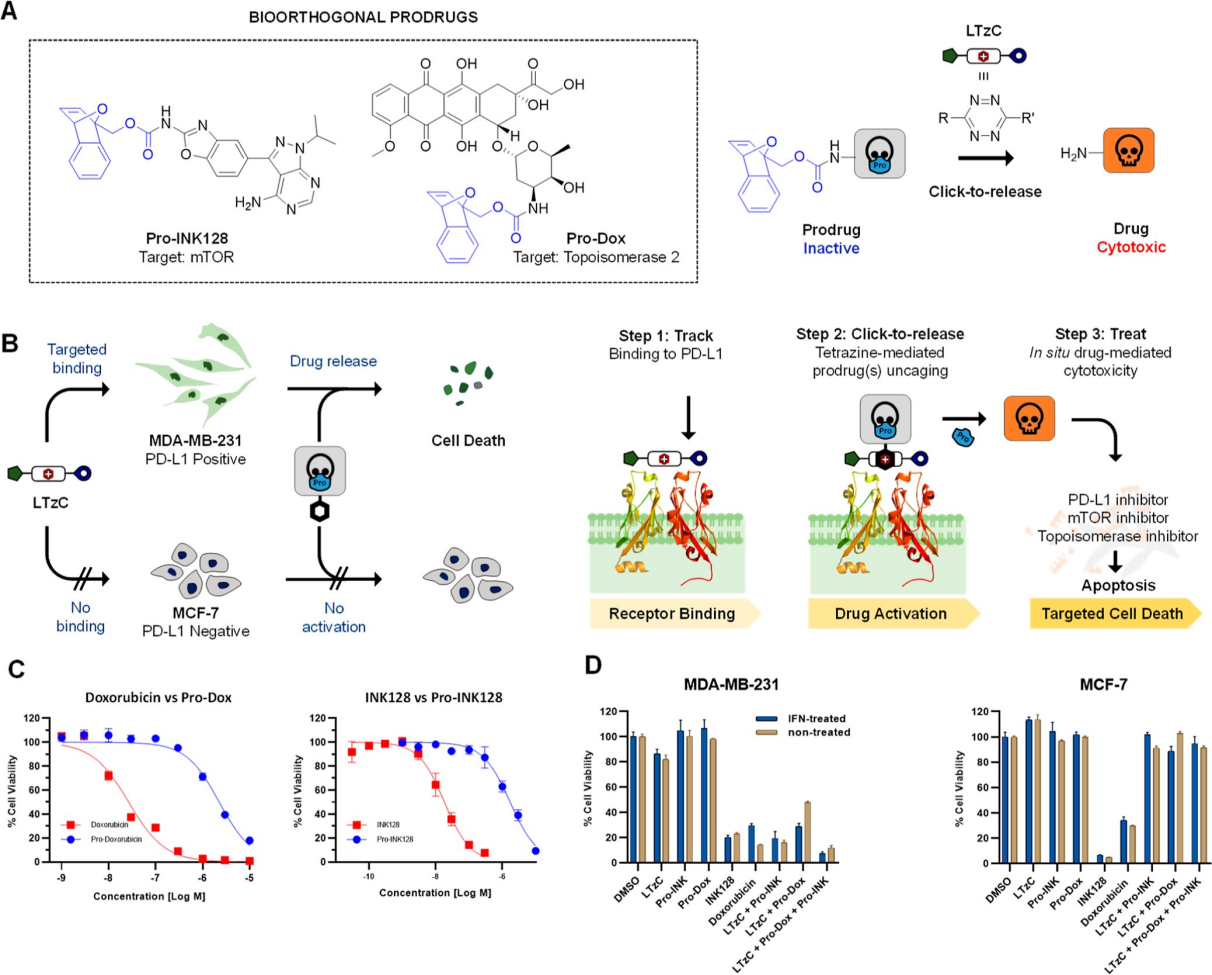
(A) Structure and bioorthogonal activation of 7-oxa-norbornadiene-masked prodrugs **Pro-INK128** and **Pro-Dox**. (B) Track-&-treat strategy: PD-L1 positive cells vs PD-L1 negative cells. (C) Dose response curves of INK128 and **Pro-INK128** (left) and doxorubicin and **Pro-Dox** (right) in MDA-MB-231 cells. (D) Study of the **LTzC**-triggered activation of prodrugs in MDA-MB-231 and MCF-7 cancer cells. Cells were first treated with **LTzC** (3 μM) for 4 h, followed by removal of unbound **LTzC** and addition of **Pro-Dox** (0.3 μM) and/or **Pro-INK128** (0.1 μM). Studies were performed with and without IFN-γ treatment to compare different levels of PD-L1 expression. Cell viability was measured at day 5 using PrestoBlue reagent. Negative controls: **LTzC** (3 μM)/**Pro-Dox** (0.3 μM) or **Pro-INK128** (0.1 μM); positive control: doxorubicin (0.3 μM) or INK128 (0.1 μM). The data are average of triplicates and the error bars indicate standard deviations.

Next, we tested the PD-L1 targeting ability of **LTzC** to perform in situ activation of anticancer agents to “track-&-treat” PD-L1-expressing cancer cells. Based on the superior cargo release obtained with the 7-oxa-BNBD/**LTzC** reaction pair, 7-oxa-BNBD-masked prodrugs of two anticancer agents, an mTOR inhibitor (sapanisertib, a.k.a. INK128) and a cytotoxic drug (doxorubicin), were developed (Figure 6A, see synthesis in the Supporting Information). The choice of INK128 was based on the well-established association of PD-L1 expression and mTOR activity,^42,43^ which indicates that PD-L1-expressing cells are expected to feature over-activation of the mTOR pathway and, therefore, be sensitive to inhibition of mTOR. On the other hand, doxorubicin^44^ is a chemotherapy drug currently approved for the treatment of TNBC. Based on the above, we designed a targeted system for the synergistic co-treatment of PD-L1-expressing TNBC cells by (1) targeting and inhibiting PD-L1 using **LTzC**, (2) inhibiting mTOR activity through activation of **Pro-INK128**, and (3) combining with standard-of-care chemotherapy through uncaging of **Pro-Dox** (Figure 6B). Proof-of-concept studies of this strategy were carried out in PD-L1-expressing MDA-MB-231 cells and non-PD-L1-expressing MCF-7 cells.

Preliminary cell viability studies in both PD-L1 positive and negative cell lines confirmed that the cytotoxicity of **Pro-INK128** (EC_50_ = 2.7 and 8.3 μM in MDA-MB-231 and MCF7, respectively) and **Pro-Dox** (EC_50_ = 1.8 and 2.5 μM in MDA-MB-231 and MCF7, respectively) was successfully masked, compared to their respective parent drugs (see values in Table 1 and dose response curves in Figures 6C and S8).

**Table 1:** Calculated EC_50_ Values^a^ (μM) for Drug and Prodrug Treatments in MDA-MB-231 and MCF-7 Cells

	MDA-MB-231		MCF-7	
compound	non-treated	IFN-γ	non-treated	IFN-γ
**INK128**	0.016	0.042	0.002	0.003
**Pro-INK128**	2.7	1.9	8.3	9.1
**doxorubicin**	0.018	0.028	0.041	0.047
**Pro-Dox**	1.8	1.4	2.5	2.9

a The data are average of triplicates.

We then examined the ability of **LTzC** to elicit bioorthogonal click-to-release activation of **Pro-INK128** and/or **Pro-Dox** in PD-L1-expressing MDA-MB-231. Non-PD-L1-expressing MCF-7 cells were used as a control experiment. Cells were first treated with **LTzC** for 4 h for pre-targeting of PD-L1 receptors on cell surface, followed by multiple washings with PBS to remove unbound **LTzC**. Subsequently, mono- and co-treatment with **Pro-INK128** and **Pro-Dox** were performed to study drug release by PD-L1-bound **LTzC**. The cells were also separately treated with **LTzC**, **Pro-INK128,** or **Pro-Dox** as the negative controls and doxorubicin or INK128 as the positive control. In the mono-prodrug treatments, **Pro-INK128** did not elicit any direct cytotoxic effect on any cell line, whereas highly potent cytotoxicity (85% inhibition) was observed when used in combination with **LTzC** in MDA-MB-231 but not in MCF-7 (Figure 6D). This demonstrates that PD-L1 targeting is required for the in situ tetrazine-mediated click-to-release activation of **Pro-INK128**. Equivalent results were observed for **Pro-Dox**, albeit with reduced efficacy (70% inhibition). In the co-treatment strategy, the combination of **LTzC**, **Pro-INK128**, and **Pro-Dox** led to complete inhibition of cell proliferation. Importantly, the combination of the **non-targeting LTzC** with **Pro-INK128** and/or **Pro-Dox** displayed minimal cytotoxicity effect in both PD-L1 positive and negative cell lines (Figure S9). This further confirms the need of the BMS-202-derived module to effectively target PD-L1-expressing cells. Of note, **LTzC** on its own displayed a minor but evident cytotoxicity effect only in MDA-MB-231 cells, leading to >14% reduction in cell viability at 3 μM but not in MCF-7 cells (Figure 6D). This selective inhibitory effect is attributed to the targeted delivery of the lipoyl module into PD-L1-expressing cells. Additional dose–response cytotoxicity profiling studies revealed that lipoic acid alone indiscriminately elicits around 10% cytotoxic effect in both cell lines and that the lack of the lipoyl module from **LTzC** (**compound 7**) leads to a decrease in cytotoxicity in MDA-MB-231 cells (Figure S8C). This study indicates that lipoylation of the **LTzC** elicits additive anti-cancer effect in the targeted cells.

## Conclusions

In conclusion, we have successfully developed the first cancer-targeting multifunctional construct that can selectively bind PD-L1-expressing cancer cells and trigger IEDDA reactions. The multimodal features of our approach was demonstrated by bioorthogonal fluorescent labeling of PD-L1-expressing cancer cells and by programming PD-L1-selective cell death through click-to-release activation of cytotoxic agents, using the same molecular construct. The combination of **LTzC** and a clickable fluorescent probe enabled the labeling of PD-L1 on and inside live cells, a feature inaccessible using conventional immunostaining. Thereby, we have shown that the PD-L1-targeted **LTzC** triggers protein internalization upon binding, reducing the quantity of surface and total PD-L1. This investigation also represents the first use of PD-L1 small molecule inhibitors as selective cancer cell-targeting ligands to deliver functional cargoes. Last, the versatility of this powerful modular approach facilitates easy introduction of custom-made modifications, thereby opening new opportunities in targeted therapies, sensing, and diagnostics.

## Methods

### Synthetic Procedures

Synthesis of **LTC** and derivatives, **SRB probe**, prodyes **PF 1** and **PF 2,** and prodrugs **Pro-Dox** and **Pro-INK128** was done and characterized as detailed in the Supporting Information.

### **LTzC**-Mediated Activation of **PF 1** and **PF 2**

1 mM stock solutions of **LTzC**, prodyes, and *N*-butyl Lucifer were prepared in biological-grade DMSO. Initial fluorescent profiling control studies were performed to assess fluorescence activities of DMSO, **PF 1** or **PF 2**, or **LTzC**s, and *N*-butyl Lucifer. **PF 1** or **PF 2** and **LTzCs** displayed no interfering fluorescence activity. Deprotection of **PF 1** or **PF 2** by reaction with **LTzC** or non-targeting LTzC was performed in biocompatible conditions (10% FBS in DMEM media, 37 °C) in 96-well flat-bottom plates. Time-course kinetic profiling studies were carried out over 24 h. The final reaction composition was 1 μL of 1 mM **LTzC** (final conc. = 10 μM) and/or 1 μL of 1 mM prodye (final conc. = 10 μM) in 98 μL of DMEM. Conversion of **PF 1** or **PF 2** into fluorescent *N*-butyl Lucifer was monitored via fluorescence detection (λ_ex_ = 440 nm; λ_em_ = 535 nm) using an EnVision Multimode Plate Reader. All measurements were performed in triplicates and analyzed using GraphPad Prism.

### HPLC-MS Analysis of Click-to-Release Reaction

Click-to-release activation of Lucifer dye from **PF 1** or **PF 2** by reaction with **LTzC** was monitored in Agilent 1260 Infinity II Prime LC System using InfinityLab Poroshell 120 EC-C18 column (3 × 100 mm; 2.7 micro). The mobile phase A was 0.1% formic acid in water, and the mobile phase B was acetonitrile. A gradient of 0–100% B ranging from 1 to 7 min was run at a flow rate of 1.0 mL/min. Samples were prepared from stock solutions in DMSO (10 mM of prodye, 3 mM of **LTzC**) to achieve a final concentration of 10 and 12 μM of prodye and **LTzC**, respectively. Samples were incubated at 37 °C, and aliquots were taken at five time points (5, 30 min, 2, 4, 6, and 24 h), diluted by 5-fold with MeCN to quench the reaction, and analyzed by HPLC-MS. The full composition of active species, **LTzC**, **PF 1** or **PF 2**, pyridazine (Pz), and Lucifer dye, was monitored at the 254 nm channel. The conversion of **PF 1** or **PF 2** to Lucifer dye was selectively monitored at the 380 nm channel.

### Cell Culture

Human breast adenocarcinoma MDA-MB-231 and MCF-7 cells were cultured in DMEM supplemented with 10% fetal bovine serum and l-glutamine (2 mM) and maintained in a tissue culture incubator at 37 °C and 5% CO_2_ environment.

### Measurement of PD-L1

MDA-MD-231 and MCF-7 cells were seeded at 24,000 cells per well in 6-well plates. After 24 h, the media were removed and incubated with fresh DMEM media with or without IFN-γ (40 ng/mL). After 24 h incubation, the plate was placed on ice, following by removal of the cell media. The wells were washed thrice with ice-cold PBS, followed by the addition of RIPA lysis buffer (Thermo Scientific) containing protease and phosphatase inhibitors (1.25 mM PMSF, 0.1% v/v aprotinin, 100 μM Na_2_VO_4_, 500 μM NaF). The cells were collected, incubated on ice for 10 min, and centrifuged at 4 °C (17,000×*g*, 10 min). The supernatant was collected, and the total protein content was assessed using Pierce BCA Protein Assay Kit (Thermo Scientific). Appropriate dilution in RIPA buffer was performed to obtain 20 μg cell lysate for each respective samples. The supernatant was then treated with Laemmli Sample Buffer (Bio-Rad) at 95 °C for 5 min, loaded onto Mini-PROTEAN TGX precast gels (Bio-Rad), run in Mini-PROTEAN Tetra Cell tanks in TGS buffer (pH 8.3) at 150 V for 45 min, and transferred onto Trans-Blot Turbo Nitrocellulose membranes (Bio-Rad) using the Trans-Blot Turbo Transfer System (Bio-Rad). The membrane was blocked with a blocking buffer for 1 h at r.t., followed by incubation with the primary antibody (1:1000, Human PD-L1 Mab, R&D Systems Inc.) at 4 °C for 24 h. The membrane was washed twice with TBST, followed by incubation with the secondary antibody (1:1000, Goat Anti-Rabbit IgG HRP, R&D Systems Inc.) for 2 h. The membrane was washed thrice with TBST, followed by addition of Clarity ECL western blotting substrates (Bio-Rad) before imaging in the ChemiDoc XRS + Imaging System (Bio-Rad). Protein bands (PD-L1: ∼50 kDA) were quantified using the ImageLab software v5.2 (Bio-Rad). MDA-MB-231 cells were seeded (24,000 cells per well) in 6-well plates. The same protocol was followed for measuring PD-L1 after treatment with BMS-202 or **LTzC** for 4 h at two concentrations (1 and 3 μM).

### Flow Cytometry Quantification of Surface PD-L1 Population

Surface PD-L1 expression (non-stimulated or IFN-γ stimulated) of MDA-MB-231 cells before and after treatment with BMS-202 or **LTzC** was determined by flow cytometry. MDA-MD-231 cells were seeded at 24,000 cells per well in 6-well plates. After 24 h, the media were removed and incubated with fresh DMEM media with or without IFN-γ (40 ng/mL) as control for 24 h or with drug treatment (BMS-202 or **LTzC**) for 4 h at two concentrations (3 or 1 μM). The cells were washed with PBS (3 times) and treated with trypsin solution in PE buffer for 5 min. 2 mL of DMEM media were added to quench the reaction, and the cells were pipetted multiple times slowly to prepare a single cell suspension. The samples were transferred to centrifuge tubes and centrifuged at 300 G for 5 min. The supernatant was discarded, to which primary anti-PD-L1 antibody (1:500; Extracellular Domain Specific anti-PD-L1 antibody D8T4X Rabbit mAb) in FACs buffer (5% BSA in PBS) was added and incubated on ice for 1 h. The cell suspension was centrifuged at 300 G for 5 min, and the supernatant was removed. The cell pellets were washed three times via centrifugation and supernatant removal. The secondary antibody (1:500, Alexa Fluor 488 Goat anti-Rabbit IgG) was added and incubated on ice for 1 h. Washing steps were carried out, followed by cell fixation (4% paraformaldehyde in PBS, 10 min at room temperature) and resuspension in FACS buffer. Rabbit IgG control was used for each condition as an isotype control. The single cell suspension was analyzed using a BD LSRFortessa X-20 Cell and processed using FlowJo program.

### Track-&-Tag Method

MDA-MB-231 and MCF-7 cells (24,000 cells/well) were seeded in MatTek glass-bottom 6-well plates. After 24 h, the media were removed and incubated with fresh DMEM media with IFN-γ (40 ng/mL). After 24 h, the media were replaced with fresh media in the absence or presence of **LTzC** (3 μM) and incubated for 4 h. The media were removed from the wells, washed with PBS (3 times), and replaced with fresh media containing **SRB probe** (100 nM) for 24 h. Control wells were incubated with DMSO (0.1% v/v). Subsequently, the media were removed and washed with PBS (3 times). Cells were fixed using paraformaldehyde (4% v/v) for 15 min and washed with PBS (3 times). Permeabilization of cells was performed using 0.1% Triton in PBS for 15 min and washed with PBS (3 times). The cells were treated with blocking buffer (PBS, 5% goat serum) for 1 h. The blocking buffer was removed, washed with PBS (3 times), and primary antibody solution (**Ab1**; 1:500, Human PD-L1 mAb in PBS and 1% BSA) was added and incubated at 4 °C for 24 h. AfterQ1: Please check the sentence for completeness. washing with PBS (3 times), secondary antibody solution (**Ab2**, 1:500, Alexa Fluor 488 Goat anti-Rabbit IgG (H + L) in PBS and 1% BSa). The wells were washed with PBS and incubated with Alexa Fluor 647 Phalloidin (1:1000) and Hoerst 33342 (1:10,000) in PBS for 20 min at r.t. The wells were washed with PBS (3 times) and stored in PBS solution at 4 °C. Confocal imaging was performed using a confocal inverted microscope Nikon A1R with a 40× air immersion objective (Plan Fluar 0.75 DIC N2). The image was acquired using NIS-Elements program in a sequential mode using preconfigured settings for Hoechst 33342 (λ_ex_ = 350–410 nm; λ_em_ = 380–440 nm; blue; nuclei), Alexa Fluor 488 (λ_ex_ = 480–505 nm; λ_em_ = 510–540 nm; green; immunostained PD-L1), **SRB probe** (λ_ex_ = 560–600 nm; λ_em_ = 580–620 nm; red; bioorthogonally tagged PD-L1), and Alexa Fluor 647 (λ_ex_ = 630–670 nm; λ_em_ = 650–720 nm; far red; actin).

### Track-&-Treat Method

MDA-MB-231 and MCF-7 cells were seeded in a 96-well plate (1000 cells per well), incubated or 24 h, followed by replacement with fresh media with or without IFN-γ (40 ng/mL). After 24 h, the media were replaced with fresh media containing respective **LTzC**s (3 μM) and incubated for 4 h. The media were removed from the wells, washed with PBS (3 times), and replaced with fresh media containing **Pro-Dox** (0.3 μM) or **Pro-INK128** (0.1 μM) for 5 d. Control wells were incubated with DMSO (0.1% v/v). Experiments were performed in triplicates. PrestoBlue cell viability reagent (10% v/v) was added to each well and the plate was incubated for 90 min. Fluorescence emission was detected using a EnVision Multimode Plate Reader (λ_ex_ = 540 nm; λ_em_ = 590 nm). All conditions were normalized to the untreated cells (100%), and analysis was performed using GraphPad Prism.

## Supporting Information Available

Materials and methods, compound synthesis and characterization, ^1^H and ^13^C NMR spectra, experimental procedures for kinetics studies, and biological assays and confocal imaging studies (PDF)

## Abbreviations

PD-L1: programmed-death receptor ligand 1
IEDDA: inverse-electron demand Diels–Alder
TNBC: triple negative breast cancer
