# Supplementary material for: Targeted Molecular Construct for Bioorthogonal Theranostics of PD-L1-Expressing Cancer Cells
Source: JACS Au. 2022 Jul 1;2(7):1747–56. doi: 10.1021/jacsau.2c00328 (PMC9326819; doi:10.1021/jacsau.2c00328)
Supplement: Supplementary file 1 — au2c00328_si_001.pdf [file au2c00328_si_001.pdf]

Supporting Information

A targeted molecular construct for bioorthogonal theranostics of PD-L1-expressing cancer cells

Shiao Y. Chow and Asier Unciti-Broceta*

Cancer Research UK Edinburgh Centre, Institute of Genetics and Cancer, University of Edinburgh, Crewe Road South, Edinburgh EH4 2XR, UK

*Corresponding author. E-mail: asier.ub@ed.ac.uk

Table of Contents

1. General 2

2. Design, synthesis, and assembly of Ligand-Tetrazine Conjugate (LTzC) 2

2.1 Molecular modelling studies 2

2.2 Synthesis of LTzC and nom-targeting LTzC 2

2.2.1 Synthesis of LTzC 3

2.2.2 Synthesis of non-targeting LTzC 5

2.3 Synthesis of profluorophores, fluorescent probe and prodrugs 6

2.3.1 Synthesis of norbornene precursors 6

2.3.2 Synthesis of profluorophores 1-2 6

2.3.3 Synthesis of SRB Probe 8

2.3.4 Synthesis of prodrugs 8

3. LTzC-mediated deprotection of tetrazine-sensitive probes 9

3.1 Profiling of LTzC-mediated deprotection of tetrazine-sensitive fluorescent probes 9

3.2 HPLC-MS analysis of LTzC-mediated deprotection of tetrazine-sensitive probes 10

3.3 Spectrophotometric analysis and calculation of cycloaddition reaction kinetics 13

4. Biological studies 13

4.1 Cell culture 13

4.2 PD-L1 expression validation studies for cell lines selection 13

4.3 Effect of BMS202 and LTzC on PD-L1 expression 14

5. PD-L1 imaging studies in breast cancer cells 15

5.1 LTzC-mediated labeling of PD-L1 positive cells using mode ‘Track-&-Tag’ 15

5.2 Comparison of LTzC- and antibody-based PD-L1-labeling in live or fixed cells 17

6. Biocompatibility and cell viability studies 19

7. LTzC-mediated pro-drug activation in breast cancer cells 20

8. NMR spectra 21

1. General

Chemicals and solvents were purchased from Sigma-Aldrich, Fisher Scientific, Fluorochem Ltd. or VWR International Ltd. Drug (Doxorubicin) were purchased from Fluorochem Ltd. Recombinant Human IFN-γ was purchased from BioLegend. Primary antibody (Human PD-L1 Mab; Clone 2340D) and secondary antibodies (Goat Anti-Rabbit IgG HRP and Alexa Fluor 488 Goat anti-Rabbit IgG) for Western Blot and immunofluorescent studies were purchased from R&D Systems Inc and Invitrogen. NMR spectra were recorded at ambient temperature on a 500 MHz Avance III spectrometer. Chemical shifts are reported in parts per million (ppm) relative to the solvent peaks. High Resolution MS of novel compounds were performed on a Bruker microTOF focus II mass spectrometer. All compounds used in the biological experiments were > 95% pure, as determined by NMR analysis. Stock solutions were prepared in biological grade DMSO.

2. Design, synthesis, and assembly of Ligand-Tetrazine Conjugate (LTzC)

2.1 Molecular modelling studies

Co-crystallized structure of BMS202 and PD-L1 (PDB: 5j89) was obtained from RCSB Protein Data Bank[1]. Protein-ligand interactions were visualized using the PyMOL Molecular Graphics System (Version 1.2r3pre, Schrödinger, LLC). The terminal acetamide group of PD-L1 bound BMS-202 was observed to be solvent-exposed (Supp. Fig. 1) and not engaged in binding to PD-L1 protein, and therefore identified as the suitable attachment site for the synthetic construction of LTzCs.

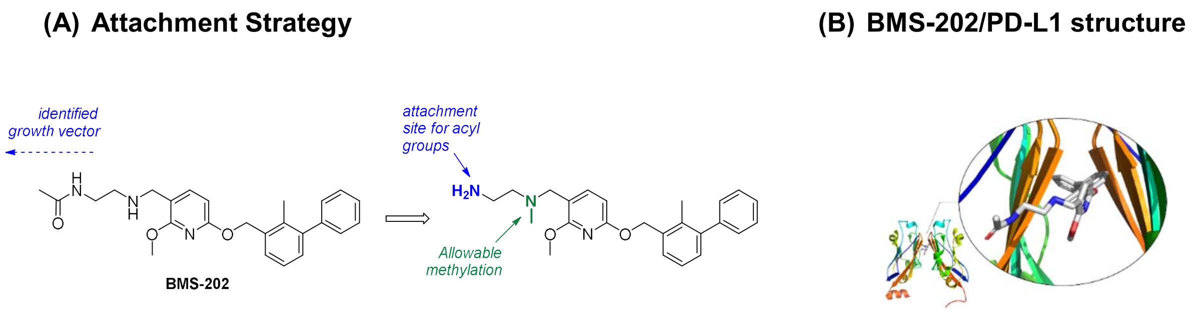

Figure S1. Identification of attachment site on BMS-202 for LTzCs assembly.

2.2 Synthesis of LTzC and nom-targeting LTzC

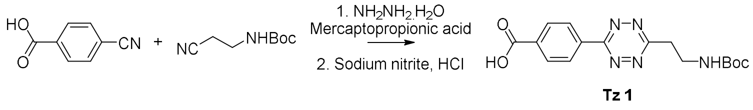

Synthesis of Tetrazine 1[2]. A solution of 4-cyanobenzoic acid (50.0 mg, 0.34 mmol), N-Boc-2-cyano-ethylamine (231 mg, 1.36 mmol) and 3-mercaptopropionic acid (24 mg, 0.068 mmol) in ethanol:DMSO (100:20 uL) was cooled on ice. To this mixture was added dropwise hydrazine hydrate (272 mg, 5.44 mmol). The reaction mixture was allowed to return to room temperature and stirred overnight. The reaction mixture was diluted with ice water, to which sodium nitrite (235 mg, 3.40 mmol) was added. The reaction was kept cold on ice and adjusted to pH 2-3 using 1 M HCl. The crude solution was extracted with EtOAc (50 mL X 3). The

[1] 10.2210/pdb5J89/pdb
[2] W. Mao, W. Shi, J. Li, D. Su, X. Wang, L. Zhang, L. Pan, X. Wu, H. Wu, *Angew. Chem. Int. Ed.* **2019**, 58, 1106

organic layers were collected, washed with brine, dried with $MgSO_4$, filtered and removed in vacuo to afford the crude product. Purification was performed (0-7% MeOH in DCM) using column chromatography to afford **Tz 1** as a pink solid (46 mg, 39% yield).^{1}H NMR (500 MHz, CD_3OD): δ=8.63 (d, J = 8.4 Hz, 2H), 8.23 (d, J = 8.4 Hz, 2H), 3.64 (m, 2H), 3.50 (m, 2H), 1.32 (s, 9H). ^{13}C NMR (126 MHz, CD_3OD): δ= 169.8, 169.0, 165.1, 158.4, 137.5, 135.6, 131.5, 128.8, 80.2, 40.0, 36.9, 28.7. ESI-MS: Calcd. For m/z, $C_{16}H_{19}N_5NaO_4$+ [M+Na]+ 368.13; Found, 368.30

2.2.1 Synthesis of LTzC

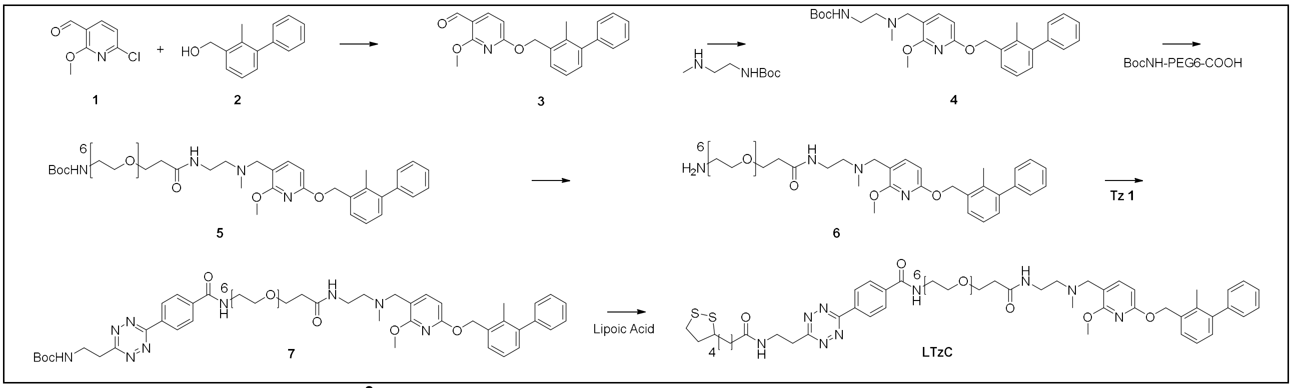

Synthesis of Compound 3[3]**.** 6-chloro-2-methoxypyridine-3-carbaldehyde, **1** (345 mg, 2.0 mmol), (2-methyl[1,1'-biphenyl]-3-yl)methanol, **2** (500 mg. 2.5 mmol), $Pd(OAc)_2$ (57 mg, 0.25 mmol), cesium carbonate (1.64 g, 5.1 mmol), and XPhos (240 mg, 0.50 mmol) were dissolved in anhydrous toluene (20 mL) in a sealed reaction vial and purged by a stream of argon for 5 min. The reaction was heated at 80 °C overnight. The mixture was cooled to room temperature, filtered through a short plug of Celite, and the filtrate was concentrated under reduced pressure. Purification was performed (0-20% EtOAc in Hexane) using column chromatography to afford compound **3** as a white solid (394 mg, 47% yield). ^{1}H NMR (500 MHz, DMSO-D6): δ=10.10 (s, 1H), 8.04 (d, J = 8.3 Hz, 1H), 7.53 – 7.41 (m, 3H), 7.41 – 7.35 (m, 1H), 7.34 – 7.26 (m, 3H), 7.21 (dd, J = 7.7, 1.4 Hz, 1H), 6.60 (dd, J = 8.3, 0.8 Hz, 1H), 5.56 (s, 2H), 4.05 (s, 3H), 2.22 (s, 3H). ^{13}C NMR (126 MHz, DMSO-D6): δ=186.7, 165.9, 164.3, 142.3, 141.3, 140.6, 135.0, 134.1, 129.8, 129.1, 128.5, 128.2, 126.9, 125.5, 111.8, 103.5, 67.3, 54.0, 15.9. ESI-MS: Calcd. For m/z, $C_{21}H_{20}NO_3$+ [M+H]+ 334.14; Found, 334.10

Synthesis of Compound 4. Compound **3** (350 mg,1.1 mmol), tert-butyl-2-(methylamino)ethylcarbamate (366 mg, 2.1 mmol), and sodium triacetoxyborohydride (668 mg, 3.15 mmol) were dissolved in anhydrous DCE (3 mL) and stirred at room temperature under nitrogen atmosphere overnight. The reaction was quenched with $NaHCO_3$ and extracted with EtOAc. The organic layers were collected, dried with $MgSO_4$, filtered and removed in vacuo to afford a sticky oil as the crude product. Purification was performed (0-50% EtOAc in hexane) using column chromatography to afford compound **4** as a clear oil (276 mg, 53% yield). ^{1}H NMR (500 MHz, CD_3OD): δ=7.58 (d, J = 8.0 Hz, 1H), 7.41 (t, J = 7.3 Hz, 3H), 7.37 – 7.30 (m, 1H), 7.27 (dd, J = 8.0, 1.4 Hz, 2H), 7.22 (t, J = 7.6 Hz, 1H), 7.16 (dd, J = 7.6, 1.5 Hz, 1H), 6.40 (d, J = 8.0 Hz, 1H), 5.45 (s, 2H), 3.97 (s, 3H), 3.58 (m, 2H), 3.24 (m, 2H), 2.60 (m, 2H), 2.30 (br s, 3H), 2.25 (s, 3H), 1.43 (s, 9H). ^{13}C NMR (126 MHz, CD_3OD): δ= 165.0, 162.7, 158.9, 145.2, 144.3, 143.5, 137.1, 135.4, 130.9, 130.4, 129.4, 129.2, 128.0, 126.5, 103.3, 81.0, 68.2, 57.4, 55.9, 54.5, 41.4, 37.4, 28.7, 16.4. ESI-HRMS: Calcd. For m/z, $C_{29}H_{38}N_3O_4$+ [M+H]+ 492.2857; Found, 492.2859

[3] S. Basu et. al. *J. Med. Chem.* **2019**, 62, 15, 7250–7263

Synthesis of Compound 5. Compound **4** (270 mg, 0.55 mmol) was treated with 0.5 N HCl in ether (2 mL) and was stirred at room temperature for 4 h. The solvent was removed in vacuo to afford the deprotected product as a crude oil. The crude oil was redissolved in DCM (2 mL) to which Amberlyst A-21 (10 mg) was added and stirred for 30 min. The reaction mixture was filtered and the filtrate was collected and removed in vacuo. The **free-amino intermediate** was obtained as a clear oil (207 mg, 96% yield) and used directly in the next step without further purification. The **free-amino intermediate** (160 mg, 0.41 mmol), BOP (180 mg, 0.41 mmol), BocHN-PEG($_6$)-COOH (186 mg, 0.41 mmol) and DIPEA (211 mg, 1.63 mmol) were dissolved in anhydrous DMF and stirred at room temperature overnight. The reaction mixture was diluted with EtOAc, washed with saturated $NaHCO_3$, then dried over $MgSO_4$. The reaction mixture was filtered and the filtrate was collected and removed in vacuo. Purification was performed (0-8% MeOH in DCM) using column chromatography to afford compound **6** as a yellow oil (299 mg, 88% yield). *HMPA by-product was not completely removed and the product was used directly in the next step. ^{1}H NMR (500 MHz, CD_3OD): δ= 7.62 (d, J = 7.9 Hz, 1H), 7.48 – 7.40 (m, 3H), 7.40 – 7.33 (m, 1H), 7.32 – 7.28 (m, 2H), 7.24 (t, J = 7.6 Hz, 1H), 7.18 (dd, J = 7.4, 1.5 Hz, 1H), 6.43 (d, J = 7.9 Hz, 1H), 5.48 (s, 2H), 3.98 (s, 3H), 3.73-3.35 (m, 29H), 3.26-3.15 (m, 3H), 2.45 (t, J = 6.1 Hz, 2H), 2.33 (br s, 3H), 2.27 (s, 3H), 1.45 (s, 9H). ^{13}C NMR (126 MHz, CD_3OD): δ= 162.7, 158.4, 154.2, 146.1, 145.3, 144.3, 143.5, 137.1, 135.4, 130.9, 130.4, 129.4, 129.2, 128.0, 126.5, 120.5, 103.3, 80.1, 71.6, 71.5, 71.5, 71.5, 71.4, 71.3, 71.3, 68.2, 68.0, 49.6, 49.5, 49.5, 49.5, 49.3, 49.3, 49.2, 49.1, 49.0, 49.0, 48.9, 48.8, 48.8, 48.8, 48.7, 48.7, 48.7, 48.5, 37.4, 37.0, 37.0, 28.8, 28.6, 16.5. ESI-HRMS: Calcd. For m/z, $C_{44}H_{67}N_4O_{11}$+ [M+H]+ 827.4801; Found, 827.4800

Synthesis of Compound 6. Compound **5** (280 mg, 0.34 mmol) was treated with 0.5 N HCl in ether (2 mL) and was stirred at room temperature for 4 h. The solvent was removed in vacuo to afford the deprotected product as a crude oil. The crude oil was dissolved in DCM (2 mL) to which Amberlyst A-21 (20 mg) was added and stirred for 30 min. The reaction mixture was filtered and the filtrate was collected and removed in vacuo. The free-amine intermediate **6** was obtained as a clear oil and used directly in the next step without further purification (241 mg, 98%). ESI-MS: Calcd. For m/z, $C_{39}H_{59}N_4O_9$+ [M+H]+ 727.43; Found, 727.31

Synthesis of Compound 7. Compound **6** (30 mg, 0.041 mmol), **Tz 1 (**14 mg, 0.041 mmol), BOP (18 mg, 0.041 mmol), and DIPEA (11 mg, 0.082 mmol) were dissolved in anhydrous DMF and stirred at room temperature overnight. The reaction mixture was diluted with EtOAc, washed with saturated $NaHCO_3$, then dried over $MgSO_4$. The reaction mixture was filtered and the filtrate was collected and removed in vacuo. Purification was performed (0-10% MeOH in DCM) using column chromatography to afford compound **8** as a pink oil (27 mg, 62% yield). ^{1}H NMR (500 MHz, CD_3OD): δ= 8.63 (d, J = 8.5 Hz, 2H), 8.06 (d, J = 8.5 Hz, 2H), 7.67 (d, J = 8.1 Hz, 1H), 7.45 – 7.36 (m, 3H), 7.35 (m, 1H), 7.27 (dt, J = 8.1, 1.4 Hz, 2H), 7.22 (m, 1H), 7.17 (m, 1H), 6.46 (d, J = 8.1 Hz, 1H), 5.47 (s, 2H), 4.01 (m, 5H), 3.72 – 3.46 (m, 32H), 3.08 (m, 2H), 2.69 (br s, 3H), 2.44 (t, J = 5.9 Hz, 2H), 2.24 (s, 3H), 1.31 (s, 9H). ^{13}C NMR (126 MHz, CD_3OD): δ=174.2, 169.8, 169.3, 165.0, 163.4, 162.3, 158.4, 144.2, 143.5, 139.2, 137.4, 136.3, 135.3, 131.4, 130.8, 130.4, 129.3, 129.3, 129.2, 128.9, 127.9, 126.4, 102.4, 80.1, 71.6, 71.6, 71.5, 71.5, 71.4, 71.4, 71.3, 70.5, 68.2, 67.9, 56.9, 55.5, 54.0, 42.3, 41.2, 40.0, 37.8, 37.7, 36.9, 28.7, 16.4. ESI-HRMS: Calcd. For m/z, $C_{55}H_{76}N_9O_{12}$+ [M+H]+ 1054.5608; Found, 1054.5600

Synthesis of LTzC. Compound **7** (20 mg, 0.019 mmol) was treated with 0.5 N HCl in ether (1 mL) and was stirred at room temperature for 4 h. The solvent was removed in vacuo to afford the deprotected product as a pink oil. The amine.HCl intermediate was unstable in desalting protocol and used directly in the next step without further purification (16 mg, 88% yield). The amine.HCl intermediate (16 mg, 0.017 mmol), lipoic acid (3.5 mg, 0.017 mmol), BOP (7.4 mg, 0.017 mmol), and DIPEA (4.4 mg, 0.033 mmol) dissolved in anhydrous DMF and stirred at room temperature overnight. The reaction mixture was diluted with EtOAc, washed with saturated $NaHCO_3$, then dried over $MgSO_4$. The reaction mixture was filtered and the filtrate was collected and removed in vacuo. Purification was performed (0-10% MeOH in DCM) using column chromatography to afford **LTzC** as a pink oil (9 mg, 47% yield). ^{1}H NMR (500 MHz, CD_3OD): δ= 8.65 (d, J = 8.5 Hz, 2H), 8.07 (d, J = 8.5 Hz, 2H), 7.59 (d, J = 8.0 Hz, 1H), 7.48 – 6.91 (m, 8H), 6.39 (d, J = 8.0 Hz, 1H), 5.44 (s, 2H), 3.95 (s, 3H), 3.79 (t, J = 6.3 Hz, 2H), 3.71 – 3.50 (m, 32H), 3.38 (t, J = 6.3 Hz, 2H), 3.22 – 2.97 (m, 2H), 2.62 (s, 1H), 2.41 (m, 3H), 2.32 (br s, 3H), 2.24 (s, 3H), 2.10 (t, J = 7.3 Hz, 2H), 1.84 (m, 1H), 1.74 – 1.44 (m, 4H), 1.47 – 1.31 (m, 2H). ^{13}C NMR (126 MHz, CD_3OD): δ= 174.9, 172.5, 168.3, 167.9, 163.7, 163.3, 161.8, 160.8, 142.8, 142.7, 142.1, 137.8, 136.1, 134.9, 133.9, 129.3, 129.0, 127.9, 127.9, 127.8, 127.6, 126.5, 125.0, 100.8, 70.2, 70.2, 70.1, 70.1, 70.0, 70.0, 69.9, 69.1, 66.8, 66.4, 56.1, 55.4, 54.0, 52.6, 41.0, 39.8, 39.8, 37.9, 37.4, 36.6, 36.3, 35.3, 34.7, 34.3, 28.3, 25.2, 15.0. ESI-HRMS: Calcd. For m/z, $C_{58}H_{80}N_9O_{11}S_2^+$ [M+H]+ 1142.5413; Found, 1142.5419

2.2.2 Synthesis of non-targeting LTzC

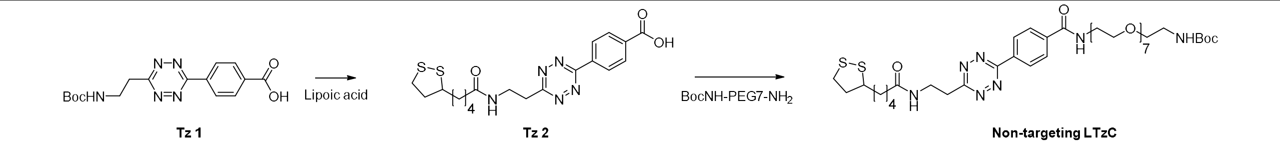

Synthesis of Tetrazine 2. Tz 1 (10 mg, 0.028 mmol) was treated with 10% TFA in DCM (2 mL) at room temperature for 1 h. The solvent was removed in vacuo to afford a pink oil (7 mg). The free amine intermediate was dissolved in anhydrous DMF (1 mL), to which BOP (14 mg, 0.031 mmol) and lipoic acid (6.4 mg, 0.031 mmol) and DIPEA (7.4 mg, 0.057 mmol) were added. The reaction mixture was stirred at room temperature overnight. The reaction mixture was diluted with EtOAc, washed with saturated $NaHCO_3$ and then dried over $MgSO_4$. The reaction mixture was filtered and the filtrate was collected and removed in vacuo. Purification was performed (0-25% MeOH in DCM) using column chromatography to afford **Tz 2** as a pink solid (7 mg, 59% yield). ^{1}H NMR (500 MHz, DMSO-D6): δ=8.57 (d, J = 8.0 Hz, 2H), 8.19 (d, J = 8.0 Hz, 2H), 7.96 (t, J = 5.9 Hz, 1H, N<u>H</u>), 3.63 (q, J = 6.3 Hz, 2H), 3.57 (m, 1H), 3.43 (t, J = 6.5 Hz, 2H), 3.24 – 2.94 (m, 2H), 2.44 – 2.30 (m, 1H), 1.98 (t, J = 7.3 Hz, 2H), 1.84 (m, 1H), 1.60 (m, 1H), 1.57 – 1.46 (m, 1H), 1.46 – 1.37 (m, 2H), 1.28 (m, 2H). ^{13}C NMR (126 MHz, DMSO-D6): δ=172.7, 168.5, 163.6, 130.6, 127.8, 56.6, 38.5, 37.7, 35.5, 35.4, 34.6, 28.6, 25.4. (some quaternary carbon peaks not observed even when slow relaxation time employed) ESI-HRMS: Calcd. For m/z, $C_{19}H_{24}N_5O_3S_2^+$ [M+H]+ 434.1315; Found, 434.1315

Synthesis of non-targeting LTzC. Tz 2 (10 mg, 0.019 mmol), NH(Boc)-PEG7-NH_2 (9.8 mg, 0.020 mmol), BOP (9.2 mg, 0.020 mmol), and DIPEA (4.9 mg, 0.038 mmol) dissolved in anhydrous DMF and stirred at room temperature overnight. The reaction mixture was diluted with EtOAc, washed with saturated $NaHCO_3$, then dried over $MgSO_4$. The reaction mixture was filtered and the filtrate was collected and removed in vacuo. Purification was performed (0-10% MeOH in DCM) using column chromatography to afford **non-targeting LTzC** as a pink oil (7 mg, 42% yield). ^{1}H NMR (500 MHz, CD_3OD): δ= 8.69 (d, J = 8.6 Hz, 2H), 8.11 (d, J = 8.6

Hz, 2H), 4.58 (s, 1H), 3.98 – 3.46 (m, 40H), 3.24 – 2.85 (m, 4H), 2.65 – 2.34 (m, 1H), 2.23 zz– 2.05 (m, 2H), 1.88 (m, 1Hi), 1.72 – 1.29 (m, 13H). ^{13}C NMR (126 MHz, CD_3OD): δ= 174.9, 168.4, 167.9, 163.7, 137.9, 134.9, 127.9, 127.6, 70.2 (multiple overlapping peaks), 70.0, 69.9, 69.7, 69.1, 56.1, 39.9, 39.9, 39.8, 37.9, 37.4, 35.3, 34.7, 34.3, 28.3, 27.4, 25.2. ESI-HRMS: Calcd. For m/z, $C_{40}H_{66}N_7O_{11}S_2$+ [M+H]+ 884.4256; Found, 884.4255

2.3 Synthesis of profluorophores, fluorescent probe and prodrugs

2.3.1 Synthesis of norbornene precursors

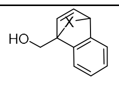

Compound 8 (X=O)
Compound 9 (X=NAc)

Synthesis of compound 8. Compound 8 was prepared according to reported protocol[4]. ^{1}H NMR (500 MHz, $CDCl_3$): δ=7.26 – 7.23 (m, 1H), 7.21 – 7.17 (m, 1H), 7.08 (m, 1H), 7.03 – 6.95 (m, 2H), 6.89 (d, J = 5.5 Hz, 1H), 5.72 (d, J = 1.9 Hz, 1H), 4.61 – 4.33 (m, 2H). ^{13}C NMR (126 MHz, $CDCl_3$): δ=150.8, 147.9, 145.1, 142.5, 125.3, 125.3, 120.3, 119.7, 93.7, 82.4, 60.7. ESI-MS: Calcd. For m/z, $C_{11}H_{10}O_2Na$+ [M+Na]+ 197.06; Found, 197.10

Synthesis of compound 9. Compound 9 was prepared according to reported protocol[4]. ^{1}H NMR (500 MHz, DMSO-D6): δ= 7.41 – 7.22 (m, 2H), 7.12 (dd, J = 5.7, 2.7 Hz, 1H), 7.06 – 6.98 (m, 2H), 6.94 (dd, J = 5.7, 0.8 Hz, 1H), 5.77 (d, J = 2.7 Hz, 1H), 4.58 (s, 2H), 2.03 (s, 3H). ^{13}C NMR (126 MHz, DMSO-D6): δ=167.2, 150.0, 149.3, 145.7, 144.7, 126.3 (d, J = 6.5 Hz), 121.5 (d, J = 12.4 Hz), 80.1, 68.3, 59.1, 22.1. ESI-MS: Calcd. For m/z, $C_{13}H_{14}NO_2$+ [M+H]+ 216.10; Found, 216.20

2.3.2 Synthesis of pro-fluorophores 1-2

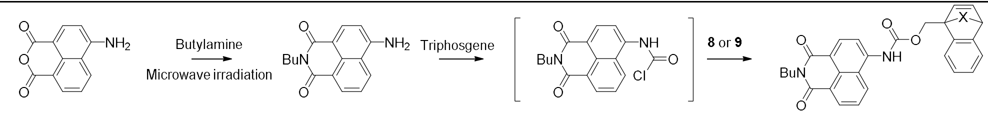

10

PF 1 (X = O)
PF 2 (X = NAc)

Synthesis of 4-amino-N-butyl-1,8-naphthalimide[5] (N-butyl Lucifer, Compound 10). Compound **10** was prepared according to reported protocol. ^{1}H NMR (500 MHz, CD_3OD): δ= 8.61 – 8.38 (m, 2H), 8.27 (d, J = 8.4 Hz, 1H), 7.63 (dd, J = 8.4, 7.3 Hz, 1H), 6.87 (d, J = 8.4 Hz, 1H), 4.23 – 3.87 (m, 2H), 1.80 – 1.54 (m, 2H), 1.52 – 1.38 (m, 2H), 0.99 (t, J = 7.4 Hz, 3H).^{13}C NMR (126 MHz, CD_3OD): δ=166.3, 165.8, 154.6, 135.6, 132.6, 131.5, 130.2, 125.3, 123.4, 121.2, 109.7, 40.8, 31.4, 21.4, 14.2. ESI-MS: Calcd. For m/z, $C_{16}H_{17}N_2O_2$+ [M+H]+ 269.13; Found, 269.11

Synthesis of Profluorophore 1 (PF 1). Compound **10** (25 mg, 0.093 mmol) and DMAP (35 mg, 0.28 mmol) were dissolved in anhydrous DCM (2 mL) under nitrogen atmosphere. The reaction was cooled to 0°C using an ice bath before triphosgene solution (17 mg, 0.055 mmol) in toluene was added dropwise. The reaction mixture was allowed warm to room temperature and stirred for 4 h. The reaction mixture was reduced to dryness by bubbling with argon before the residue was redissolved in anhydrous DCM and cooled to 0 °C (2

---

[4] M.H. Xu, J. Tu, R. M. Franzini, *Chem. Commun.*, **2017**, *53, 6271*

[5] L. Cui *et. al.*, *Chem. Commun.*, **2014,**50, 1485-1487

mL). Compound **7** (19 mg, 0.11 mmol) in 1 mL of anhydrous DCM was added dropwise. The reaction was allowed to return to room temperature and stirred overnight under nitrogen atmosphere. The reaction was quenched with water and extracted with DCM. The resulting organic layer was dried over $MgSO_4$, filtered and removed in vacuo to afford the crude product as a yellow oil. Purification was performed (0-20% EtOAc in Hexane) to afford **PF 1** as a yellow solid (11 mg, 25% yield). ^{1}H NMR (500 MHz, CD_3OD): δ= 8.57 (dd, J = 7.2, 1.2 Hz, 1H), 8.53 (dd, J = 8.5, 1.2 Hz, 2H), 8.25 (d, J = 8.2 Hz, 1H), 7.79 (dd, J = 8.5, 7.2 Hz, 1H), 7.38 – 7.24 (m, 2H), 7.21 – 7.11 (m, 1H), 7.06 – 6.94 (m, 3H), 5.77 (d, J = 1.9 Hz, 1H), 5.17 (d, J = 12.5 Hz, 1H), 5.09 (d, J = 12.5 Hz, 1H), 4.18 – 4.10 (m, 2H), 1.75 – 1.65 (m, 2H), 1.49 – 1.38 (m, 2H), 1.00 (t, J = 7.4 Hz, 3H). ^{13}C NMR (126 MHz, CD_3OD): δ= 165.7, 165.2, 155.8, 151.7, 149.1, 143.1, 142.1, 133.1, 132.3, 130.2, 129.8, 127.6, 126.3, 126.1, 125.8, 124.0, 121.3, 121.0, 120.7, 119.8, 119.1, 92.9, 83.6, 63.4, 41.0, 31.3, 21.3, 14.2. ESI-HRMS: Calcd. For m/z, $C_{28}H_{25}N_2O_5$+ [M+H]+ 469.1758; Found, 469.1753

Synthesis of Profluorophore 2 (PF 2). Compound **10** (25 mg, 0.093 mmol) and DMAP (35 mg, 0.28 mmol) were dissolved in anhydrous DCM (2 mL) under nitrogen atmosphere. The reaction was was cooled to 0°C using an ice bath before triphosgene solution (17 mg, 0.055 mmol) in toluene was added dropwise. The reaction mixture was allowed warm to room temperature and stirred for 4 h. The reaction mixture was reduced to dryness by bubbling with argon before the residue was redissolved in anhydrous DCM and cooled to 0 °C (2 mL). Compound 8 (24 mg, 0.11 mmol) in 1 mL of anhydrous DCM was added dropwise. The reaction was allowed to return to room temperature and stirred overnight under nitrogen atmosphere. The reaction was quenched with water and extracted with DCM. The resulting organic layer was dried over $MgSO_4$, filtered and removed in vacuo to afford the crude product as a yellow oil. Purification was performed (0-20% EtOAc in Hexane) to afford **PF 2** as a yellow solid (17 mg, 36% yield). ^{1}H NMR (500 MHz, CD_3OD): δ= 8.61 – 8.50 (m, 3H), 8.27 (d, J = 8.2 Hz, 1H), 7.80 (dd, J = 8.6, 7.3 Hz, 1H), 7.43 – 7.32 (m, 2H), 7.18 – 7.09 (m, 2H), 7.03 (dd, J = 5.2, 3.0 Hz, 2H), 5.79 (d, J = 2.7 Hz, 1H), 5.54 (d, J = 11.7 Hz, 1H), 5.48 (d, J = 11.7 Hz, 1H), 4.18 – 4.11 (m, 2H), 2.00 (s, 3H), 1.75 – 1.65 (m, 2H), 1.50 – 1.39 (m, 2H), 1.00 (t, J = 7.4 Hz, 3H). ^{13}C NMR (126 MHz, CD_3OD): δ= 169.6, 165.7, 165.2, 155.8, 150.2, 149.9, 145.4, 144.3, 142.2, 133.0, 132.3, 130.2, 130.0, 127.6, 126.5, 126.3, 125.9, 124.0, 121.7, 121.3, 119.9, 119.1, 76.9, 68.4, 63.3, 41.0, 31.3, 22.6, 21.3, 14.2. ESI-HRMS: Calcd. For m/z, $C_{30}H_{28}N_3O_5$+ [M+H]+ 510.2023; Found, 510.2027

Synthesis of exo-5-Norbornene-Sulforhodamine B Probe (SRB Probe)

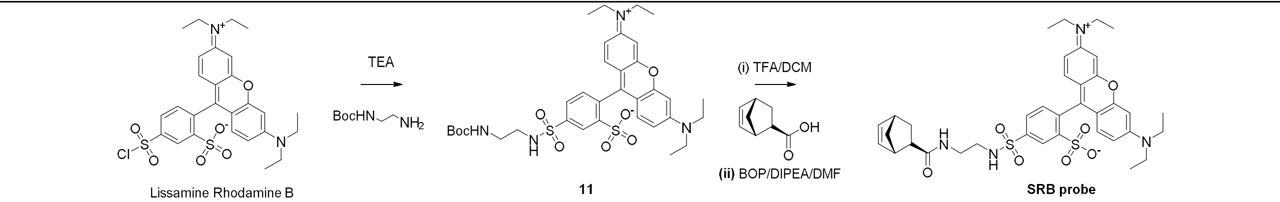

Lissamine Rhodamine B sulfonyl chloride

11

SRB probe

Synthesis of Compound 11[6]. Lissamine Rhodamine B sulfonyl chloride (50 mg, 0.087 mmol) was dissolved in anhydrous DCM (3 mL) and cooled on ice. To this solution was added N-Boc-ethylenediamine (28 mg, 0.17 mmol) and triethylamine (45 mg, 0.34 mmol). The reaction was stirred at room temperature overnight under nitrogen atmosphere. The reaction was quenched with $NaHCO_3$, extracted with DCM and washed with brine. The resulting organic layer was dried over $MgSO_4$, filtered and removed in vacuo to afford the crude product. Purification was performed (0-10% MeOH in DCM) to afford the compound **10** as a purple solid (42 mg, 69%

[6] P. Schmidt, L. Zhou, K. Tishinov, K. Zimmermann, D. Gillingham. *Angew. Chem. Int. Ed.* **2014**, 53, 10928 –10931

yield). ^{1}H NMR (500 MHz, DMSO-D6): δ= 8.40 (s, 1H), 8.01 (s, 1H), 7.92 (d, J = 7.9 Hz, 1H), 7.47 (d, J = 7.9 Hz, 1H), 7.06 – 6.96 (m, 3H), 6.94 (d, J = 2.3 Hz, 2H), 3.64 (dt, J = 13.0, 7.1 Hz, 8H), 3.02 (m, 2H), 2.89 (m, 2H), 1.37 (s, 9H), 1.21 (t, J = 7.1 Hz, 12H). ^{13}C NMR (126 MHz, DMSO-D6): δ=157.5, 157.1, 155.5, 155.0, 148.1, 141.5, 133.0, 132.7, 130.6, 126.5, 125.6, 113.6, 113.5, 95.4, 77.8, 45.2, 28.2, 12.4. ESI-MS: Calcd. For m/z, $C_{34}H_{45}N_4O_8S_2$+ [M+H]+ 701.27; Found, 701.30

2.3.3 Synthesis of SRB Probe

Compound 11 (10 mg, 0.014 mmol) was treated with 10% TFA/DCM (2 mL) for 2 h. The solvent was removed in vacuo. The free amine intermediate was re-dissolved in anhydrous DMF (1 mL), to which exo-5-norbornene-2-carboxylic acid (2 mg, 0.014 mmol), BOP (6.3 mg, 0.014 mmol), and DIPEA (7.3 m, 0.057 mmol) were added. The reaction mixture was diluted with EtOAc, washed with saturated brine and then dried over $MgSO_4$. The reaction mixture was filtered and the filtrate was collected and removed in vacuo. Purification was performed (0-10% MeOH in DCM) using column chromatography to afford **SRB Probe** as a purple solid (7 mg, 59% yield). ^{1}H NMR (500 MHz, DMSO-D6): δ= 8.42 (d, J = 2.0 Hz, 1H), 8.03 (t, J = 6.0 Hz, 1H), 7.98 – 7.90 (m, 2H), 7.48 (d, J = 7.9 Hz, 1H), 7.07 – 6.96 (m, 4H), 6.94 (d, J = 2.0 Hz, 2H), 6.15 – 6.08 (m, 2H), 3.64 (dt, J = 12.2, 7.1 Hz, 8H), 3.15 (m, 2H), 2.95 – 2.87 (m, 2H), 2.82 (m, 2H), 2.05 – 1.98 (m, 1H), 1.81 – 1.70 (m, 1H), 1.62 (d, J = 7.9 Hz, 1H), 1.21 (t, J = 7.1 Hz, 12H). ^{13}C NMR (126 MHz, DMSO-D6): δ= 174.9, 157.5, 157.1, 155.0, 148.1, 141.4, 137.7, 136.2, 133.1, 132.7, 130.7, 127.8, 126.5, 125.7, 124.3, 119.0, 113.6, 113.5, 109.7, 95.4, 46.8, 45.7, 45.2, 43.0, 42.1, 41.0, 38.7, 30.7, 29.8, 12.4. ESI-HRMS: Calcd. For m/z, $C_{37}H_{45}N_4O_7S_2$+ [M+H]+ 721.2724; Found, 721.2729

2.3.4 Synthesis of prodrugs

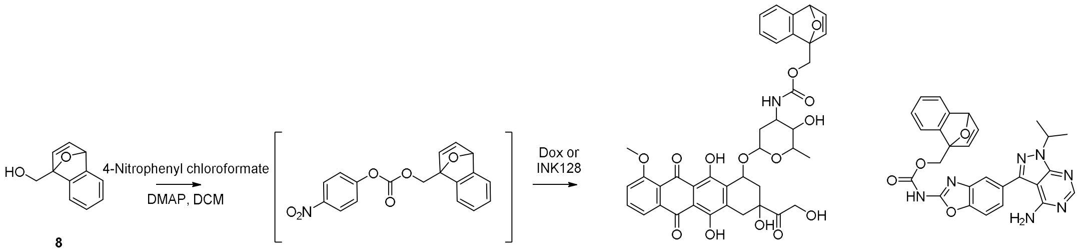

Synthesis of Pro-Dox. Pro-Dox was prepared using a modified reported protocol[4]. Compound **8** (10 mg, 0.057 mmol) and DMAP (14 mg, 0.11 mmol) was dissolved in anhydrous DCM (1 mL) and stirred at 0 °C. To the solution was added 4-nitrophenyl chloroformate (17 mg, 0.086 mmol) dissolved in 1 mL of DCM dropwise. The reaction was allowed to return to room temperature and stirred in the dark overnight. The reaction mixture was quenched with ice and extracted with DCM. The combined organic layer was washed with water and brine until no more yellow color was observed in the organic phase, dried over $MgSO_4$ and removed in vacuo to afford the carbonate intermediate as yellow solid in a yield of 15 mg (77%). Doxorubicin hydrochloride (30 mg, 0.053 mmol) and DIPEA (57 mg, 0.44 mmol) dissolved in DMF and cooled on ice. To this mixture, the carbonate intermediate in DMF (15 mg in 1 mL) was added dropwise. The reaction mixture was stirred in the dark at room temperature overnight. The mixture was diluted with DCM, washed with water and brine. The organic layers were combined, dried with $MgSO_4$, filtered and removed in vacuo to afford red solid as crude product. Purification was carried out (0-10% MeOH in DCM) using column chromatography to afford the **Pro-Dox** as a red solid **(17 mg, 52%).** ^{1}H NMR (500 MHz, CD_2Cl_2): δ=14.05 (s, 1H), 13.29 (s, 2H), 8.03 (d, J = 7.7, 1H), 7.80 (t, J = 7.7 Hz, 1H), 7.42 (d, J = 8.6 Hz, 1H), 7.29 – 6.60 (m, 6H), 5.65 (br s, 1H), 5.59 – 5.50 (m, 1H),

5.26 (m, 1H), 4.90 (m, 1H), 4.80 – 4.73 (m, 3H), 4.64 (br s, 1H), 4.16 (d, J = 6.8 Hz, 1H), 4.04 (s, 3H), 3.86 (m, 1H), 3.67 (m, 1H), 3.29 (d, J = 18.9 Hz, 1H), 3.07 (d, J = 18.9 Hz, 1H), 2.90 (t, J = 4.7 Hz, 1H), 2.39 (d, J = 14.8 Hz, 1H), 2.18 (dd, J = 14.8, 4.1 Hz, 1H), 1.93 – 1.70 (m, 3H), 1.28 (d, J = 6.7 Hz, 3H). ^{13}C NMR (126 MHz, MeOD): δ= 214.7, 188.5, 188.2, 162.6, 157.7, 157.5, 156.4, 149.2, 145.8, 143.2, 137.2, 136.7, 135.9, 135.2, 126.1, 126.1, 121.9, 121.2, 120.6, 120.3, 112.7, 112.4, 102.2, 83.5, 77.4, 71.4, 70.1, 68.6, 65.7, 62.7, 57.1, 37.6, 34.1, 30.9, 17.3. ESI-HRMS: Calcd. For m/z, $C_{39}H_{37}NNaO_{14}$+ [M+Na]+ 766.2105; Found, 766.2106

Synthesis of Pro-INK128. Compound **8** (10 mg, 0.057 mmol) and DMAP (14 mg, 0.11 mmol) was dissolved in anhydrous DCM (1 mL) and stirred at 0 ^{o}C. To the solution was added 4-nitrophenyl chloroformate (17 mg, 0.086 mmol) dissolved in 1 mL of DCM dropwise. The reaction was allowed to return to room temperature and stirred in the dark overnight. The reaction mixture was quenched with ice and extracted with DCM. The combined organic layer was washed with water and brine until no more yellow color was observed in the organic phase, dried over $MgSO_4$ and removed in vacuo to afford the carbonate intermediate as yellow solid in a yield of 15 mg (77%). INK128 (18 mg, 0.058 mmol) and DIPEA (57 mg, 0.44 mmol) dissolved in DMF and cooled on ice. To this mixture, the carbonate intermediate in DMF (15 mg in 1 mL) was added dropwise. The reaction mixture was stirred in the dark at room temperature overnight. The mixture was diluted with DCM, washed with water and brine. The organic layers were combined, dried with $MgSO_4$, filtered and removed in vacuo to afford white solid as crude product. Purification was carried out (0-10% MeOH in DCM) using column chromatography to afford the **Pro-INK128** as a white solid (8 mg, 27%). ^{1}H NMR (500 MHz, DMSO-d_6) δ 8.24 (s, 1H), 7.55 (br app. s, 2H), 7.39 – 7.22 (m, 3H), 7.19 – 7.09 (m, 1H), 7.04 – 6.91 (m, 3H), 5.78 (br s, 1H), 5.07 (m, 1H), 5.02 – 4.79 (br app. s, 2H), 1.52 (s, 3H), 1.51 (s, 3H). ^{13}C NMR (126 MHz, DMSO-d_6) δ 202.4, 162.8, 158.6, 153.7, 151.1, 138.0, 125.1, 120.3, 98.0, 81.8, 70.3, 48.6, 36.3, 31.2, 22.3. ESI-HRMS: Calcd. For m/z, $C_{27}H_{24}N_7O_4$+ $[M+H]^+$ 510.1884; Found, 510.1889

3. LTzC-mediated deprotection of tetrazine-sensitive probes

3.1 Profiling of LTzC-mediated deprotection of tetrazine-sensitive fluorescent probes

Deprotection of **PF 1** or **PF 2** by LTzC or non-targeting LTzC was performed in biocompatible conditions (10% FBS in DMEM media at 37 ^{o}C) in 96-well flat-bottom plates. 1 mM stock solutions of LTzCs, profluorophores and N-butyl Lucifer were prepared in biological grade DMSO. Initial fluorescent profiling control studies was performed to assess fluorescence activities of DMSO, **PF 1** or **PF 2**, or LTzCs / Tz 3, and N-butyl Lucifer. **PF 1** or **PF 2** and LTzC displayed no interfering fluorescence activity. Time-course kinetic profiling studies were carried out over 24 h. The final composition comprised 1 μL of 1 mM LTzC (end conc. = 10 μM) and/or 1 μL of 1 mM profluorophore (end conc. = 10 μM) in 98 μL of DMEM. Activation of **PF 1** or **PF 2** by tetrazine-mediated click-to-release to free N-butyl Lucifer by LTzC or non-targeting LTzC was monitored via detection of fluorescence emission (λ_{ex} = 440 nm; λ_{em} = 535nm) using a EnVision Multimode Plate Reader. All measurements were performed in triplicates, and analysed using GraphPad Prism.

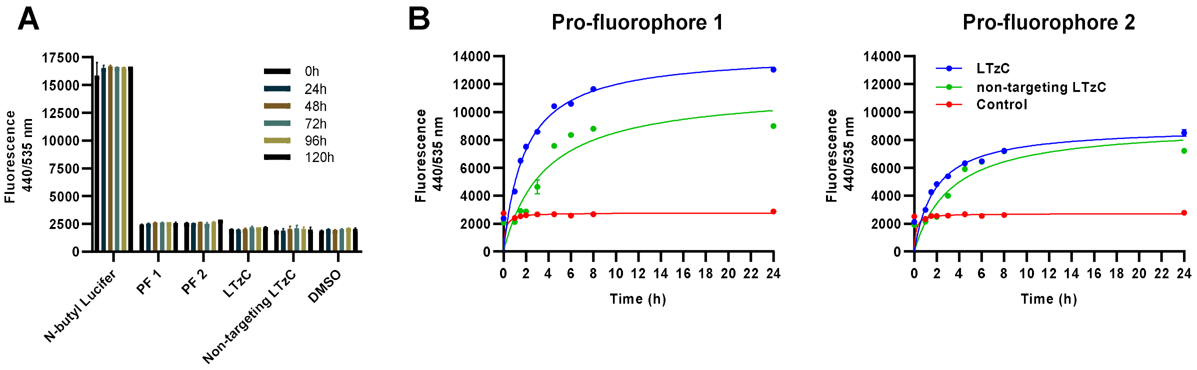

Figure S2. (A) Stability test via fluorescence profiling of free N-butyl Lucifer (10 μM), pro-fluorophores (10 μM), LTzC / non-targeting LTzC (10 μM), and blank (DMSO) in DMEM media over 5 d. (B) and (C) Time-course analysis of fluorescence intensity after incubation of PF 1 or PF 2 in biocompatible conditions (10 % serum in DMEM media) in the absence (control) or presence of LTzC or non-targeting LTzC. Error bars: ± SD from n = 3.

3.2 HPLC-MS analysis of LTzC-mediated deprotection of tetrazine-sensitive probes

HPCL-MS analysis of click-to-release of Lucifer Dye from **PF 1** or **PF 2** with LTzC was performed on Agilent 1260 Infinity II Prime LC System using InfinityLab Poroshell 120 EC-C18 column (3 X 100 mm; 2.7 micro). The mobile phase A was 0.1% Formic Acid in water and mobile phase B was acetonitrile. A gradient of 0-100% B ranging from 1-7 min was run at a flow rate of 1.0 mL/min. Stock solutions of PFs (10 mM) and LTzC (3 mM) in DMSO was prepared. Samples containing a final concentration of 10 μM and 12 μM of PFs and LTzC respectively were prepared. Samples were incubated at 37 °C and aliquots were taken at five time points (5 min, 30 min, 2 h, 4 h, 6 h and 24 h) and diluted by 5-fold with MeCN to quench the reaction and analysed by HPLC monitoring. The full composition of active species LTzC, **PF 1** or **PF 2**, Pyridazine (Pz) and Lucifer Dye were monitored at 254 nm channel. The conversion of **PF 1** or **PF 2** to Lucifer Dye was selectively monitored at 380 nm channel.

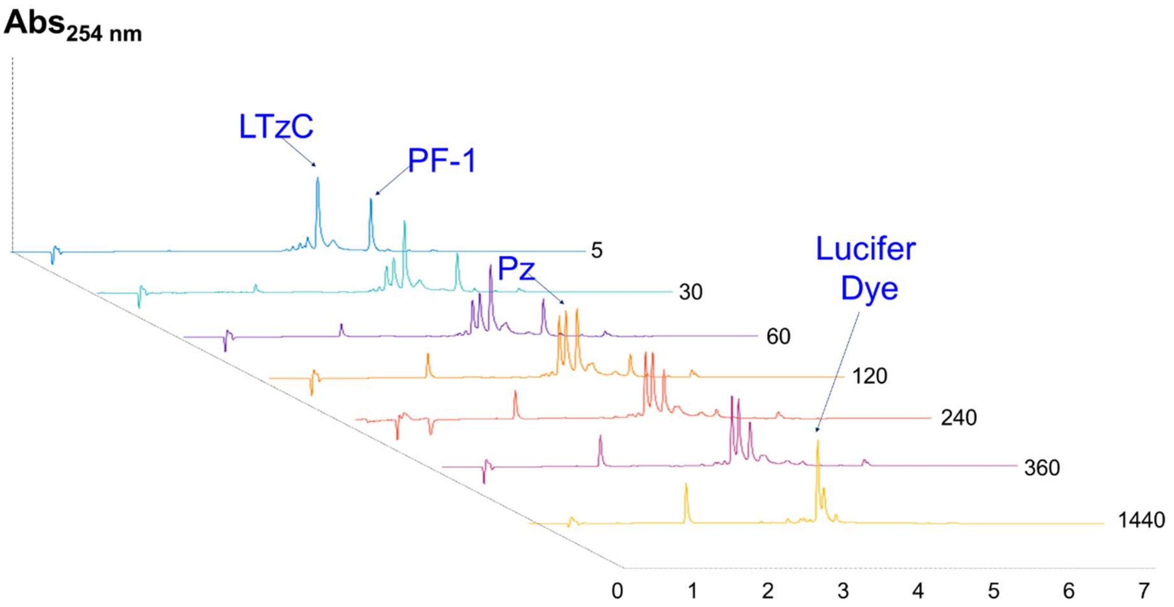

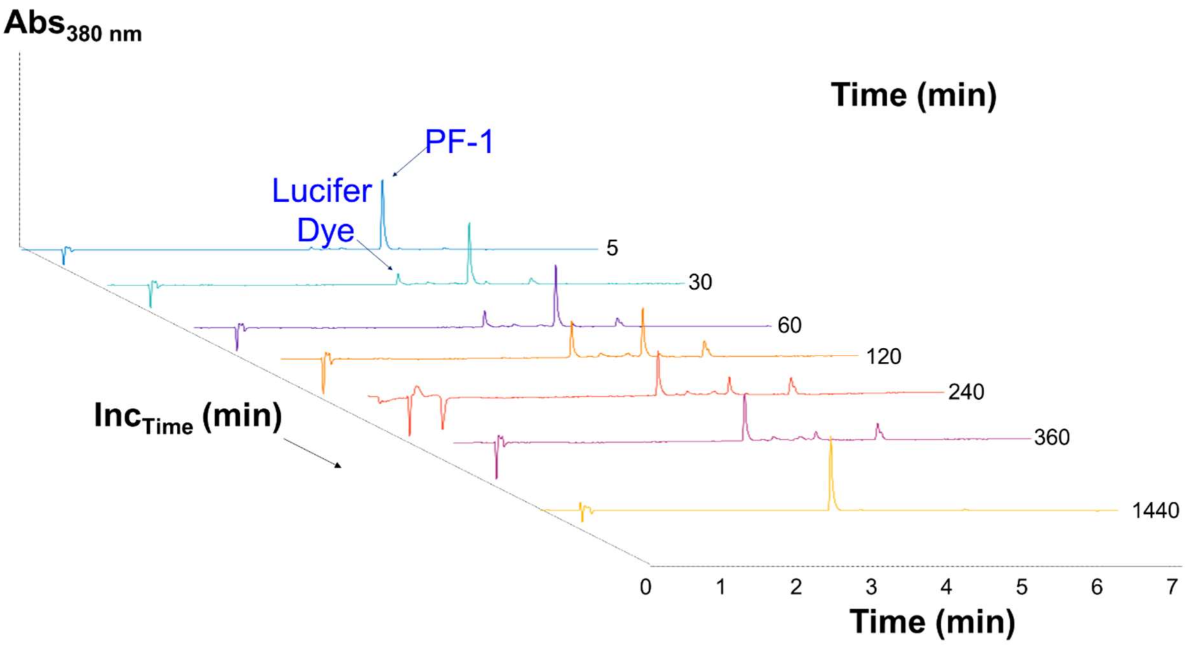

Figure S3. Analysis of reaction between **PF-1** and **LTzC** at different time points. Retention time for **LTzC**: 3.75 min; **Pyridazine** 3.61 min, **PF-1**: 4.38 min; **Lucifer Dye** 3.53 min.

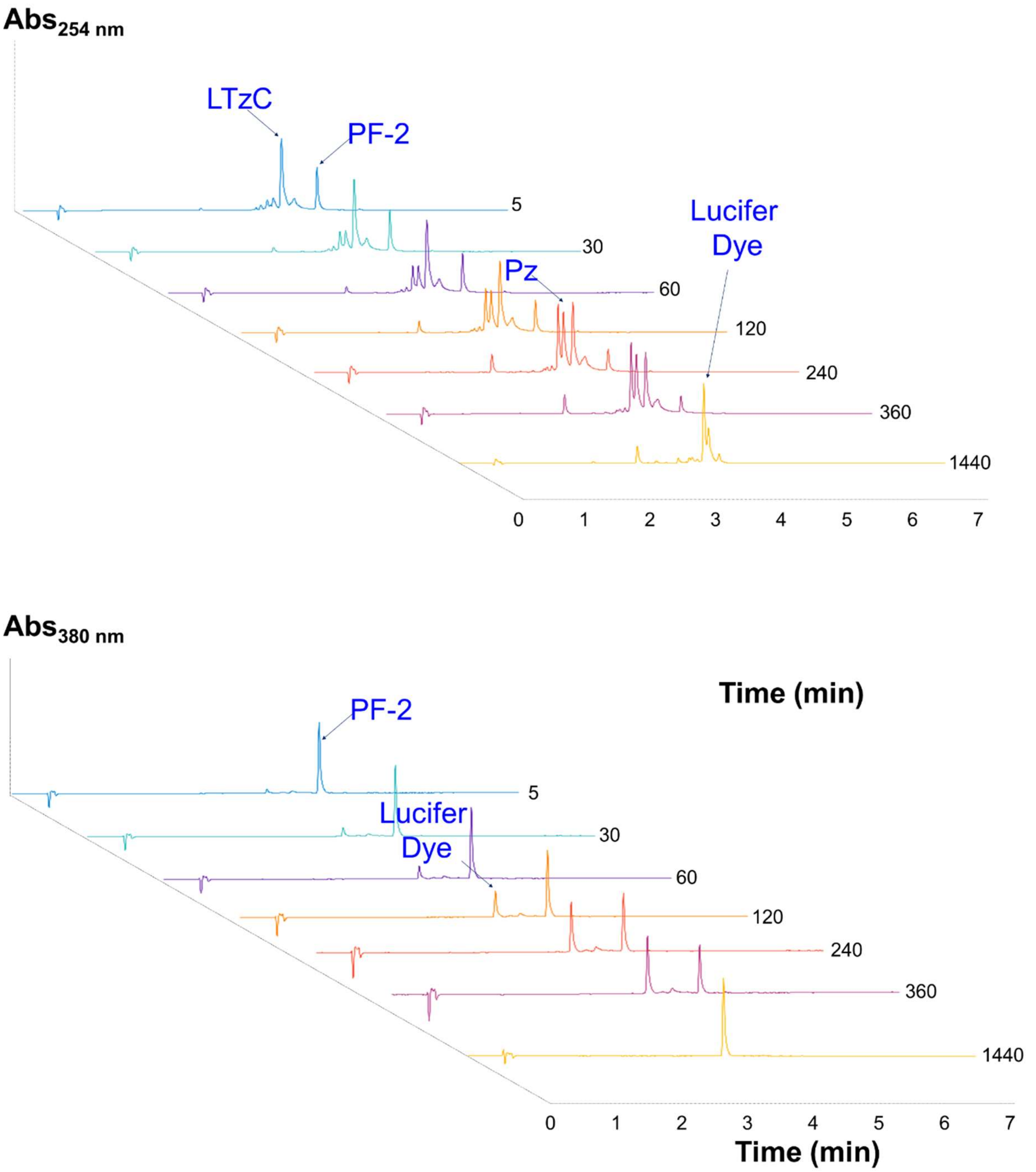

Figure S4. Analysis of reaction between **PF-2** and **LTzC** at different time points. Retention time for **LTzC**: 3.75 min; **Pyridazine** 3.61 min, **PF-**2: 4.24 min; **Lucifer Dye** 3.53 min.

3.3 Spectrophotometric analysis and calculation of cycloaddition reaction kinetics

The second-order rate constants of the cycloaddition between LTzC and benzonorbornadiene derivatives (compound **8** and **9)** were determined using spectrophotometric analysis. A solution of LTzC (30 μM) and compound **8** or **9** (600 μM, 450 μM, 300 μM and 150 μM) in 20% DMSO/PBS were prepared in 96-well plates and thoroughly mixed. The consumption of LTzC for tetrazine-mediated cycloaddition was monitored at λ_{abs} = 520 nm (local absorbance maximum of tetrazine moiety) at 37 °C using an EnVision Multimode Plate Reader in a time-course experiment. All experiments were performed in triplicate.

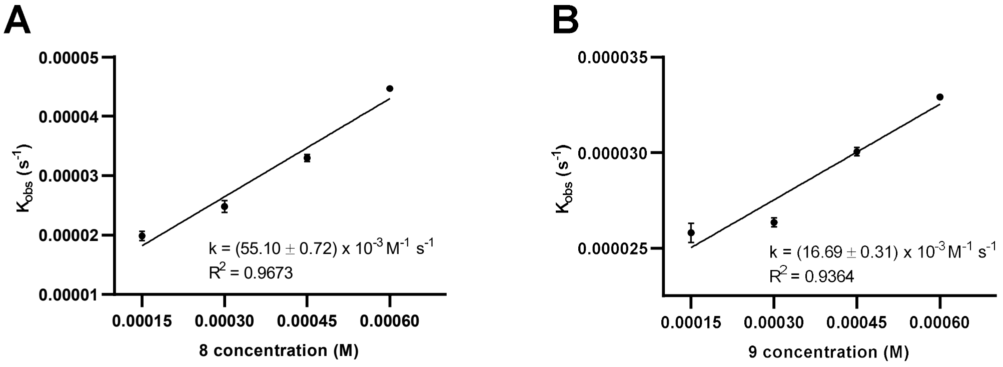

Figure S5. Second-order rate kinetics plots and calculated constants of the cycloadditive reactions between LTzC and (A) compound **8** or (B) compound **9** determined from the plots of pseudo-first order k_{obs} against concentration of **8**-**9** (150 to 600 μM). Error bars: ± SD from n = 3.

4. Biological studies

4.1 Cell culture

Human breast adenocarcinoma MDA-MB-231 and MCF-7 cells were cultured in Dulbecco's Modified Eagle Media (DMEM) supplemented with 10% foetal bovine serum and L-glutamine (2 mM) and maintained in a tissue culture incubator at 37 °C and 5% CO_2 environment.

4.2 PD-L1 expression validation studies for cell lines selection

PD-L1 positive (MDA-MD-231) and negative (MC7-7) cell lines were seeded (24000 cells per well) in 6 well plates. After 24 h, the media was removed and incubated with fresh DMEM media with or without IFN-γ (40 ng/mL). After 24h incubation, the plate was placed on ice, following by removal of the cell media. The wells were washed thrice with ice-cold PBS, followed by the addition of RIPA lysis buffer (Thermo Scientific) containing protease and phosphatase inhibitors (1.25 mM PMSF, 0.1% v/v aprotinin, 100 μM Na_2VO_4, 500 μM NaF). The cells were collected, incubated on ice for 10 min, and centrifuged at 4 °C (17000 x g, 10 min). The supernatant was collected and the total protein content was assessed using Pierce™ BCA Protein Assay Kit (Thermo Fischer Scientific). Appropriate dilution in RIPA buffer was performed to obtain 20 μg cell lysate for each respective samples. The supernatant was then treated with Laemmli Sample Buffer (Bio-Rad) at 95 °C for 5 min, loaded onto Mini-PROTEAN TGX precast gels (Bio-Rad), run in Mini-PROTEAN Tetra Cell tanks in

TGS buffer (pH 8.3) at 150 V for 45 min, transferred onto Trans-Blot Turbo Nitrocellulose membranes (Bio-Rad) using the Trans-Blot Turbo Transfer System (Bio-Rad). The membrane was blocked with blocking buffer for 1 h at room temperature, followed by incubation with the primary antibody (1:1000, Human PD-L1 Mab, R&D Systems Inc.) at 4°C for 24h. The membrane was washed twice with TBST, followed by incubation with the secondary antibody (1:1000, Goat Anti-Rabbit IgG HRP, R&D Systems Inc.) for 2 h. The membrane was washed thrice with TBST, followed by addition of Clarity ECL Western Blotting Substrates (Bio-Rad) before imaging in the ChemiDoc XRS+ Imaging System (Bio-Rad). Protein bands (PD-L1: ~50 kDA) were quantified using the ImageLab software v5.2 (Bio-Rad)

4.3 Effect of BMS202 and LTzC on PD-L1 expression

Flow cytometry quantification of surface PD-L1 Population. The PD-L1 expression (non-stimulated or IFN-gamma stimulated) and the expression after cytotoxic treatment (BMS-202 or LTzC) of the MDA-MB-231 cells was determined by flow cytometry. MDA-MD-231 cells were seeded (24000 cells per well) in 6 well plates. After 24 h, the media was removed and incubated with fresh DMEM media with or without IFN-γ (40 ng/mL) as control for 24 h, or with drug treatment (BMS-202 or LTzC) for 4h at two concentrations (3 μM or 1 μM). The cells was washed with PBS (3 times) and treated with trypsin solution in PE buffer for 5 min. 2 mL of DMEM media was added to quench the reaction and the cells were pipetting multiple times slowly to prepare single cell suspension. The samples were transferred to centrifuge tubes and centrifuged at 300 G for 5 min. The supernatant was discarded, to which primary anti-PD-L1 antibody (1:500; Extracellular Domain Specific anti-PD-L1 antibody D8T4X Rabbit mAb) in FACs buffer (5% BSA in PBS) was added and incubated on ice for 1 h. The cell suspension were centrifuged at 300 G for 5 min, and the supernatant was removed. The cell pellets were washed three times via centrifugation and supernatant removal. The secondary antibody (1:500, Alexa Fluor 488 Goat anti-Rabbit IgG) was added and incubated on ice for 1 h. Washing steps were carried out, followed by cell fixation (4% paraformaldehyde in PBS, 10 min at room temperature), and resuspension in FACs buffer. Rabbit IgG control was used for each condition as isotype control. The single cell suspension was analysed using a BD LSRFortessa™ X-20 Cell, and processed using FlowJo program.

Western Blot analysis of total PD-L1 Population. MDA-MB-231 cells were seeded (24000 cells per well) in 6 well plates. After 24 h, the media was removed and incubated with fresh DMEM media with or without IFN-γ (40 ng/mL) as control for 24 h, or with drug treatment (BMS-202 or LTzC) for 4h at two concentrations (3 μM or 1 μM). Sample preparation, western blot performance and analysis were performed as described as above in section 4.2.

5. PD-L1 imaging studies in breast cancer cells

5.1 LTzC-mediated labeling of PD-L1 positive cells using mode 'Track-&-Tag'

MDA-MB-231 and MCF-7 cells (24000 cells) were seeded in MatTek glass-bottom 6-well plate. After 24 h, the media was removed and incubated with fresh DMEM media with IFN-γ (40 ng/mL). After 24 h, the media was replaced with fresh media in the absence or presence of **LTzC** (3 μM) and incubated for 4 h. The media was removed from the wells, washed with PBS (3 times), and replaced with fresh media containing lissamine-norbornene probe (100 nM) for 24 h. Control wells were incubated with DMSO (0.1 % v/v). Subsequently, the media was removed, washed with PBS (3 times). PD-L1 immuno-labelling was performed using recommended reported protocol[7]. The cells was fixed using paraformaldehyde (4% v/v) for 15 min, and washed with PBS (3 times). Permeabilization of cells was performed using 0.1% Triton in PBS for 15 min, and washed with PBS (3 times). The cells was treated with blocking buffer (1X PBS, 5% goat serum) for 1 h. The blocking buffer was removed, washed with PBS (3 times), and primary antibody solution (**Ab1**; 1:500, Human PD-L1 Mab in PBS and 1% BSA) was added and incubated at 4 °C for 24 h. After washing with PBS (3 times), secondary antibody solution (**Ab2**, 1:500, Alexa Fluor 488 Goat anti-Rabbit IgG (H + L) in PBS and 1% BSa). The wells were washed with PBS, incubated with Alexa Fluor 647 Phalloidin (1:1000) and Hoerst 33342 (1:10000) in PBS for 20 min at room temperature. The wells were washed with PBS (3 times) and stored in PBS solution at 4 °C. Confocal imaging was performed using confocal inverted microscope Nikon A1R with a 40x air immersion objective (Plan Fluar 0.75 DIC N2). The image was acquired using NIS-Elements program in sequential mode using preconfigured settings for Hoerst 33342 (λ_{ex} = 350-410 nm; λ_{em} = 380-440 nm; blue; nuclei), Alexa Fluor 488 (λ_{ex} = 480-505 nm; λ_{em} = 510 - 540 nm; green; PD-L1 via IHC), Lissamine Rhodamine B (λ_{ex} = 560 - 600 nm; λ_{em} = 580-620 nm; red; PD-L1 via LTzC biorthogonal labelling) and Alexa Fluor 647 (λ_{ex} = 630 - 670nm; λ_{em} = 650-720nm; far red; actin)

Supp. Table 1: Labeling agents for cellular components and PD-L1.

Target	Labeling Agents	λ_{ex}	λ_{em}	Colour
Actin	Alexa Fluor Phalloidin 647	630-670	650-720	Far red
Nucleus	Hoerst 33342	350-410	380-440	Blue
PD-L1	**Immunolabeling:** Human PD-L1 Mab + Alexa Fluor 488 Goat anti-Rabbit IgG	480-505	510-540	Green
	**Bioorthogonal-labelling:** LTzC + SRB Probe	560-600	580-620	Red

[7] Cellular Oncology (2019) 42:237–242

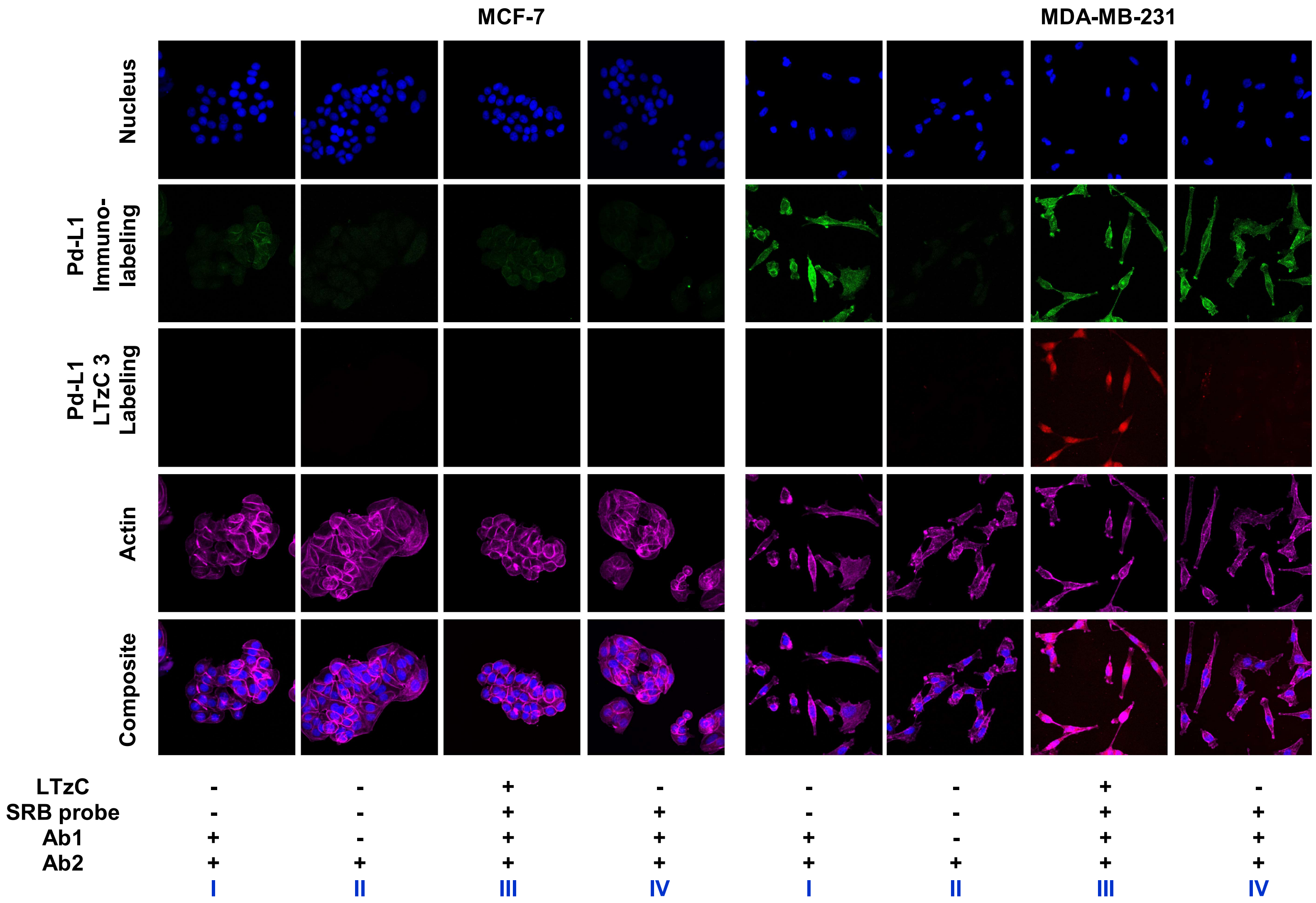

Figure S6. Confocal Imaging studies. Left panel: MCF-7 cells. Right panel: MDA-MB-231 cells. For each panel: (I) Control studies for immune-labeling of PD-L1. (**II**) Control studies for background labeling by Ab2. (iii) Dual PD-L1 labelling by immuno-labeling (ab1 + ab2) and biorthogonal labeling (**LTzC** + **SRB probe**). (IV) Control studies for background labeling by **SRB probe**.

5.2 Comparison of LTzC- and antibody-based PD-L1-labeling in live or fixed cells

PD-L1 labeling in live cells and fixed cells were assessed using LTzC-mediated labeling and antibody-based staining. MDA-MB-231 cells (24000 cells) were seeded in MatTek glass-bottom 6-well plate. After 24 h, the media was removed and incubated with fresh DMEM media with or without IFN-γ (40 ng/mL) for 24h. Cells were used directly for live imaging or fixed (15 min treatment with 4% PFA followed by permeabilization with 0.1% Triton in PBS for 10 min) for fixed-cell imaging.

Antibody-labeling. The wells were treated with extracellular specific or non-domain-specific primary PD-L1 antibody solution (Ab1; 1:500, 10% FBS / DMEM media) was added and incubated at 37 °C for 30 min. The media was removed, washed with PBS (3 times), and replaced with fresh media containing the secondary antibody solution (Ab2, 1:500; 10% FBS / DMEM media) at 37 °C for 30 min.

LTzC-mediated labeling. The samples for LTzC-mediated labeling was replaced with fresh media in the presence of **LTzC** (3 μM) and incubated for 4 h. The media was removed from the wells, washed with PBS (3 times), and replaced with fresh media containing SRB probe (100 nM) for 24 h. Subsequently, the media was removed, washed with PBS (3 times), and all samples were incubated with Hoerst 33342 (1:10000) in media for 20 min at 37 °C. Subsequently, the media was removed, washed with PBS (3 times), and fresh media was added. The image was acquired and processed as described above in **section 5.1**.**The fixed-cells treated with IFN-γ and labeled with LTzC-SRB probe system was further co-labeled via antibody-staining for additional validation of PD-L1 labeling. This specific sample was imaged with a 100x oil immersion objective.*

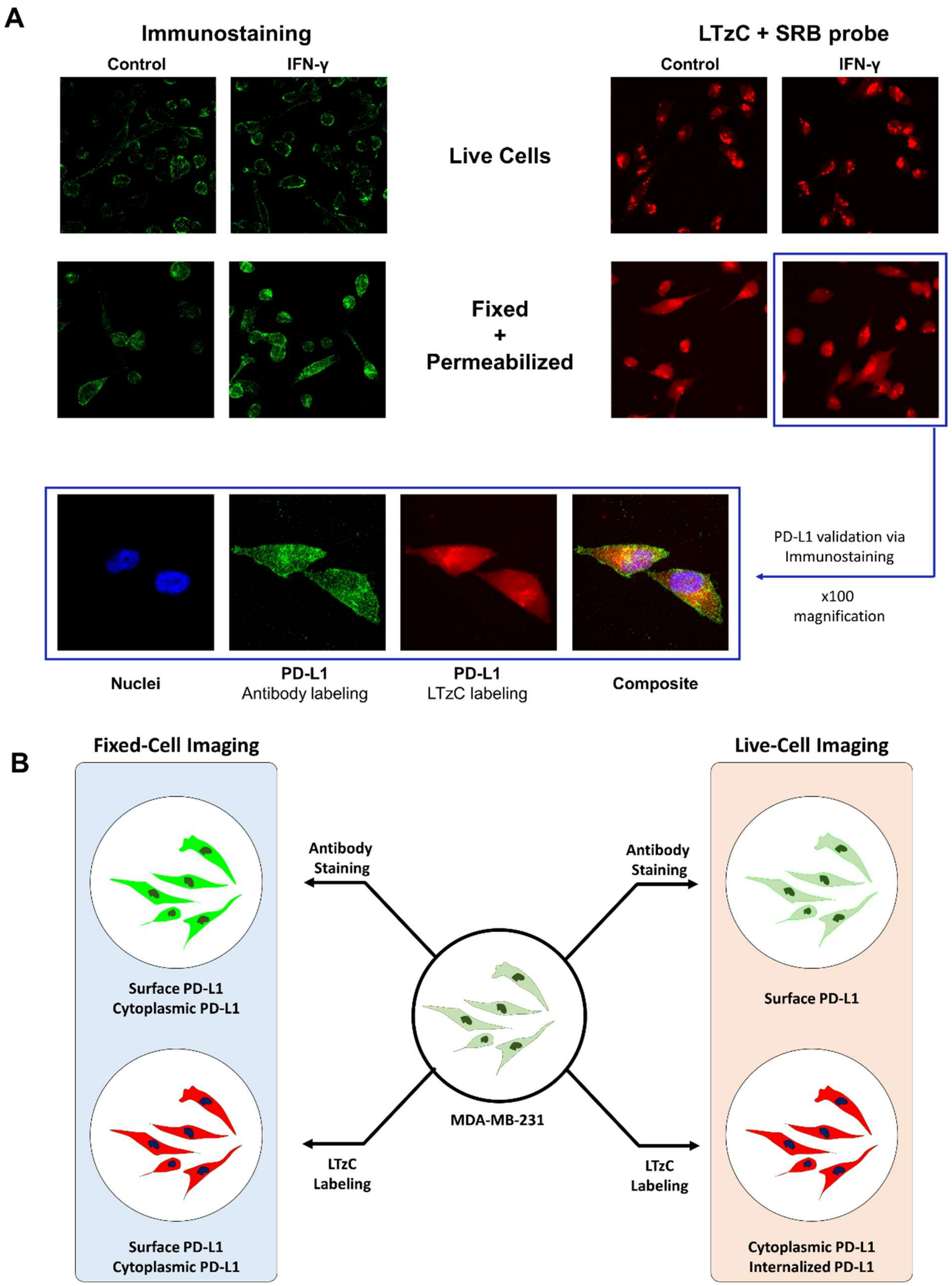

Figure S7. (A) Confocal Imaging studies. Top panel: Live MDA-MB-231 cells. Bottom panel: Fixed MDA-MB-231 cells. For each panel: (I) Immuno-labeling using anti-PD-L1 antibody. (ii) PD-L1 labeling by biorthogonal labeling (**LTzC** + **SRB probe**). The experiments were performed with or without IFN-γ treatment. (B) Mapping of different PD-L1 receptor population by LTzC-mediated and antibody-mediated labeling.

6. Biocompatibility and cell viability studies

Cytotoxicity of LTzC, control constructs (non-targeting LTzC, lipoic acid, compound 7), pro-drugs (pro-doxorubicin and pro-INK128) and parent drugs (doxorubicin and INK128) were assessed in PD-L1 positive (MDA-MD-231) and negative (MC7-7) cell lines via dose response studies. Cells were seeded in a 96-well plate (1000 cells per well), incubated or 24 h, followed by replacement with fresh media with or without IFN-γ (40 ng/mL). After another 24 h incubation, the media was replaced with fresh media containing respective LTzCs (10 - 0.001 μM) and incubated for 5 d. Control wells were incubated with DMSO (0.1 % v/v). Experiments were performed in triplicates. PrestoBlue™ cell viability reagent (10 % v/v) was added to each well and the plate incubated for 90 min. Fluorescence emission was detected using a PerkinElmer Victor multilabel reader (λ_{ex} = 540nm; λ_{em} = 590nm). All conditions were normalized to the untreated cells (100%) and analysis was performed using GraphPad Prism.

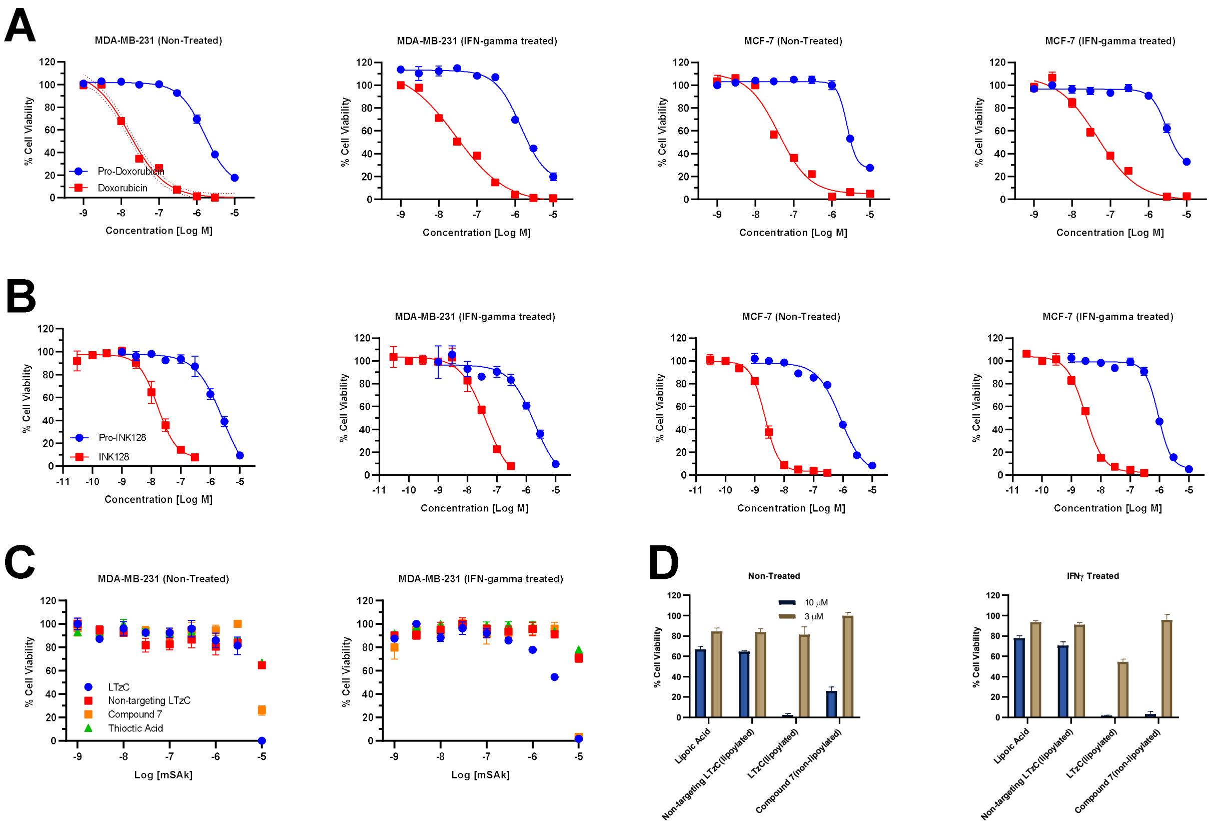

Figure S8. (A) Dose response studies of Pro-Dox and doxorubicin in MDA-MB-231 and MCF-7 cells. (B) Dose response studies of Pro-INK128 and INK128 in MDA-MB-231 and MCF-7 cells. (C) Dose response studies of **LTzC** (blue) and control constructs **non-targeting LTzC** (red), **lipoic acid** (green) and compound **7** (orange) in MDA-MB-231 without and with IFN-gamma stimulation. Error bars: ± SD from n = 3. (D) Antiproliferative effects of **lipoic acid**, **non-targeting LTzC**, **LTzC** and compound **7** against MDA-MB-231 without and with IFN-gamma stimulation at 3 and 10 μM. Error bars: ± SD from n = 3.

7. LTzC-mediated pro-drug activation in breast cancer cells

MDA-MB-231 and MCF-7 cells were seeded in a 96-well plate (1000 cells per well), incubated or 24 h, followed by replacement with fresh media with or without IFN-γ (40 ng/mL). After 24 h, the media was replaced with fresh media containing respective LTzCs (3 μM) and incubated for 4 h. The media was removed from the wells, washed with PBS (3 times), and replaced with fresh media containing Pro-Dox (0.3 μM) or Pro-INK128 (0.1 μM) for 5 d. Control wells were incubated with DMSO (0.1 % v/v). Experiments were performed in triplicates. PrestoBlue™ cell viability reagent (10 % v/v) was added to each well and the plate incubated for 90 min. Fluorescence emission was detected using a EnVision Multimode Plate Reader (λ_{ex} = 540 nm; λ_{em} = 590 nm). All conditions were normalized to the untreated cells (100%) and analysis was performed using GraphPad Prism.

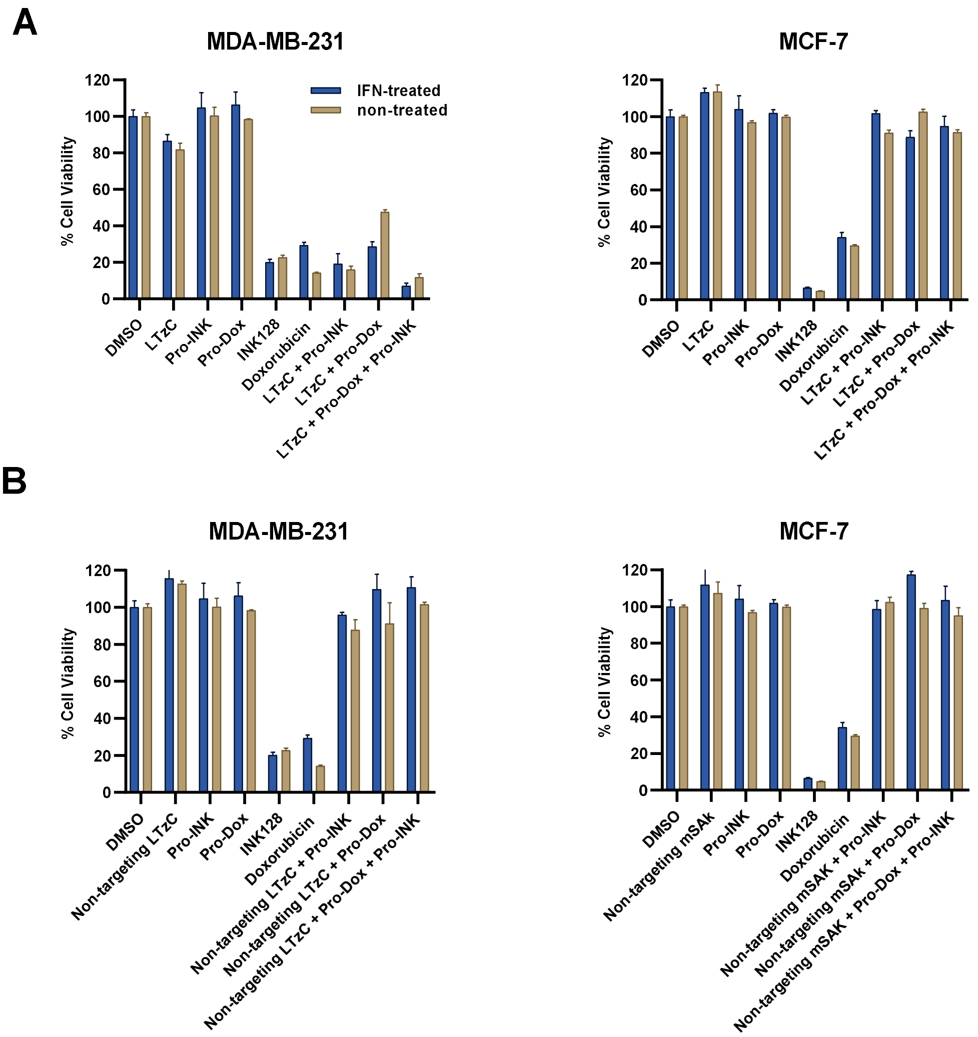

Figure S9. (A) Activation of Pro-Dox and/or Pro-INK128 by LTzC in MDA-MB-231 (PD-L1 positive) and MCF-7 cells (PD-L1 negative) (B) Activation of Pro-Dox and/or Pro-INK128 by non-targeting-LTzC in MDA-MB-231 (PD-L1 positive) and MCF-7 cells (PD-L1 negative). Negative controls: DMSO (untreated cells); LTzC or non-targeting LTzC (3 μM); Pro-Dox (0.3 μM) or Pro-INK128 (0.1 μM). Positive control: doxorubicin (0.3 μM), INK128 (0.1 μM). Treatment of cells with LTzC or non-targeting LTzC (3 μM) in combination with Pro-Dox (0.3 μM) and/or Pro-INK128 (0.1 μM). Error bars: ± SD from n = 3.

8. NMR spectra

Tetrazine 1

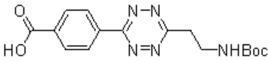

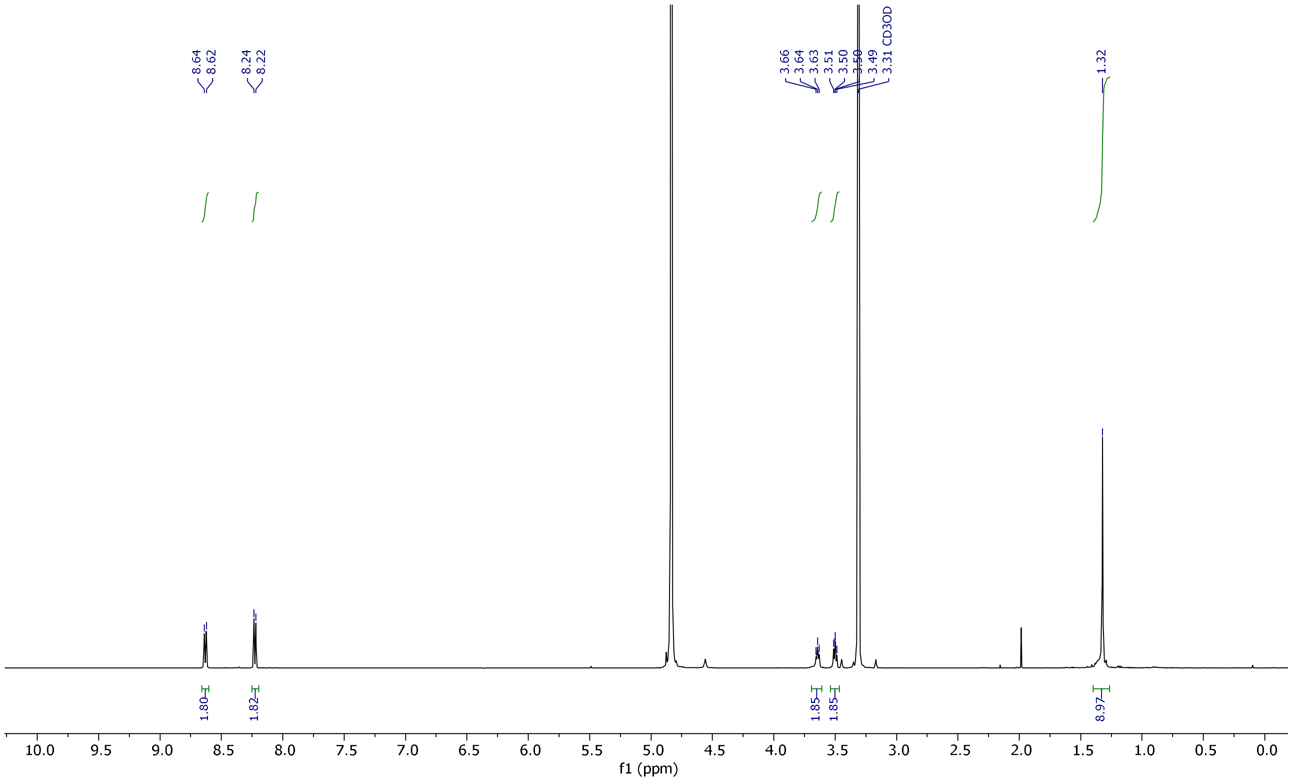

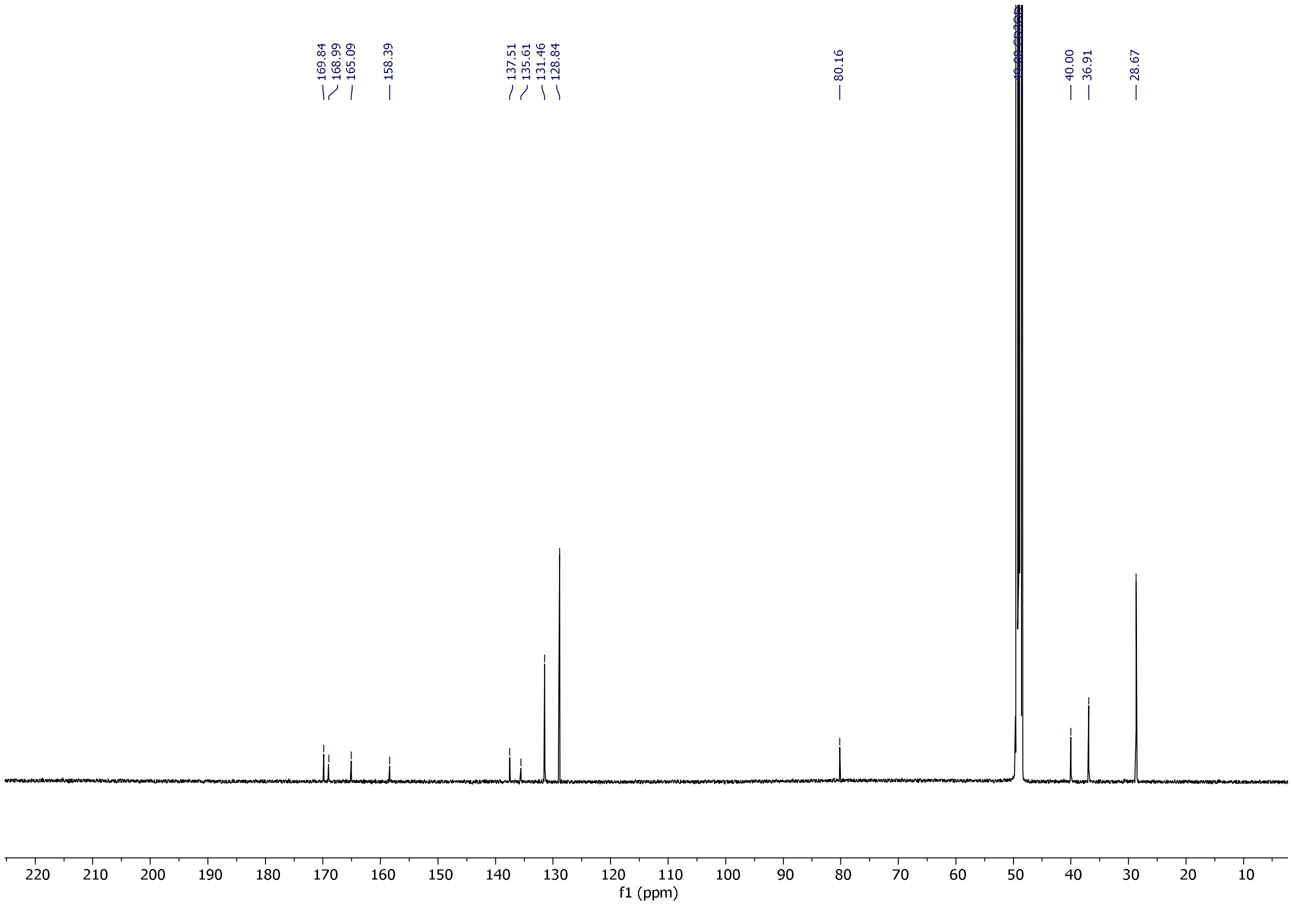

Compound 3

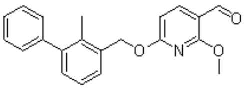

O
N
O
O

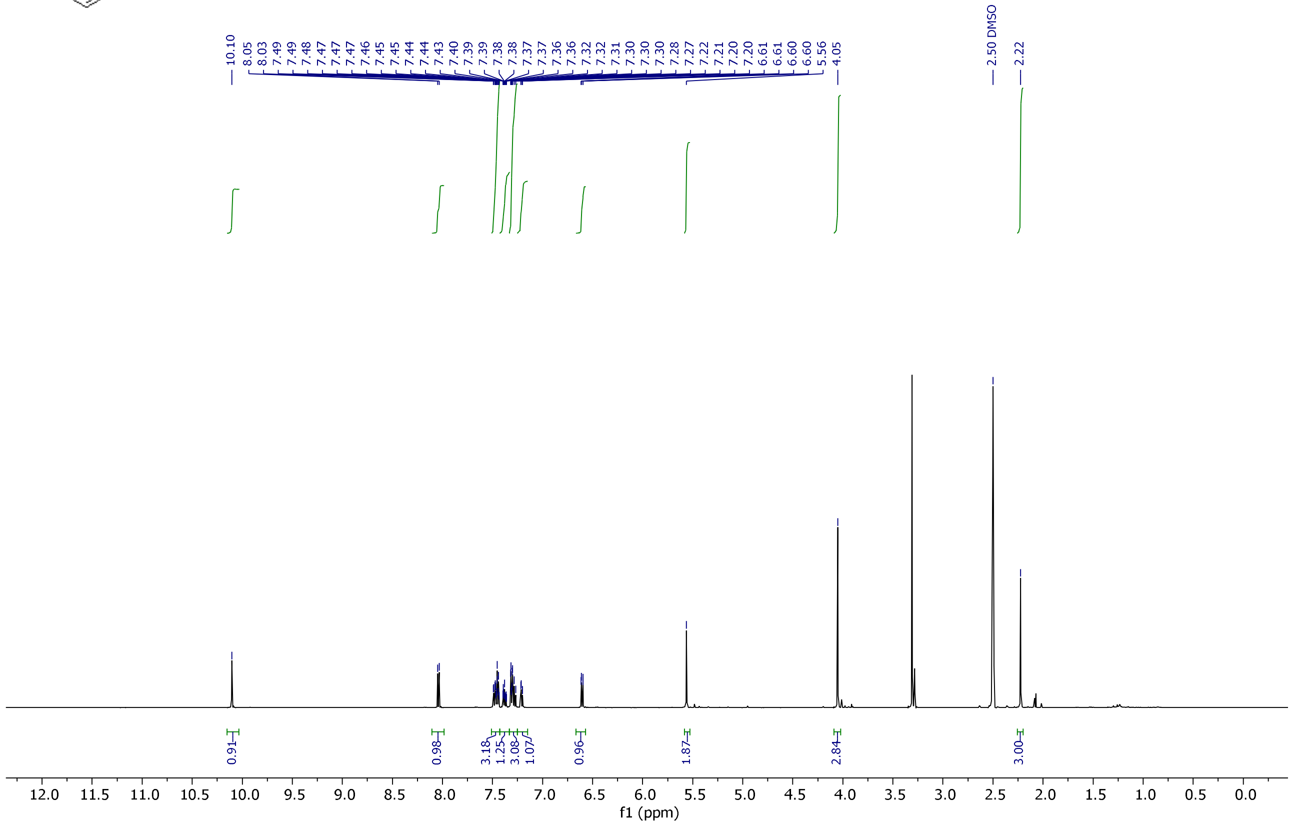

10.10
8.05
8.03
7.49
7.49
7.48
7.47
7.47
7.47
7.46
7.45
7.45
7.44
7.44
7.43
7.40
7.39
7.39
7.38
7.38
7.38
7.37
7.37
7.36
7.36
7.32
7.32
7.31
7.30
7.30
7.30
7.28
7.27
7.22
7.21
7.20
7.20
6.61
6.61
6.60
6.60
5.56
4.05
2.50 DMSO
2.22
0.91
0.98
3.18
1.25
3.08
1.07
0.96
1.87
2.84
3.00
12.0
11.5
11.0
10.5
10.0
9.5
9.0
8.5
8.0
7.5
7.0
6.5
6.0
5.5
5.0
4.5
4.0
3.5
3.0
2.5
2.0
1.5
1.0
0.5
0.0
f1 (ppm)

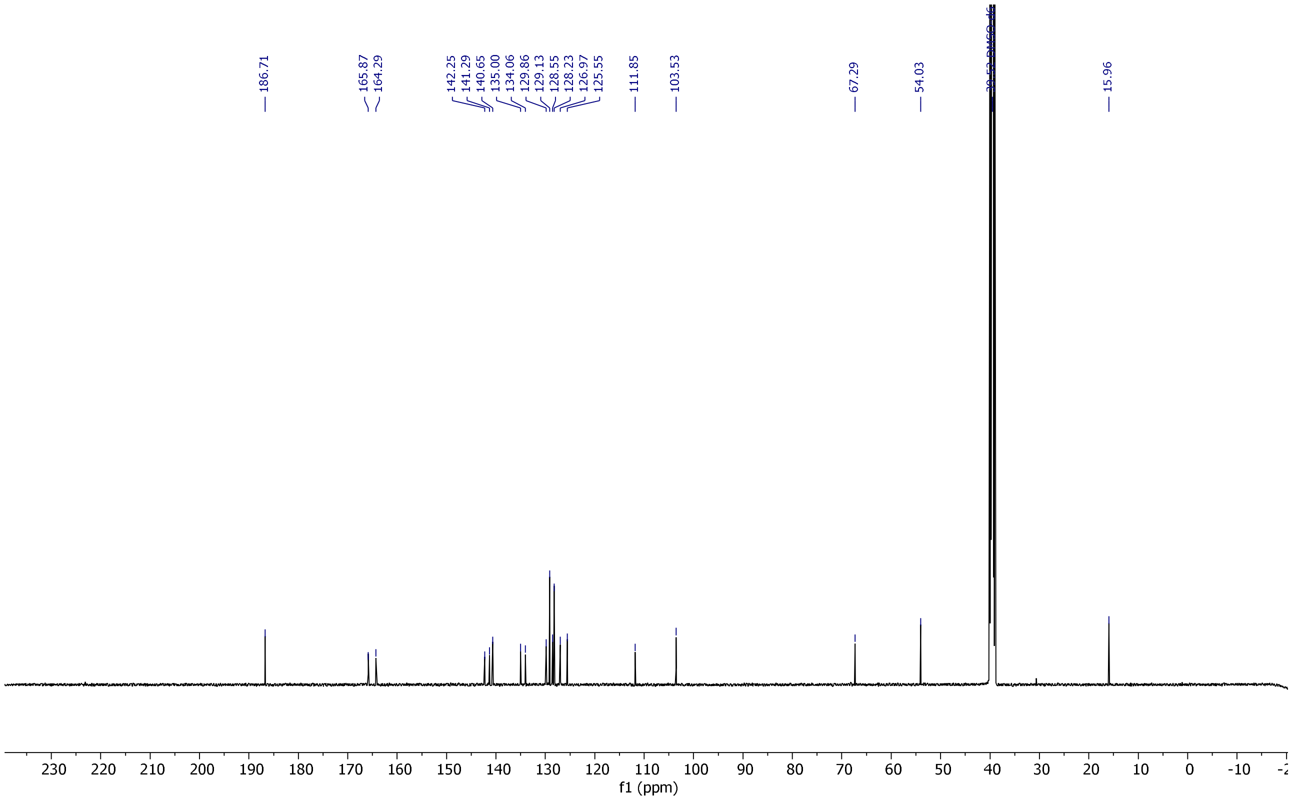

186.71
165.87
164.29
142.25
141.29
140.65
135.00
134.06
129.86
129.13
128.55
128.23
126.97
125.55
111.85
103.53
67.29
54.03
15.96
230
220
210
200
190
180
170
160
150
140
130
120
110
100
90
80
70
60
50
40
30
20
10
0
-10
f1 (ppm)

Compound 4

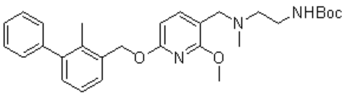

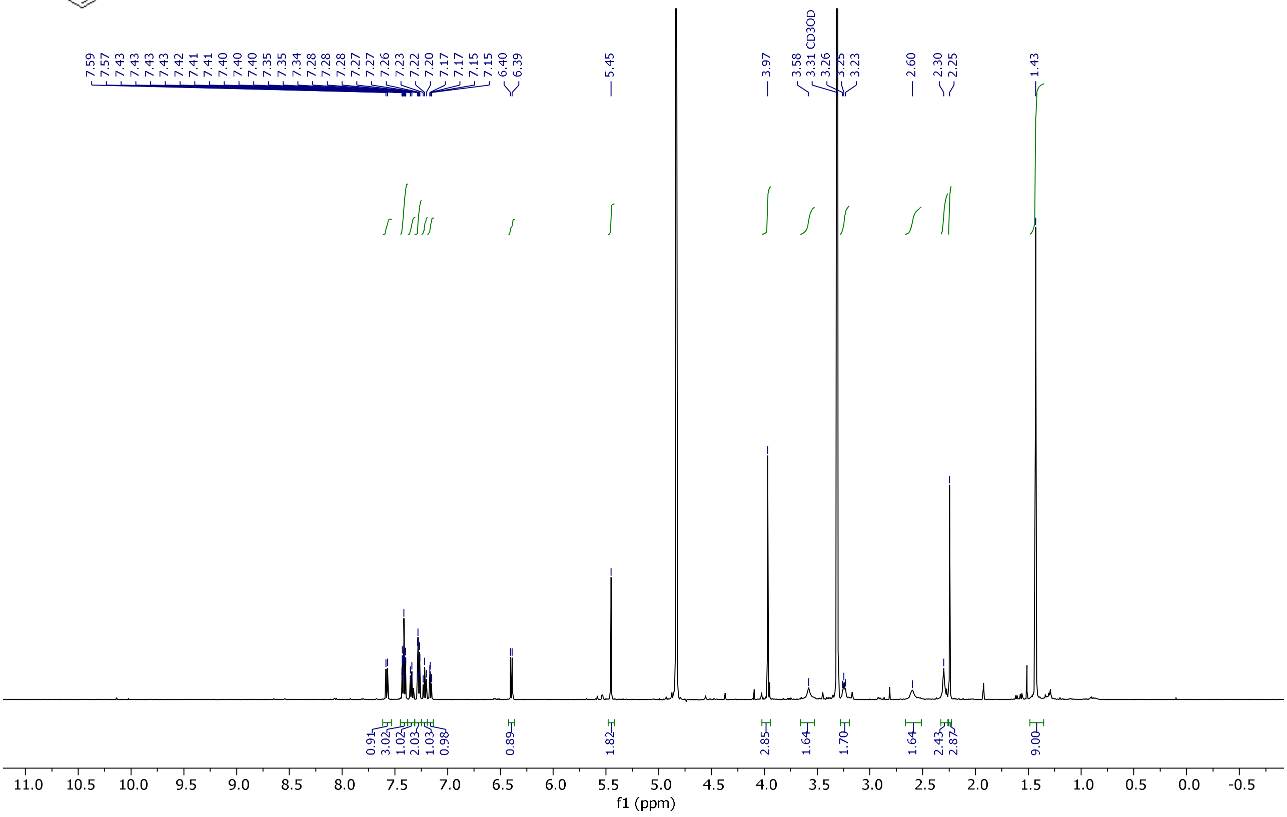

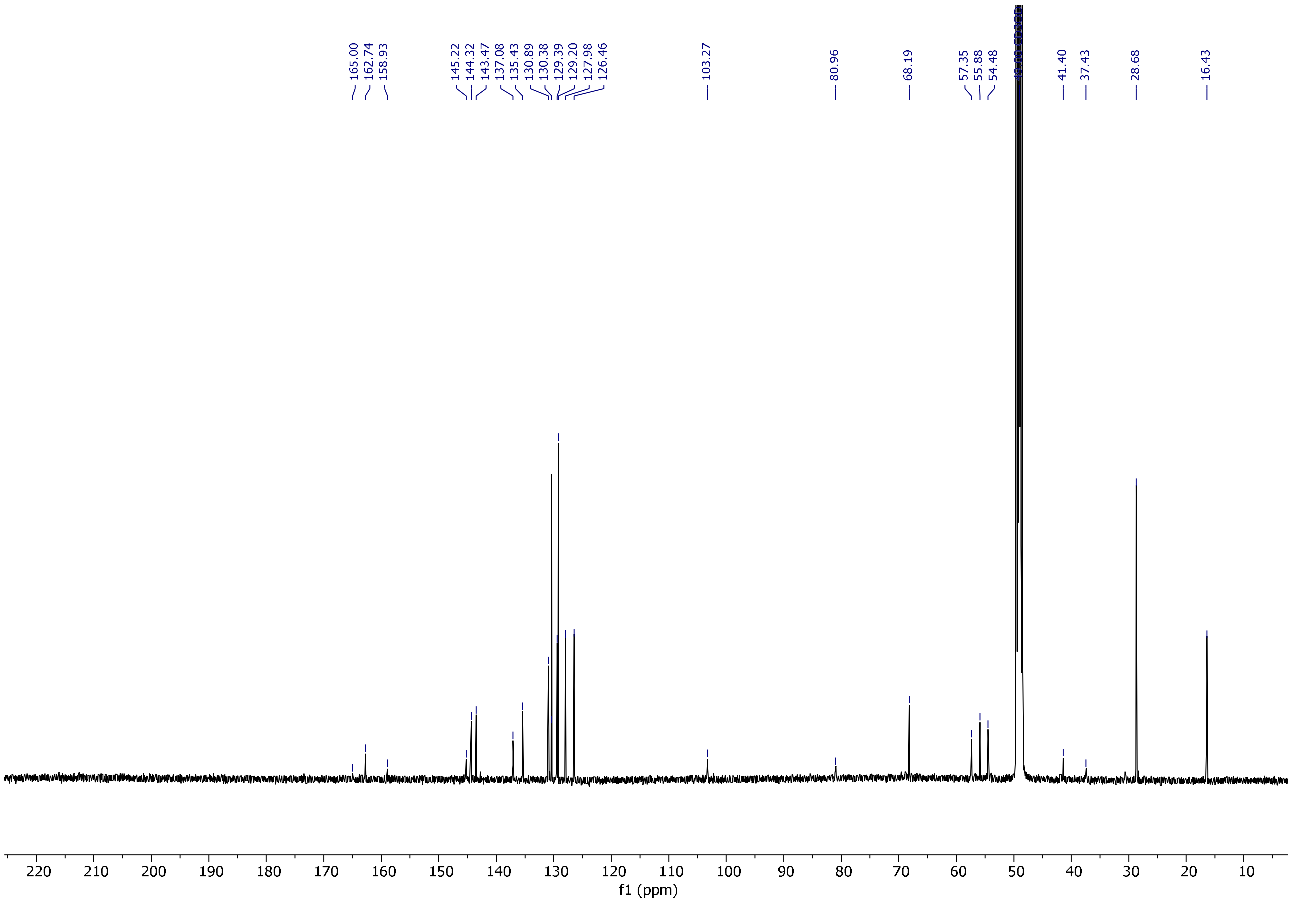

Compound 5

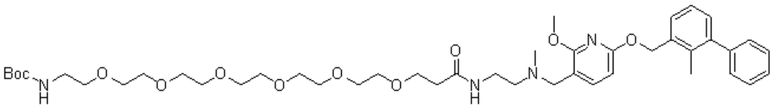

Boc
N
H
O
O
O
O
O
O
O
N
H
N
O
N
O

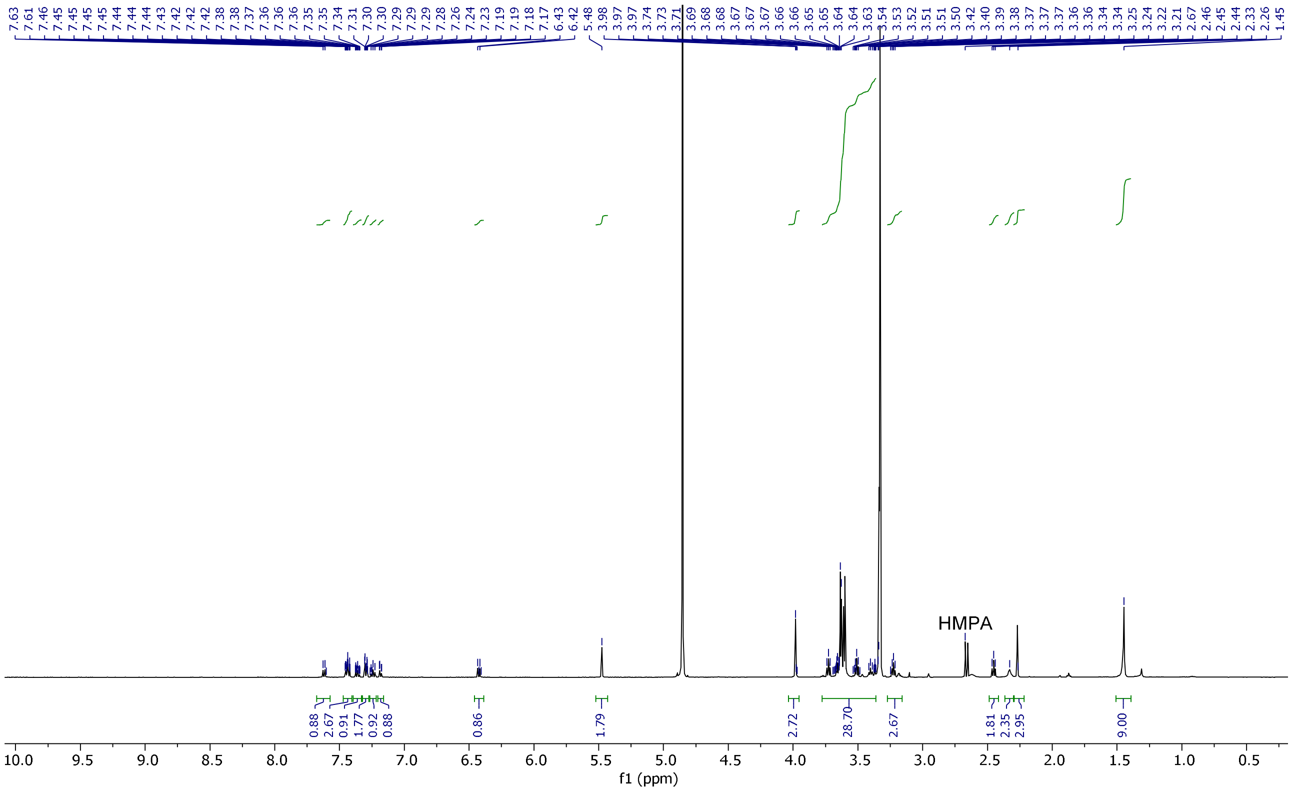

7.63 7.61 7.46 7.45 7.45 7.45 7.44 7.44 7.44 7.43 7.42 7.42 7.38 7.38 7.37 7.36 7.36 7.35 7.35 7.34 7.31 7.30 7.30 7.29 7.29 7.28 7.26 7.24 7.23 7.19 7.19 7.18 7.17 6.43 6.42 5.48 3.98 3.97 3.74 3.73 3.71 3.69 3.68 3.67 3.67 3.66 3.65 3.64 3.64 3.63 3.54 3.53 3.52 3.51 3.51 3.50 3.42 3.40 3.39 3.38 3.37 3.37 3.37 3.36 3.34 3.34 3.25 3.24 3.22 3.21 2.67 2.46 2.45 2.44 2.33 2.26 1.45
HMPA
0.88 2.67 0.91 1.77 0.92 0.88 0.86 1.79 2.72 28.70 2.67 1.81 2.35 2.95 9.00
10.0 9.5 9.0 8.5 8.0 7.5 7.0 6.5 6.0 5.5 5.0 4.5 4.0 3.5 3.0 2.5 2.0 1.5 1.0 0.5
f1 (ppm)

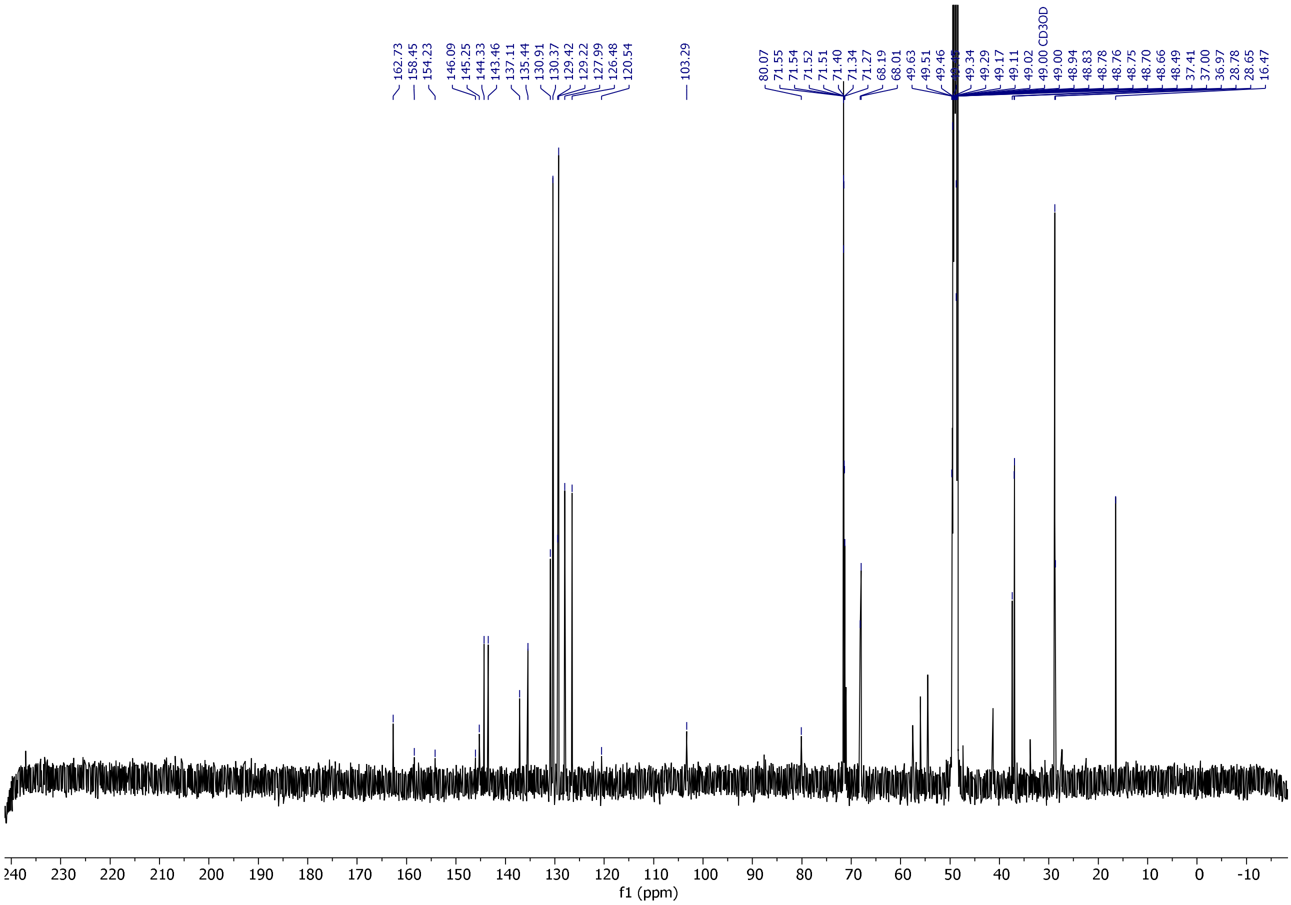

162.73 158.45 154.23 146.09 145.25 144.33 143.46 137.11 135.44 130.91 130.37 129.42 129.22 127.99 126.48 120.54 103.29 80.07 71.55 71.54 71.52 71.51 71.40 71.34 71.27 68.19 68.01 49.63 49.51 49.46 [illegible] 49.34 49.29 49.17 49.11 49.02 49.00 CD3OD 49.00 48.94 48.83 48.78 48.76 48.75 48.70 48.66 48.49 37.41 37.00 36.97 28.78 28.65 16.47
240 230 220 210 200 190 180 170 160 150 140 130 120 110 100 90 80 70 60 50 40 30 20 10 0 -10
f1 (ppm)

Compound 7

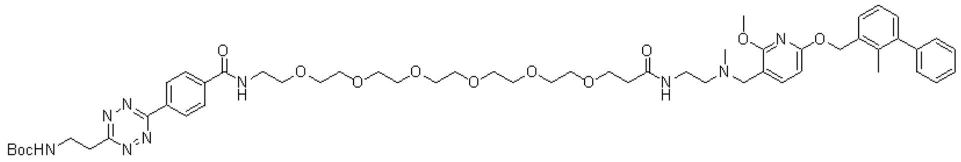
BocHN

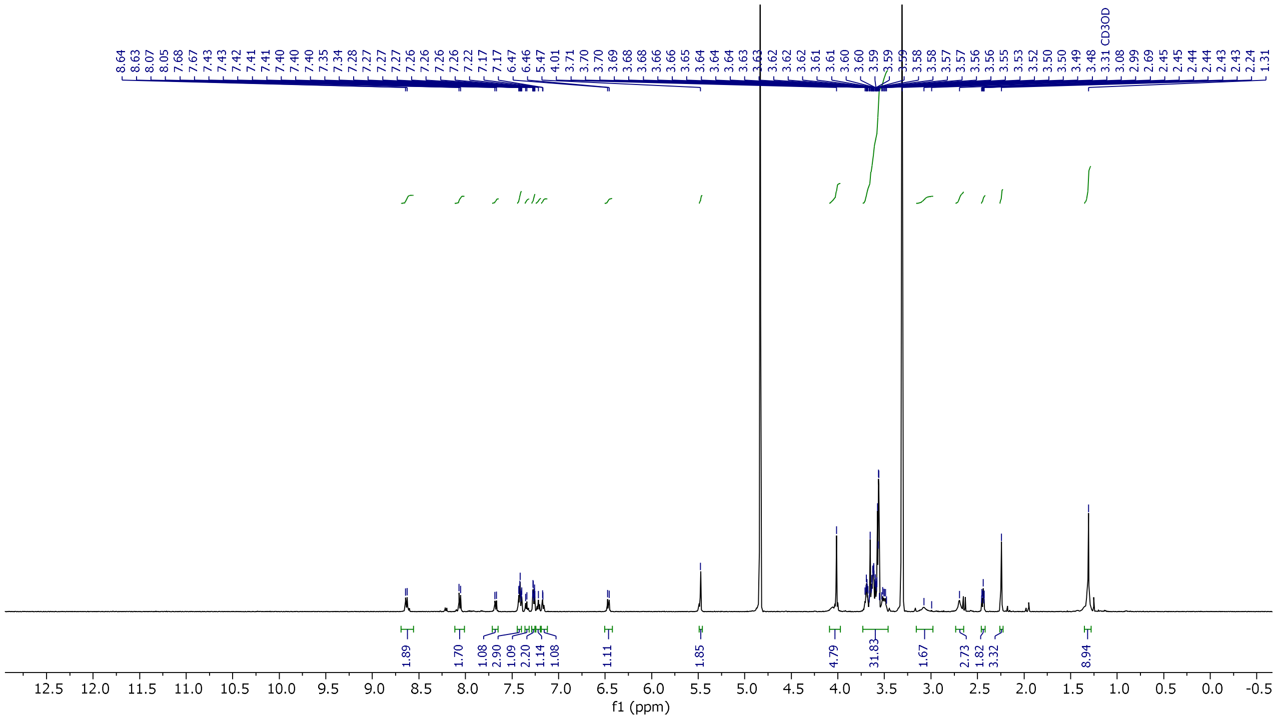
8.64 8.63 8.07 8.05 7.68 7.67 7.43 7.43 7.42 7.41 7.41 7.40 7.40 7.40 7.35 7.34 7.28 7.27 7.27 7.27 7.26 7.26 7.26 7.26 7.22 7.17 7.17 6.47 6.46 5.47 4.01 3.71 3.70 3.69 3.68 3.68 3.66 3.66 3.65 3.64 3.64 3.64 3.63 3.63 3.62 3.62 3.62 3.61 3.61 3.60 3.59 3.59 3.59 3.58 3.58 3.57 3.57 3.56 3.56 3.55 3.53 3.52 3.50 3.50 3.49 3.48 3.31 CD3OD 3.08 2.99 2.69 2.45 2.45 2.44 2.43 2.43 2.24 1.31
1.89 1.70 1.08 2.90 1.09 2.20 1.14 1.08 1.11 1.85 4.79 31.83 1.67 2.73 1.82 3.32 8.94
f1 (ppm)

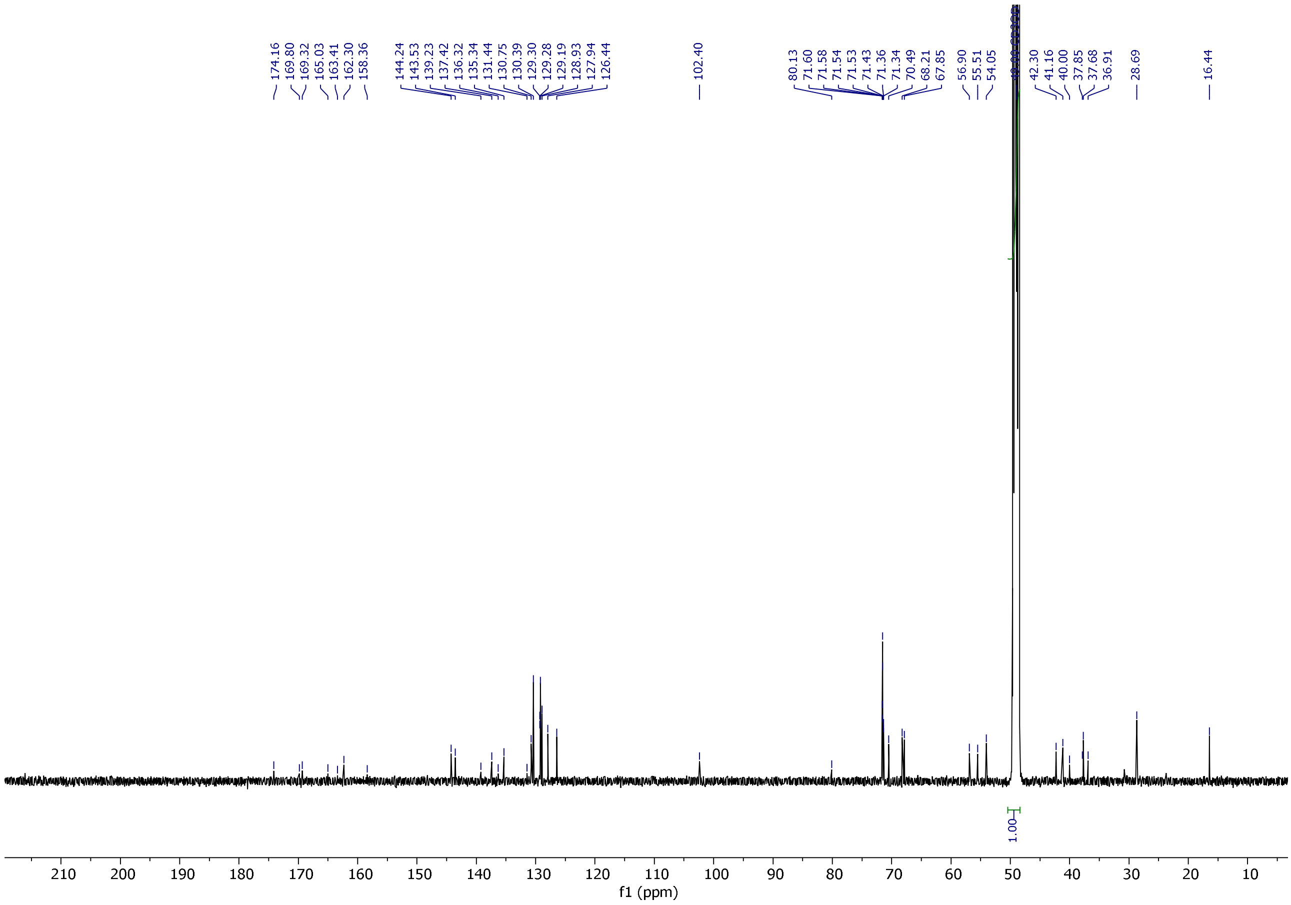
174.16 169.80 169.32 165.03 163.41 162.30 158.36 144.24 143.53 139.23 137.42 136.32 135.34 131.44 130.75 130.39 129.30 129.28 129.19 128.93 127.94 126.44 102.40 80.13 71.60 71.58 71.54 71.53 71.43 71.36 71.34 70.49 68.21 67.85 56.90 55.51 54.05 42.30 41.16 40.00 37.85 37.68 36.91 28.69 16.44
1.00
f1 (ppm)

LTzC

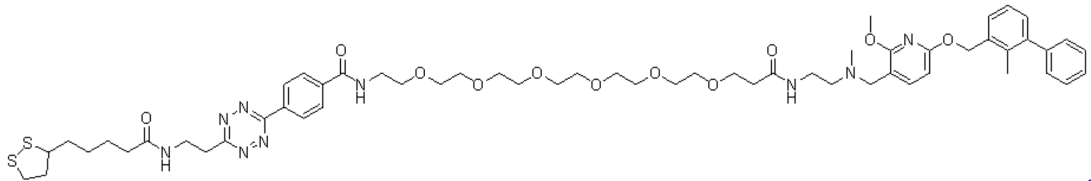

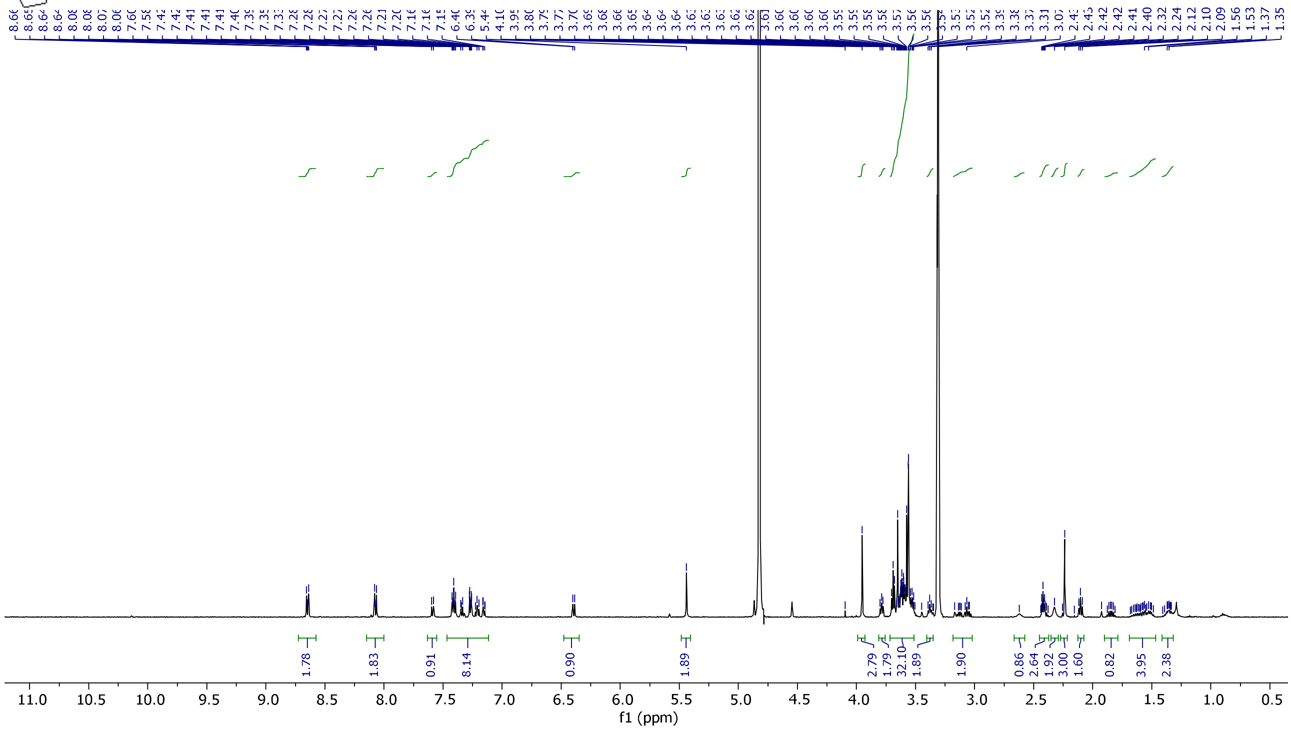

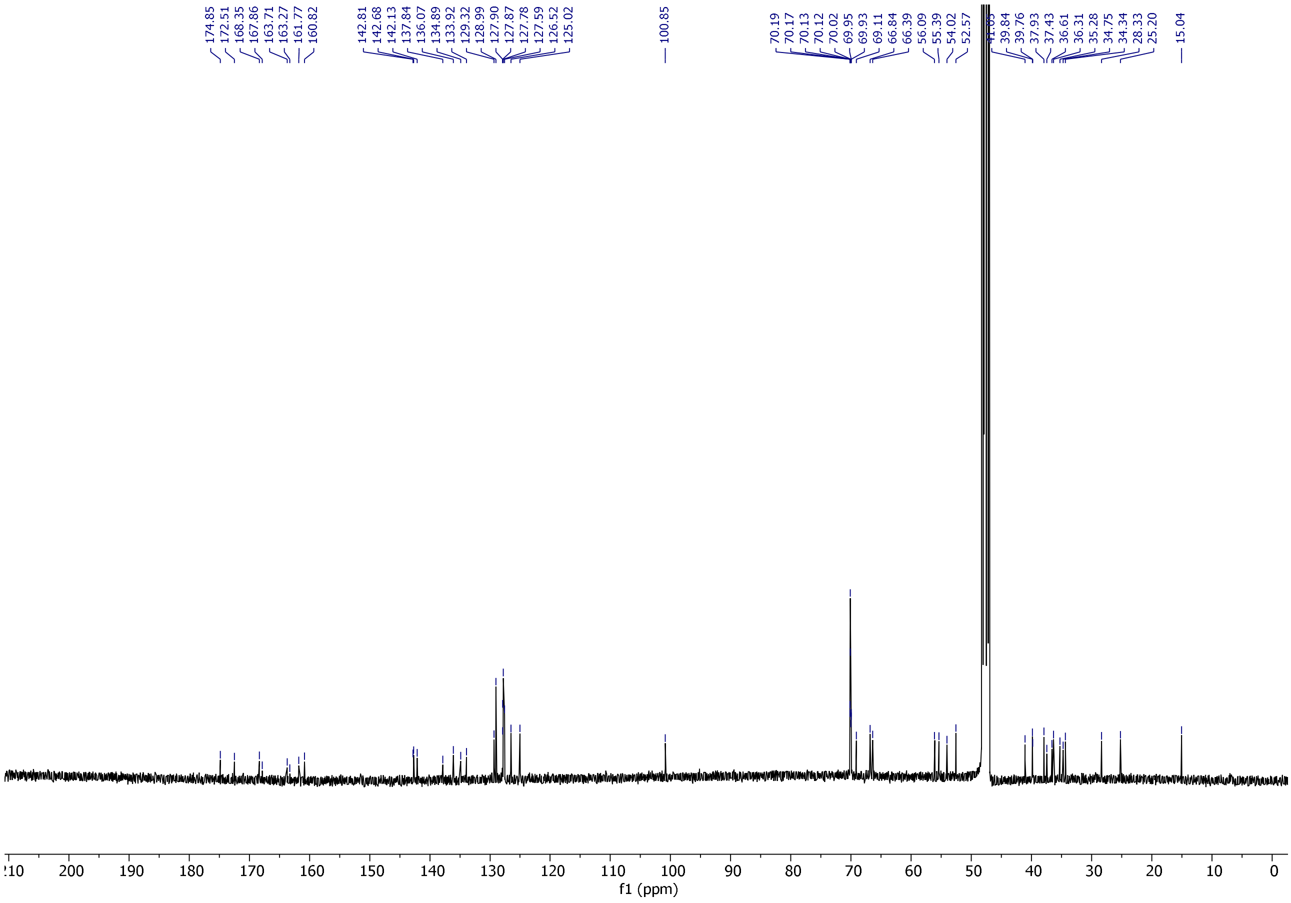

Tetrazine 2

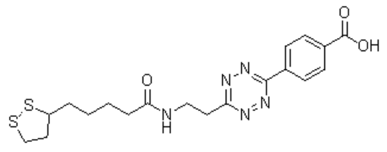
O
OH
N
N
N
N
O
S
S
N
H

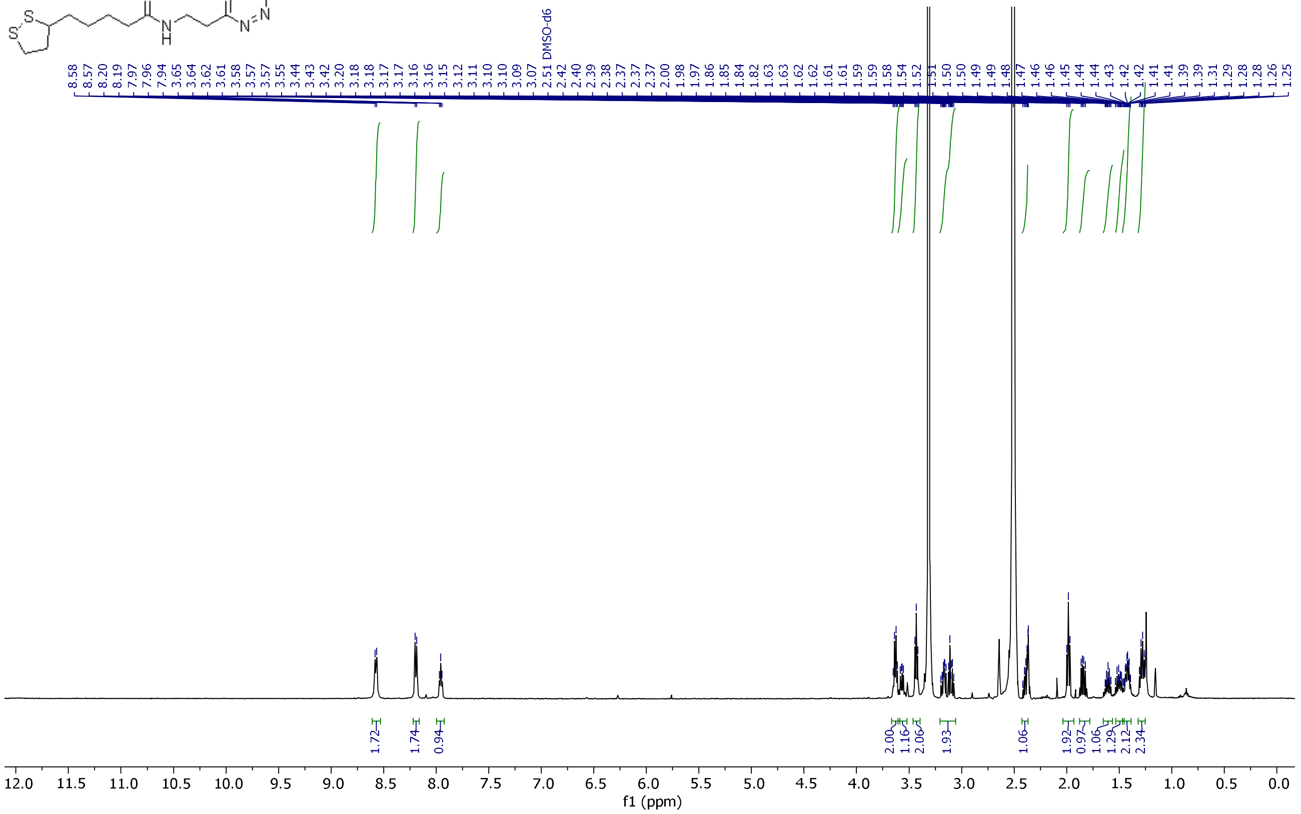
8.58 8.57 8.20 8.19 7.97 7.96 7.94 3.65 3.64 3.62 3.61 3.58 3.57 3.57 3.55 3.44 3.43 3.42 3.20 3.18 3.18 3.17 3.17 3.16 3.16 3.15 3.12 3.11 3.10 3.10 3.09 3.07 2.51 DMSO-d6 2.42 2.40 2.39 2.38 2.37 2.37 2.37 2.00 1.98 1.97 1.86 1.85 1.84 1.82 1.63 1.63 1.62 1.61 1.61 1.59 1.59 1.58 1.54 1.52 1.51 1.50 1.50 1.49 1.49 1.48 1.47 1.46 1.46 1.45 1.44 1.44 1.43 1.42 1.42 1.41 1.39 1.31 1.29 1.28 1.26 1.25
1.72 1.74 0.94 2.00 1.16 2.06 1.93 1.06 1.92 0.97 1.06 1.29 2.12 2.34
12.0 11.5 11.0 10.5 10.0 9.5 9.0 8.5 8.0 7.5 7.0 6.5 6.0 5.5 5.0 4.5 4.0 3.5 3.0 2.5 2.0 1.5 1.0 0.5 0.0
f1 (ppm)

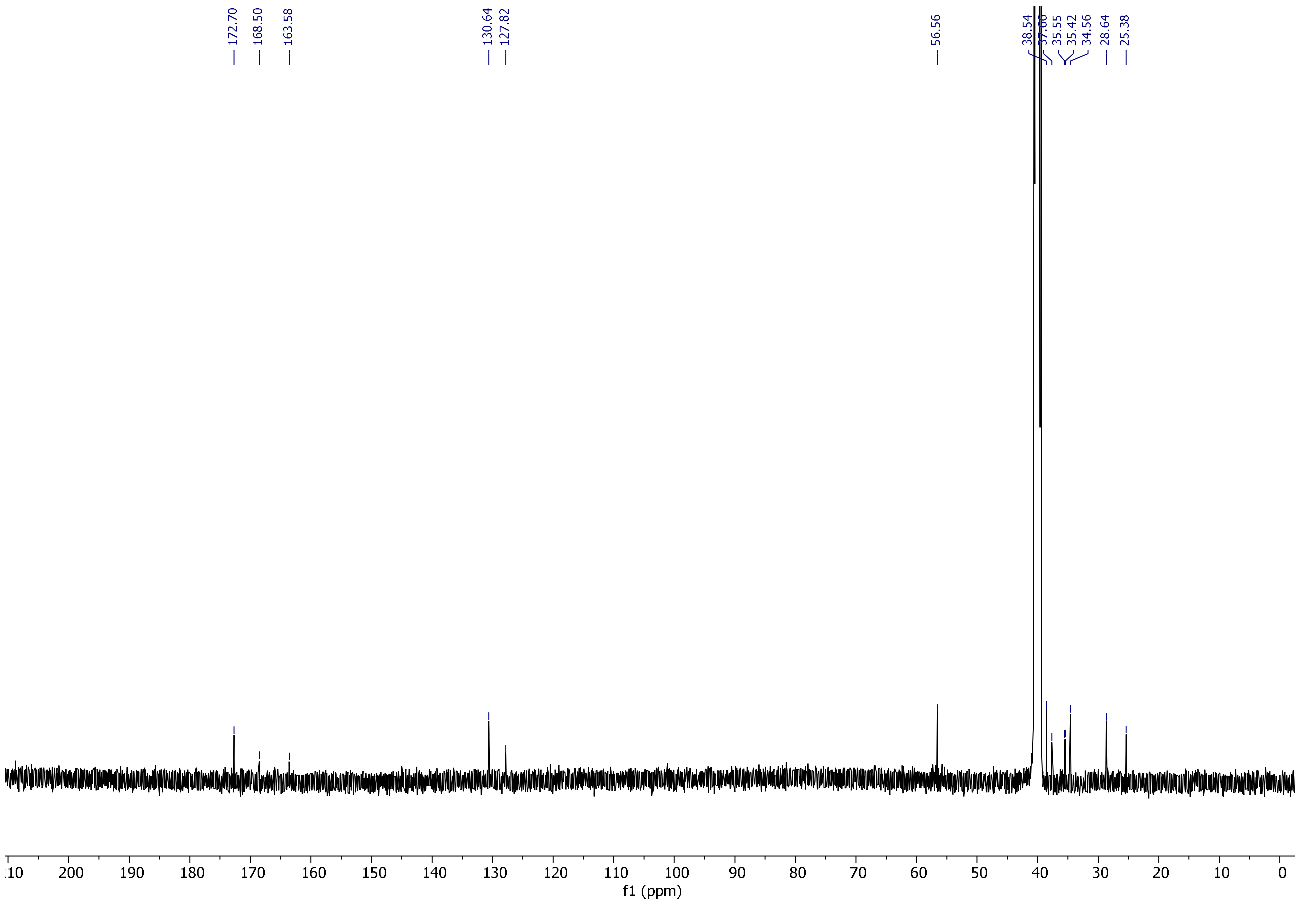
172.70 168.50 163.58 130.64 127.82 56.56 38.54 37.00 35.55 35.42 34.56 28.64 25.38
210 200 190 180 170 160 150 140 130 120 110 100 90 80 70 60 50 40 30 20 10 0
f1 (ppm)

Non-targeting LTzC

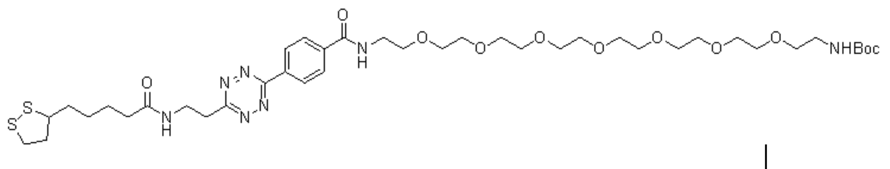

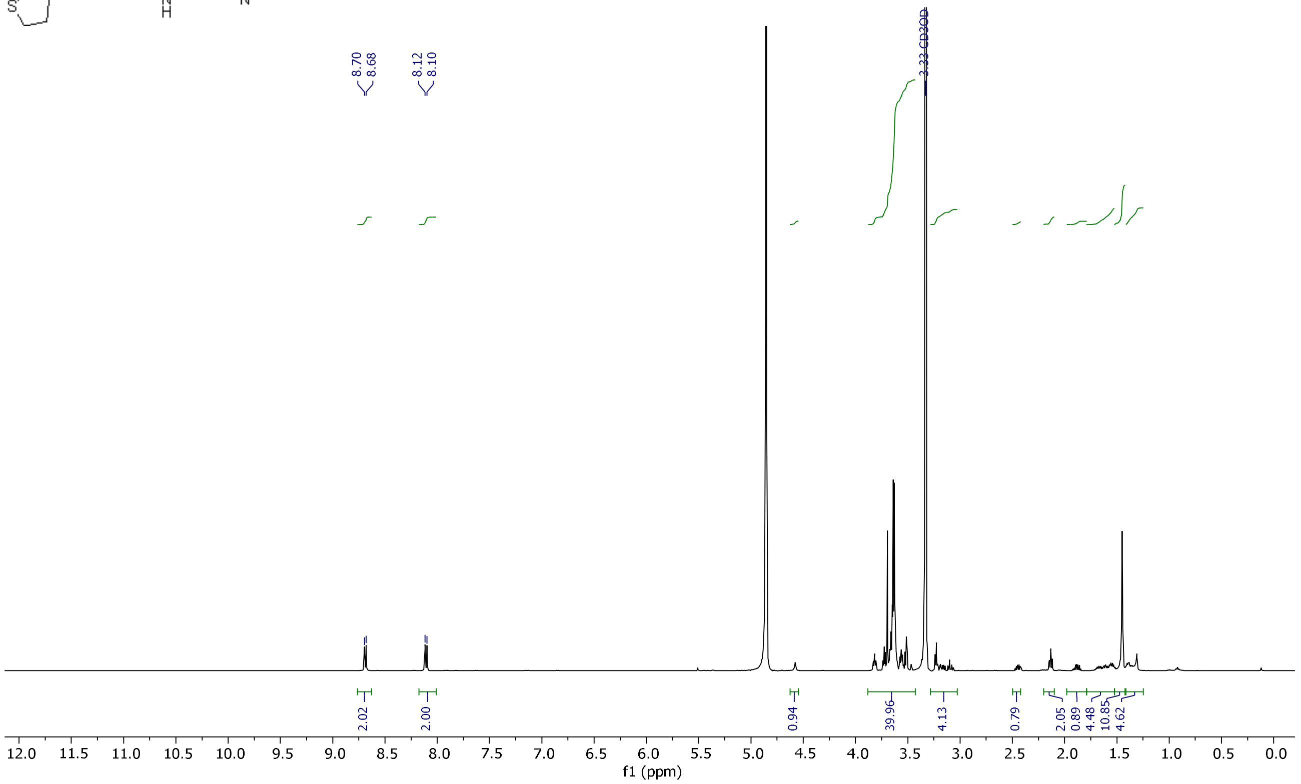

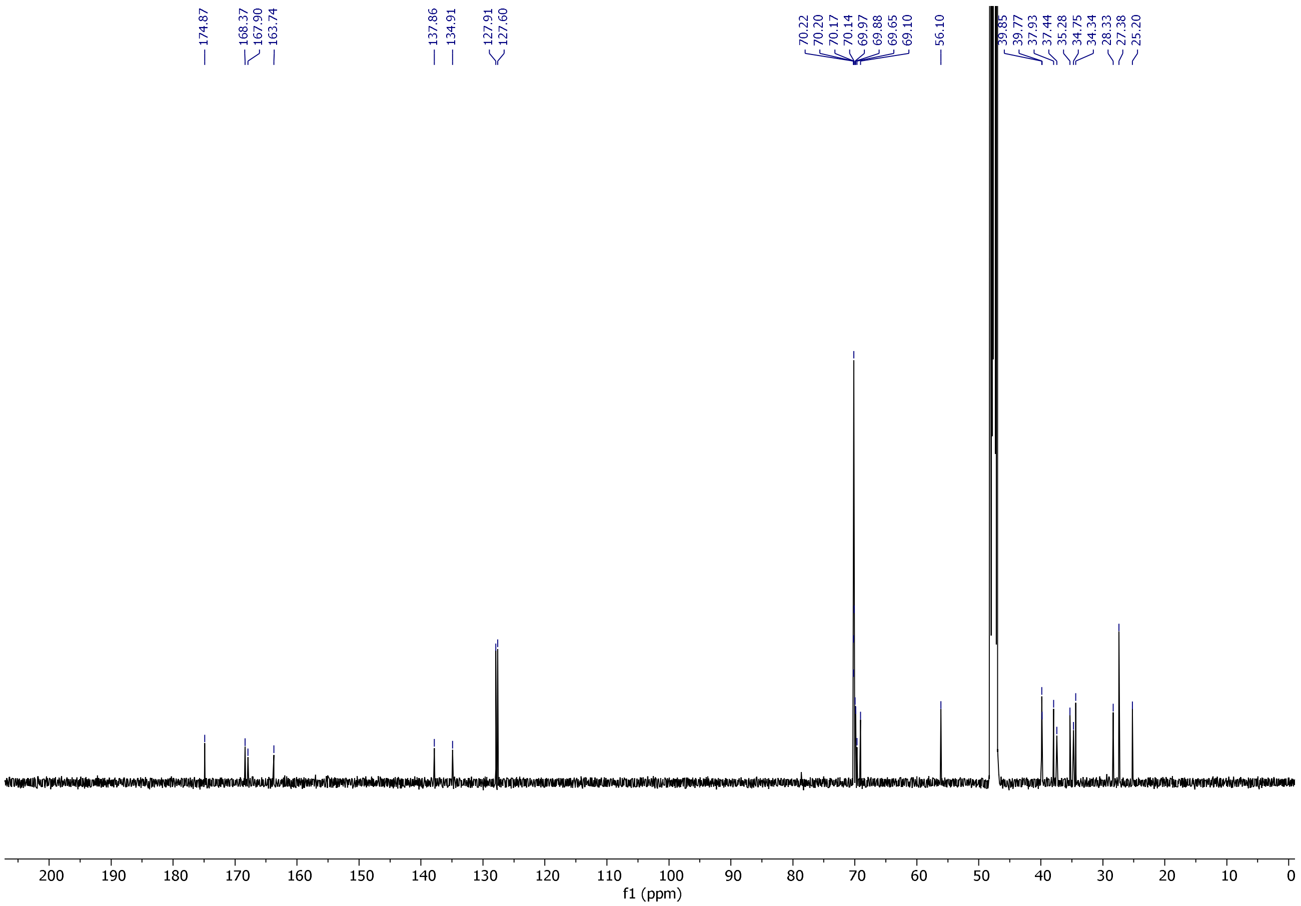

Compound 8

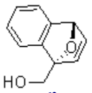

HO
O

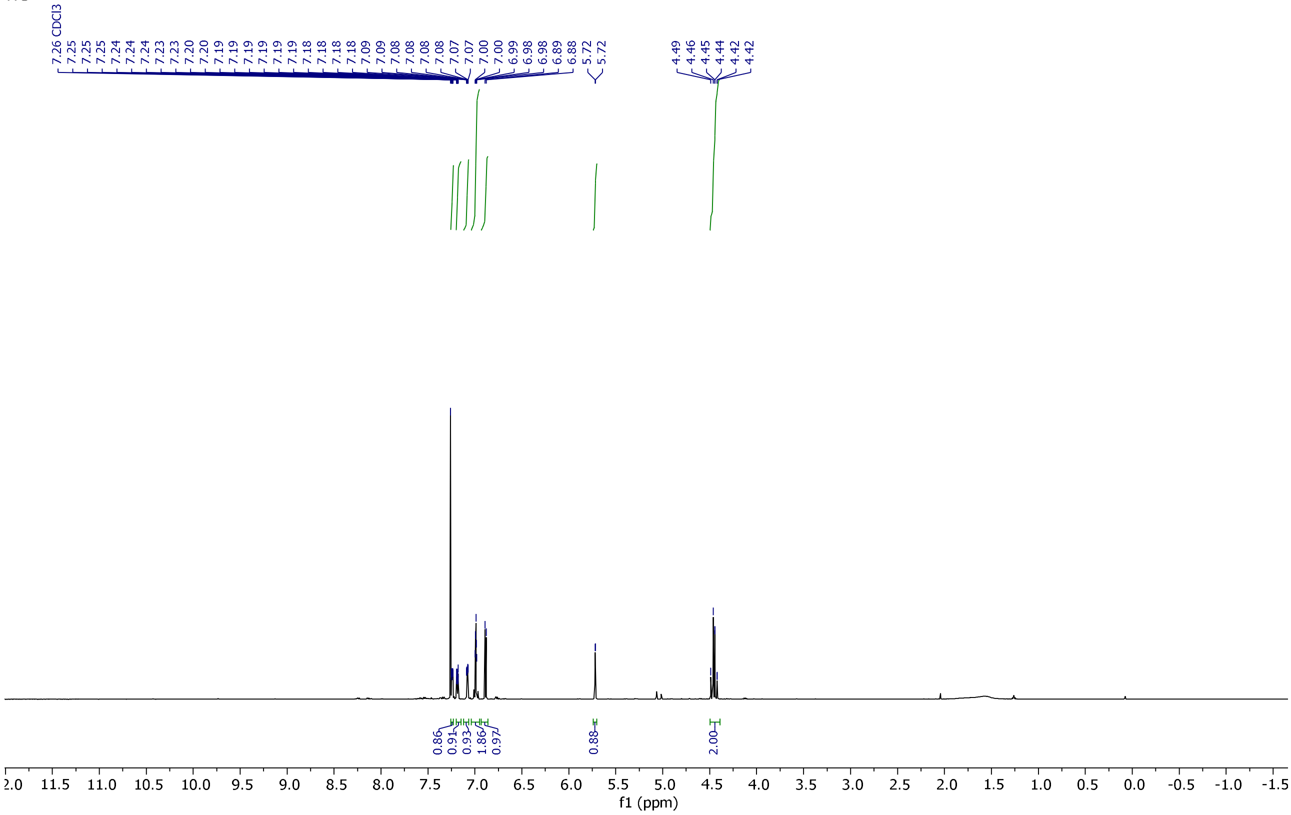

7.26 CDCl3
7.25
7.25
7.25
7.24
7.24
7.24
7.23
7.23
7.20
7.20
7.19
7.19
7.19
7.19
7.19
7.19
7.18
7.18
7.18
7.18
7.09
7.09
7.08
7.08
7.08
7.08
7.07
7.07
7.00
7.00
6.99
6.98
6.98
6.89
6.88
5.72
5.72
4.49
4.46
4.45
4.44
4.42
4.42
0.86
0.91
0.93
1.86
0.97
0.88
2.00
f1 (ppm)

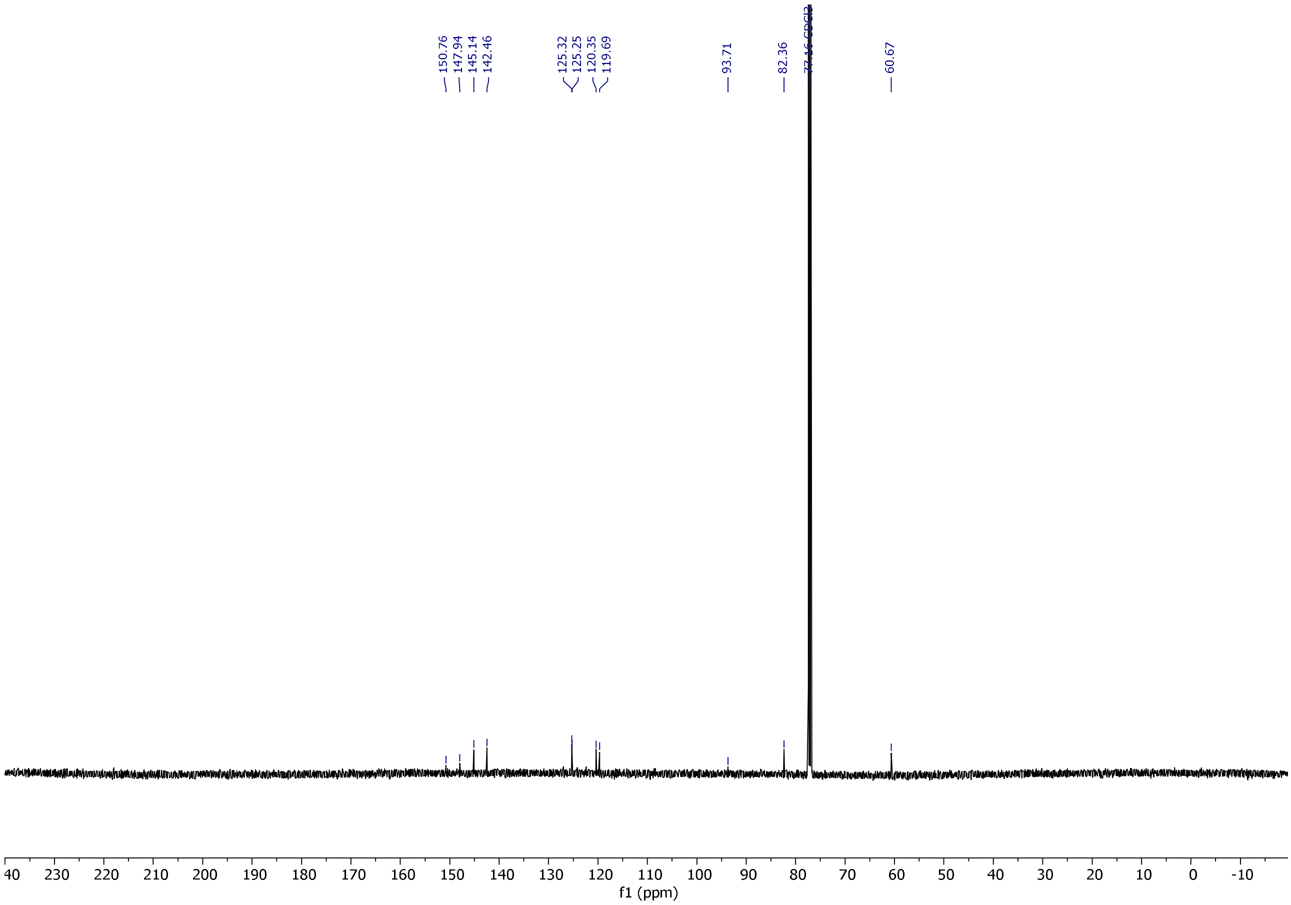

150.76
147.94
145.14
142.46
125.32
125.25
120.35
119.69
93.71
82.36
77.16 CDCl3
60.67
f1 (ppm)

Compound 9

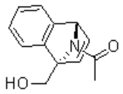

N
O
HO

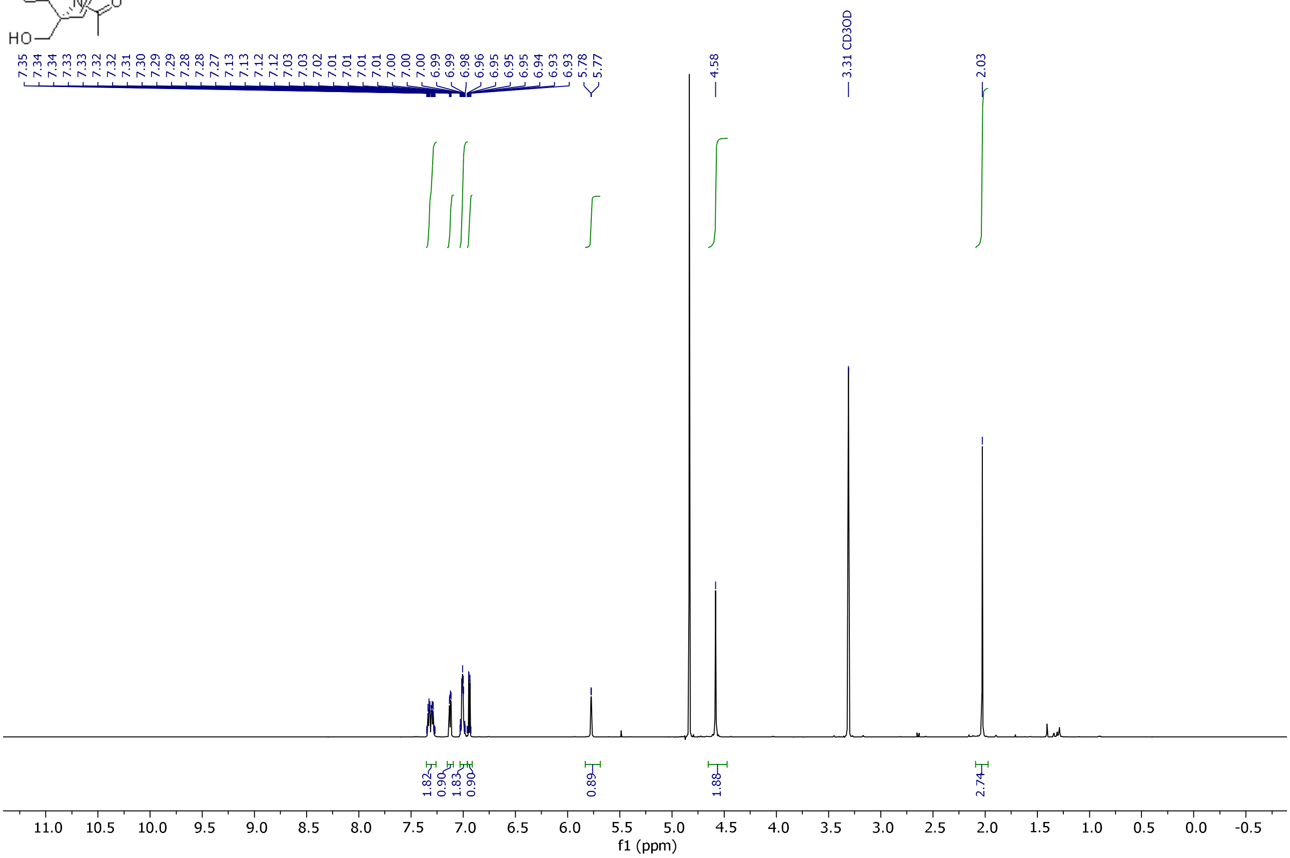

7.35
7.34
7.34
7.33
7.33
7.32
7.31
7.30
7.29
7.29
7.28
7.28
7.27
7.13
7.13
7.12
7.12
7.03
7.02
7.01
7.01
7.01
7.00
7.00
6.99
6.99
6.98
6.96
6.95
6.95
6.95
6.94
6.93
5.78
5.77
4.58
3.31 CD3OD
2.03
1.82
0.90
1.83
0.90
0.89
1.88
2.74
11.0 10.5 10.0 9.5 9.0 8.5 8.0 7.5 7.0 6.5 6.0 5.5 5.0 4.5 4.0 3.5 3.0 2.5 2.0 1.5 1.0 0.5 0.0 -0.5
f1 (ppm)

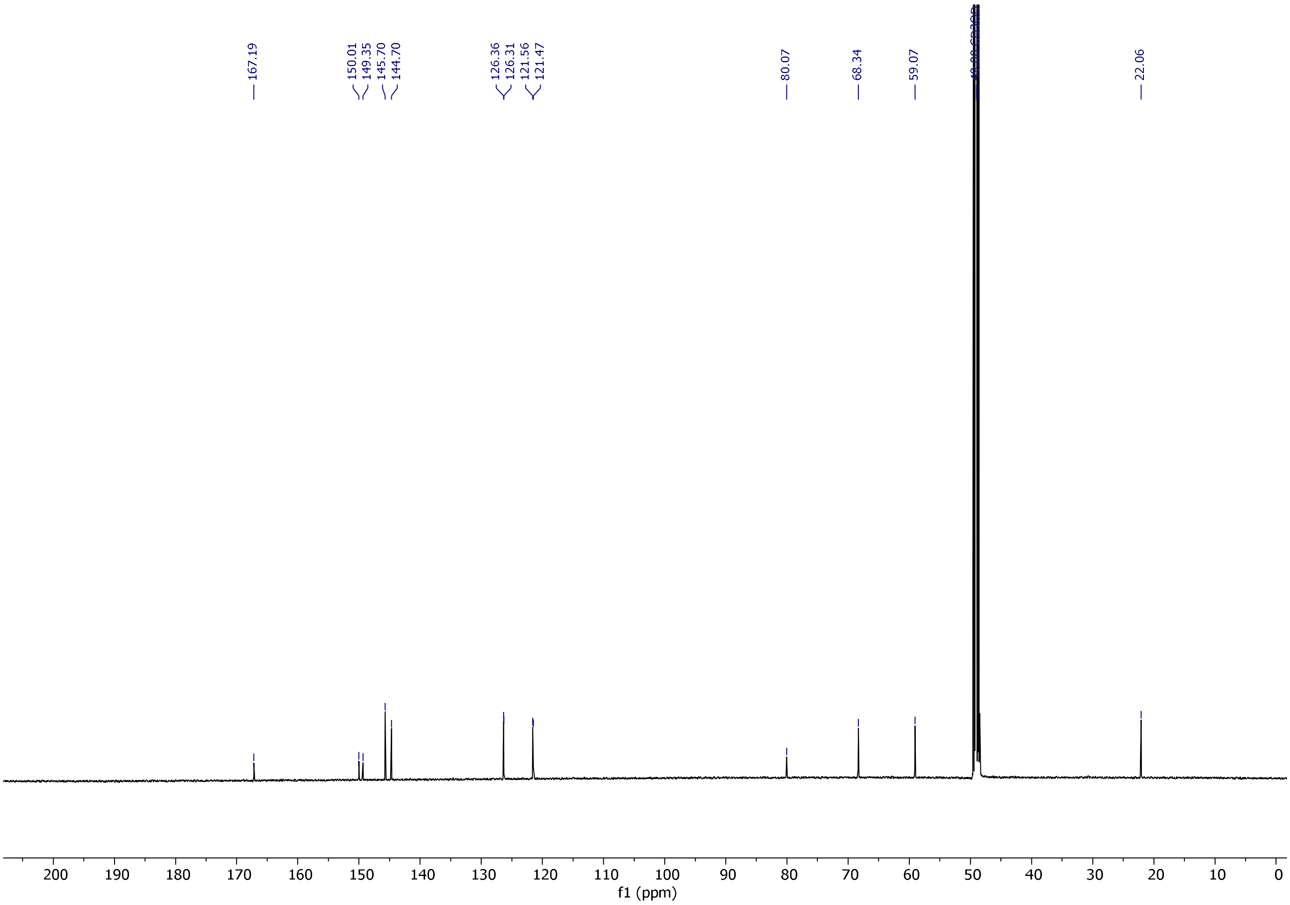

167.19
150.01
149.35
145.70
144.70
126.36
126.31
121.56
121.47
80.07
68.34
59.07
[illegible] CD3OD
22.06
200 190 180 170 160 150 140 130 120 110 100 90 80 70 60 50 40 30 20 10 0
f1 (ppm)

N-butyl Lucifer (Compound 10)

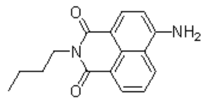

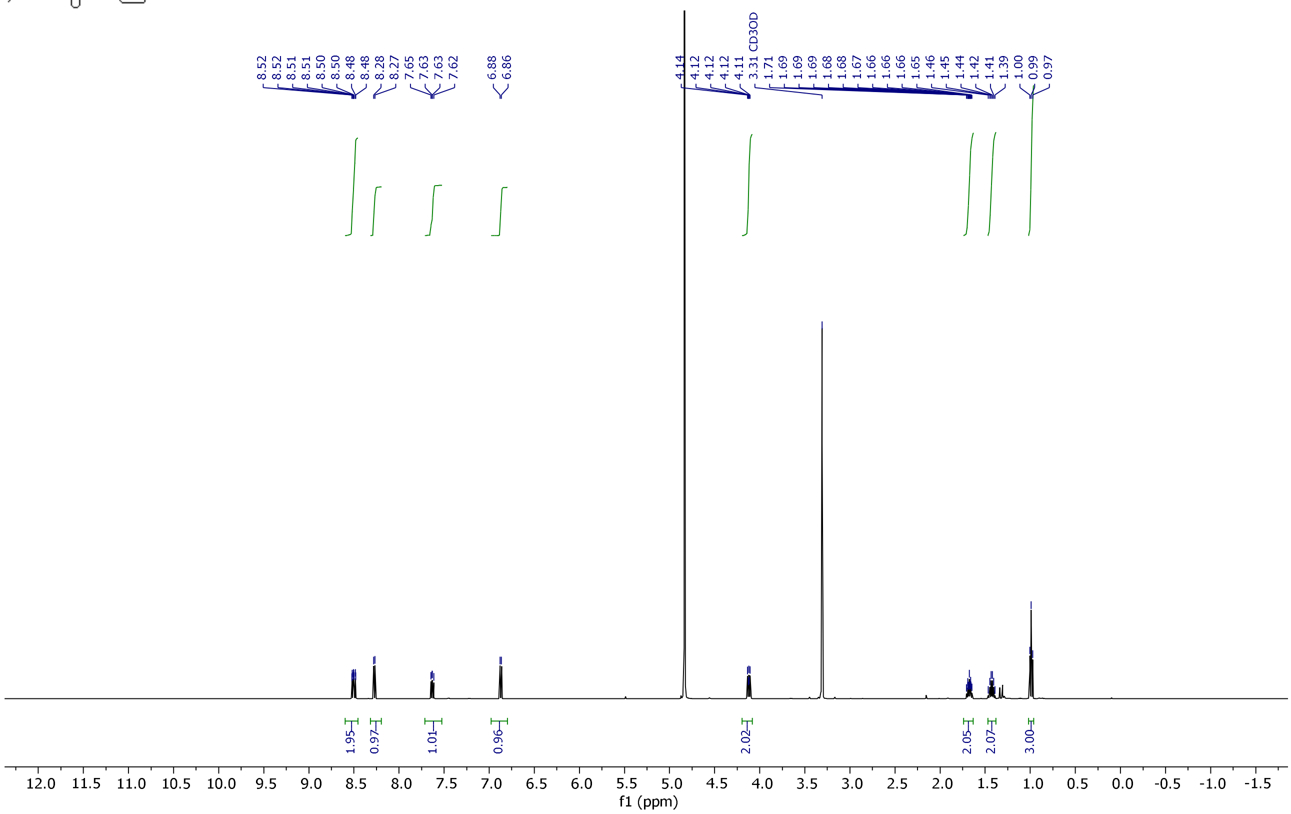

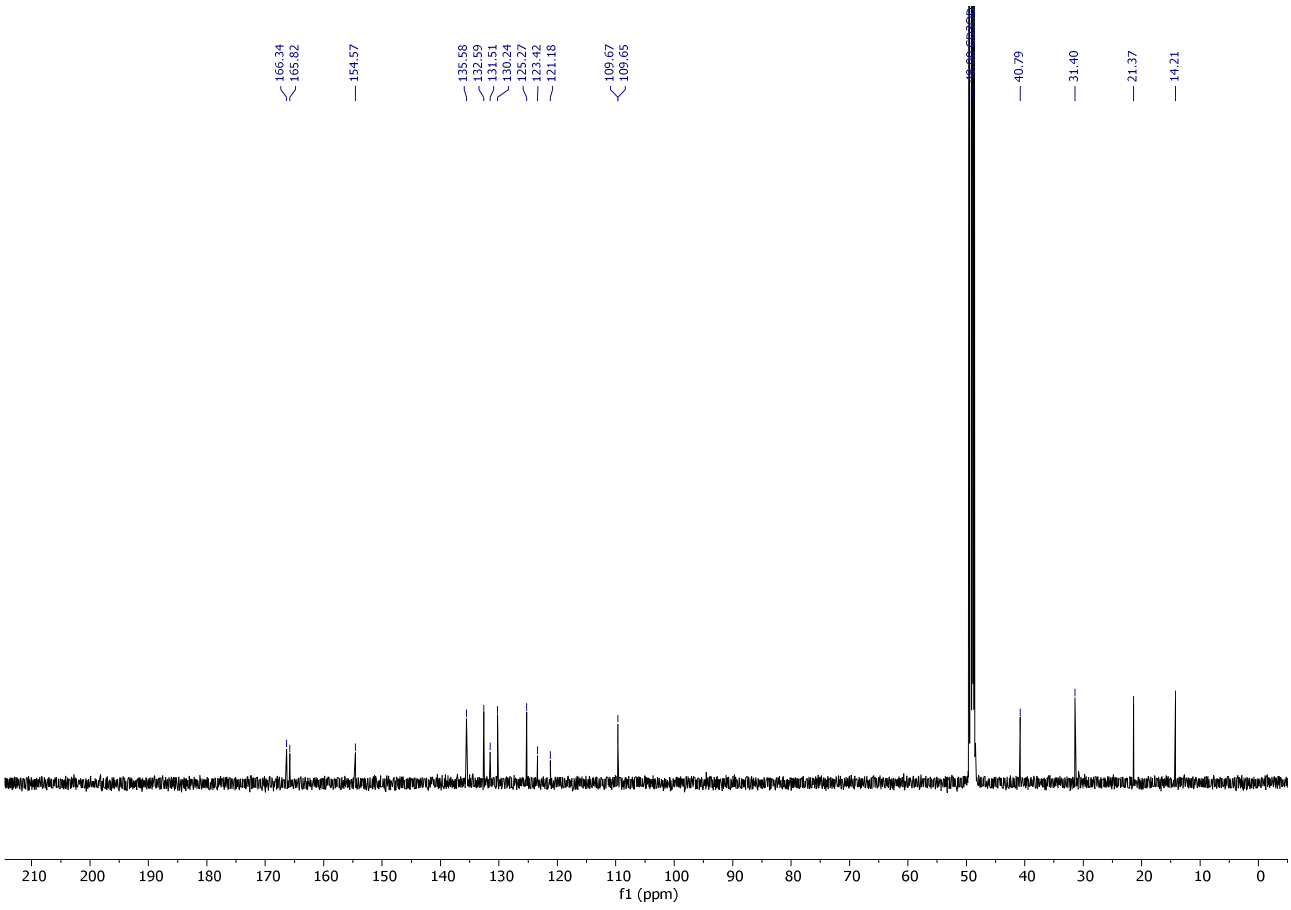

Pro-fluorophore 1

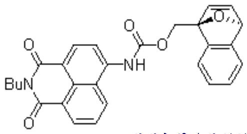

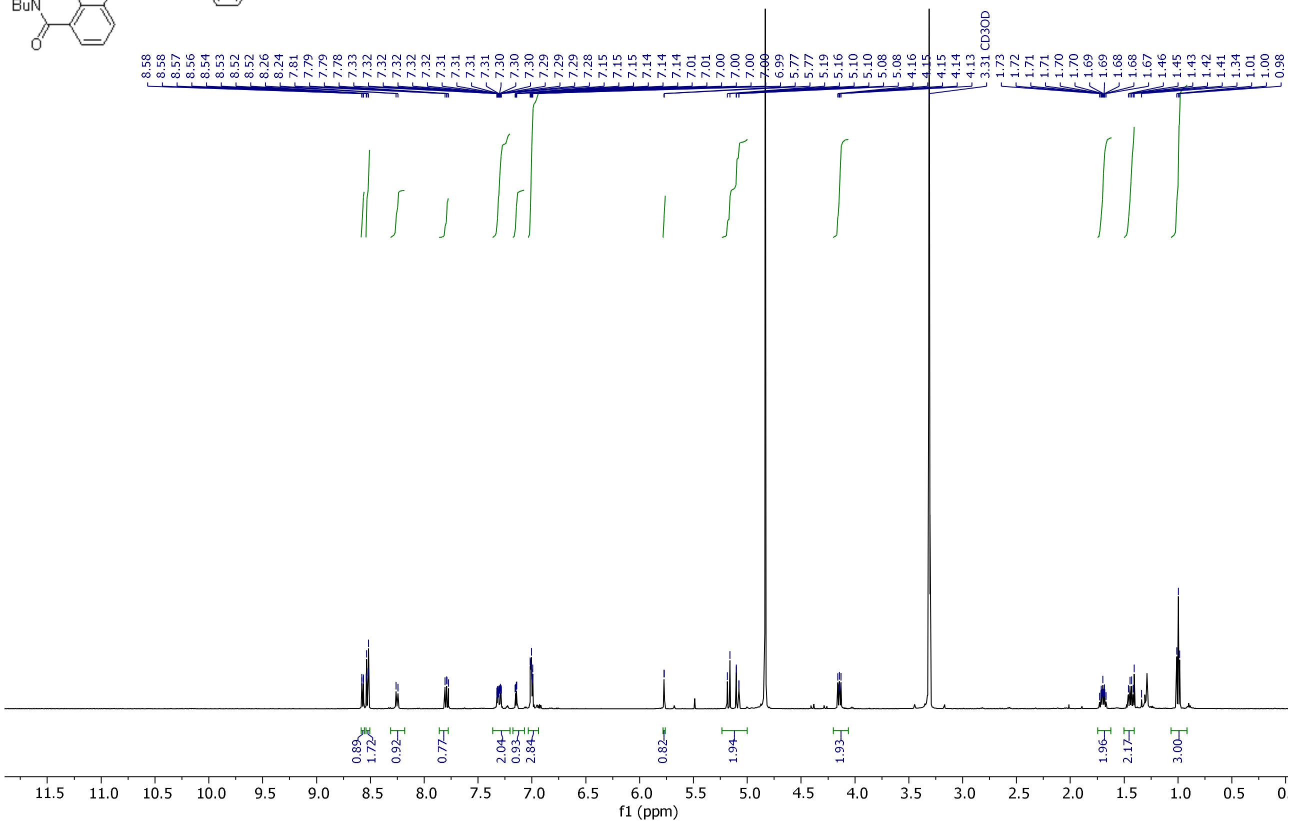

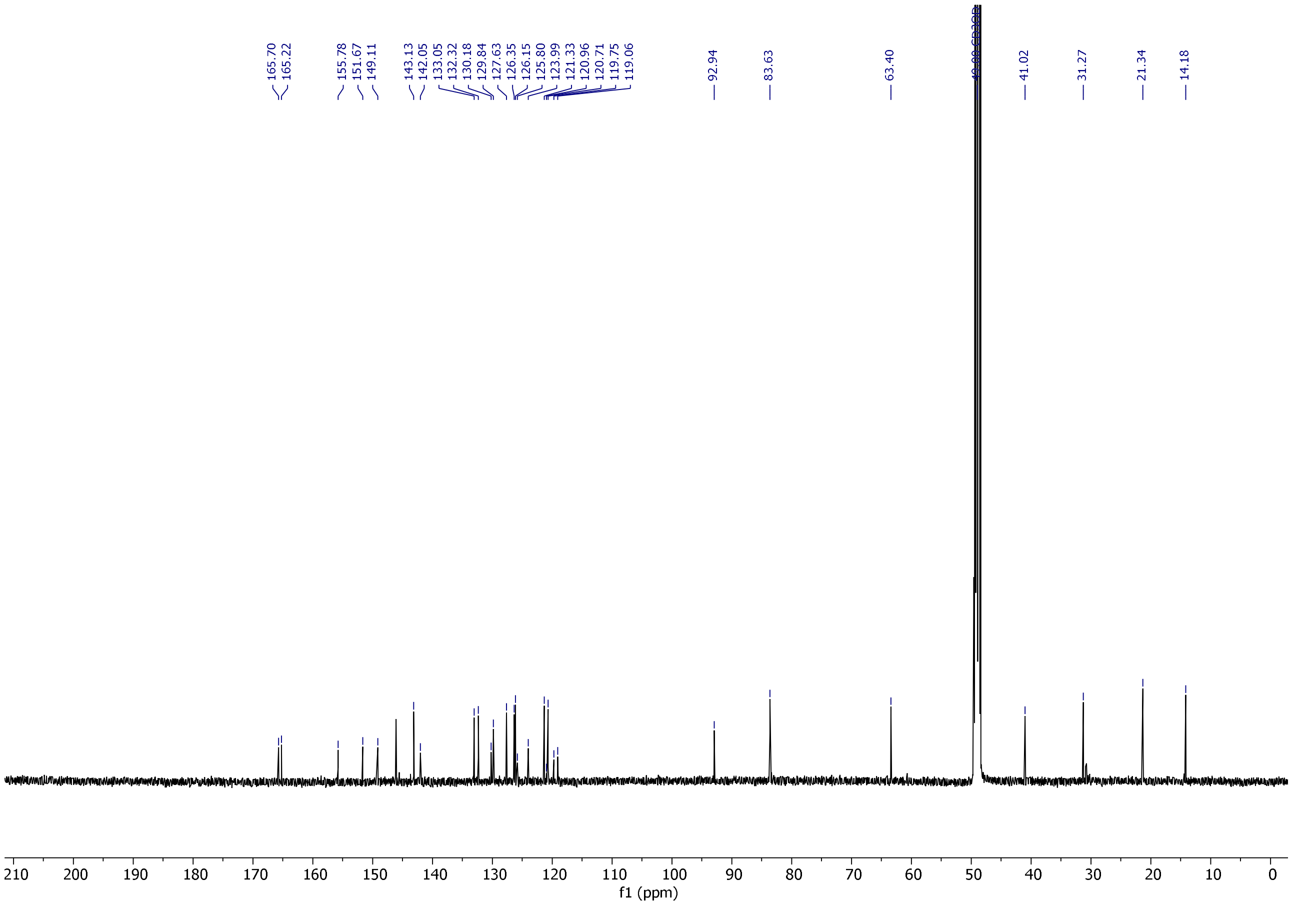

Pro-fluorophore 2

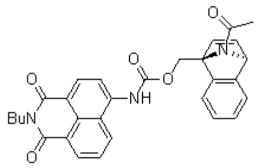

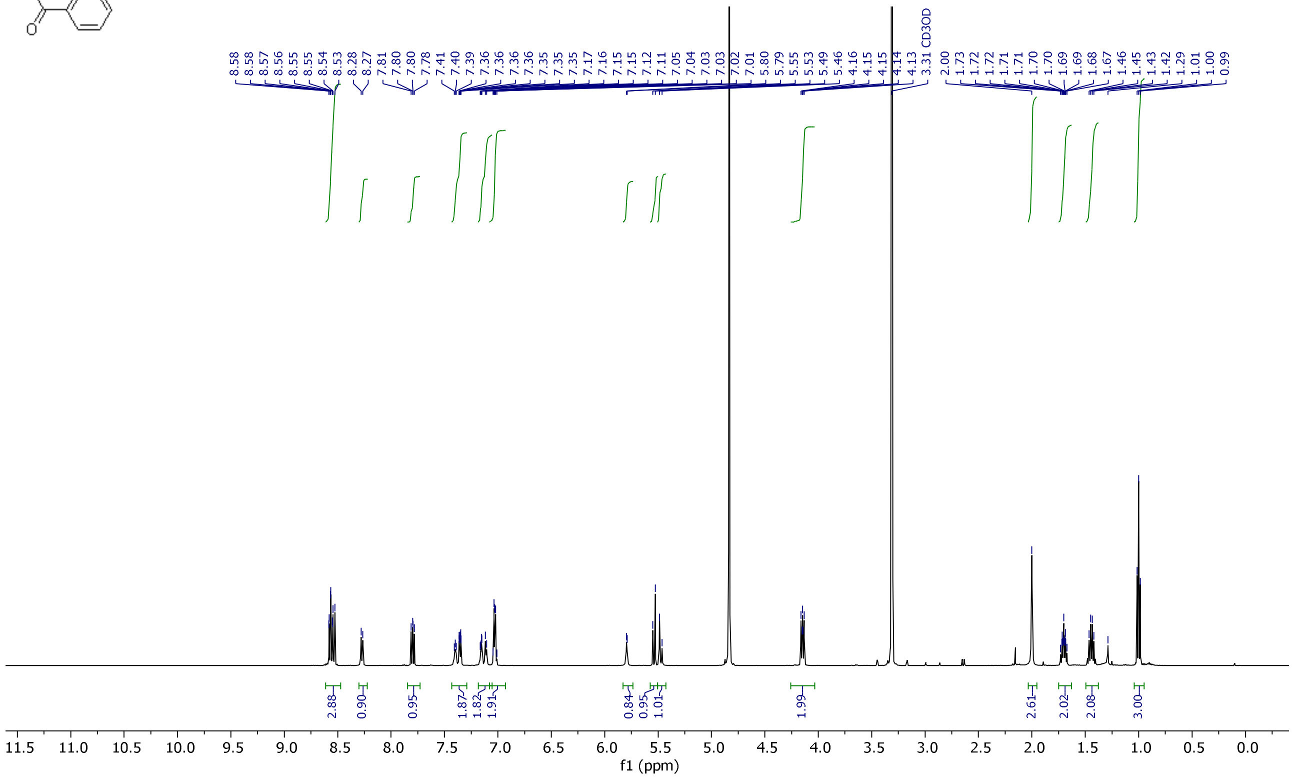

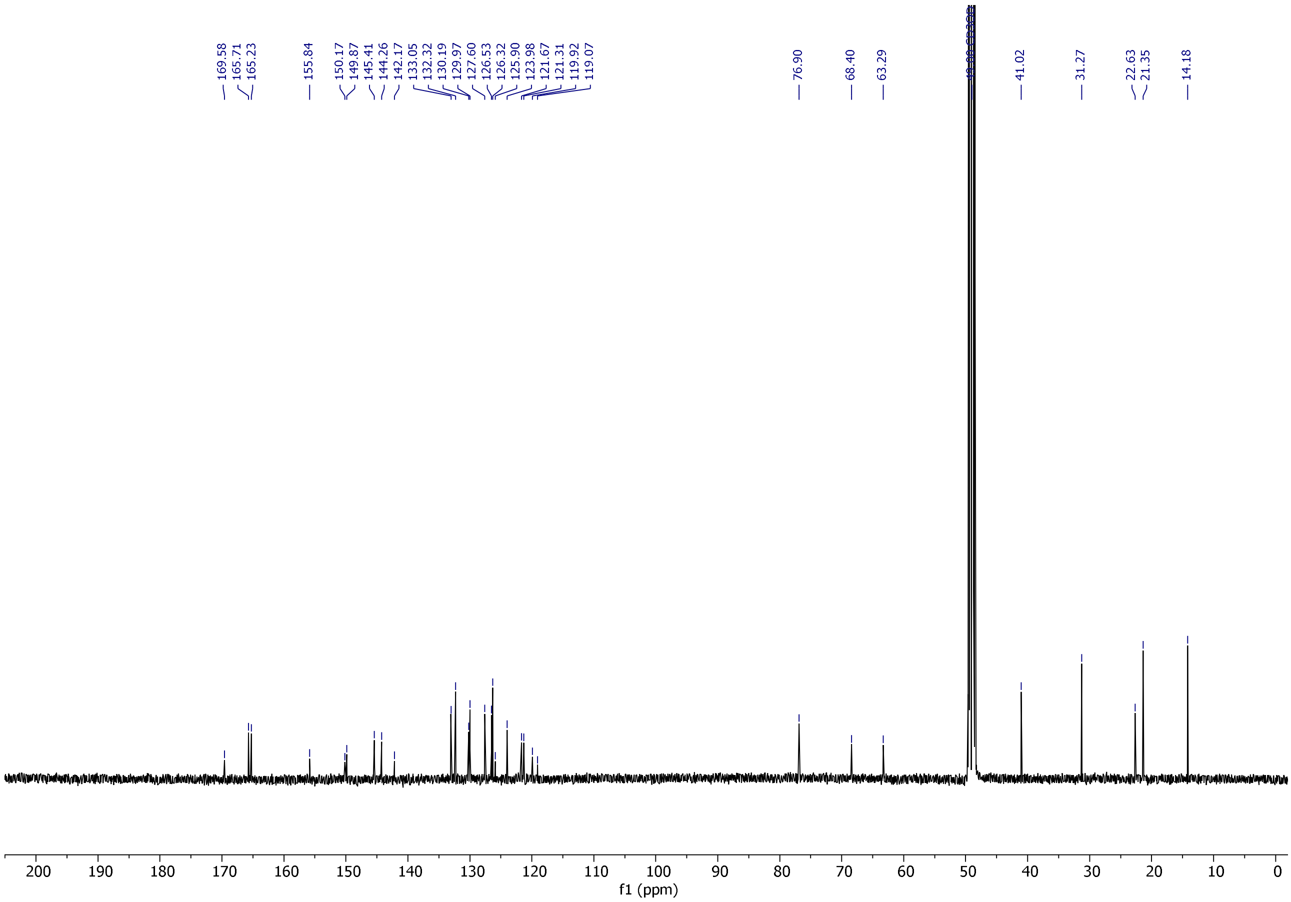

Compound 11

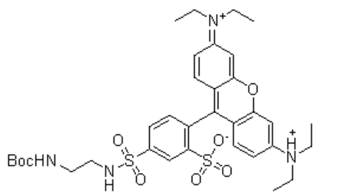
BocHN

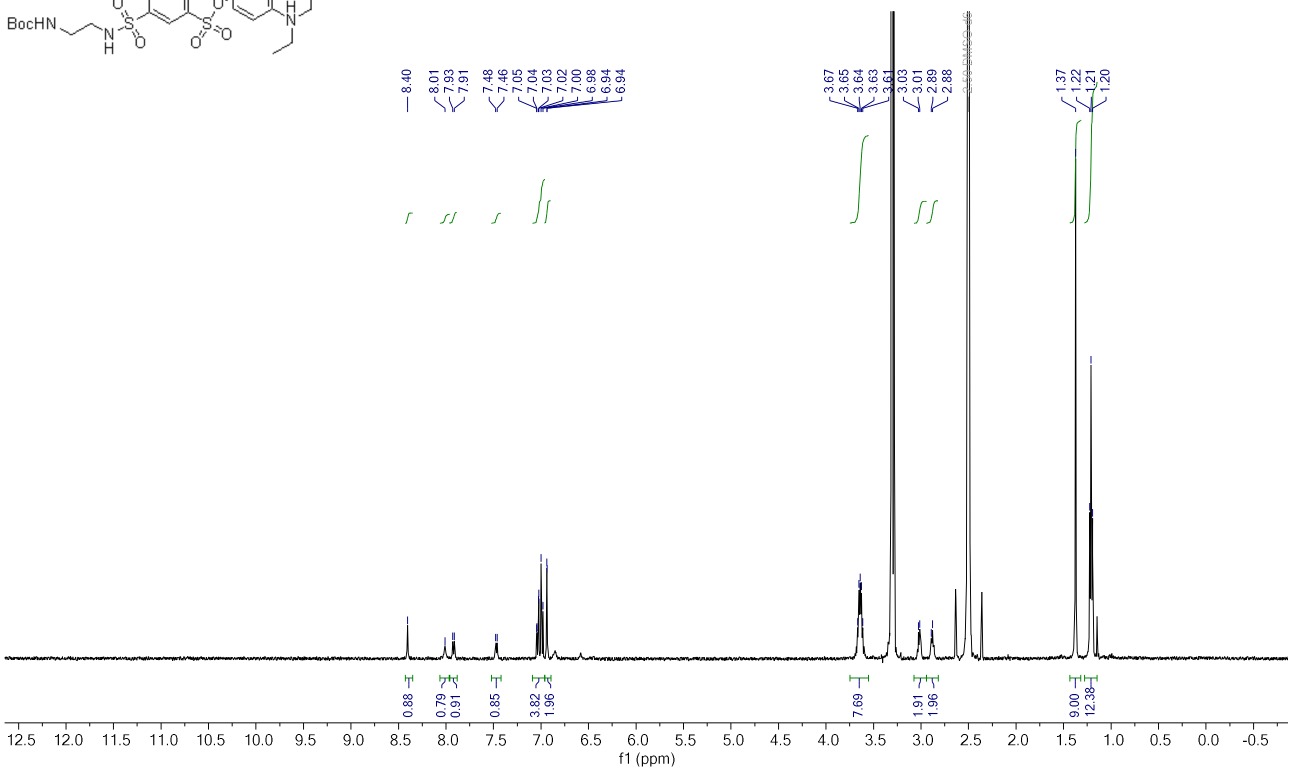
8.40
8.01
7.93
7.91
7.48
7.46
7.05
7.04
7.03
7.02
7.00
6.98
6.94
6.94
3.67
3.65
3.64
3.63
3.61
3.03
3.01
2.89
2.88
1.37
1.22
1.21
1.20
0.88
0.79
0.91
0.85
3.82
1.96
7.69
1.91
1.96
9.00
12.38
f1 (ppm)

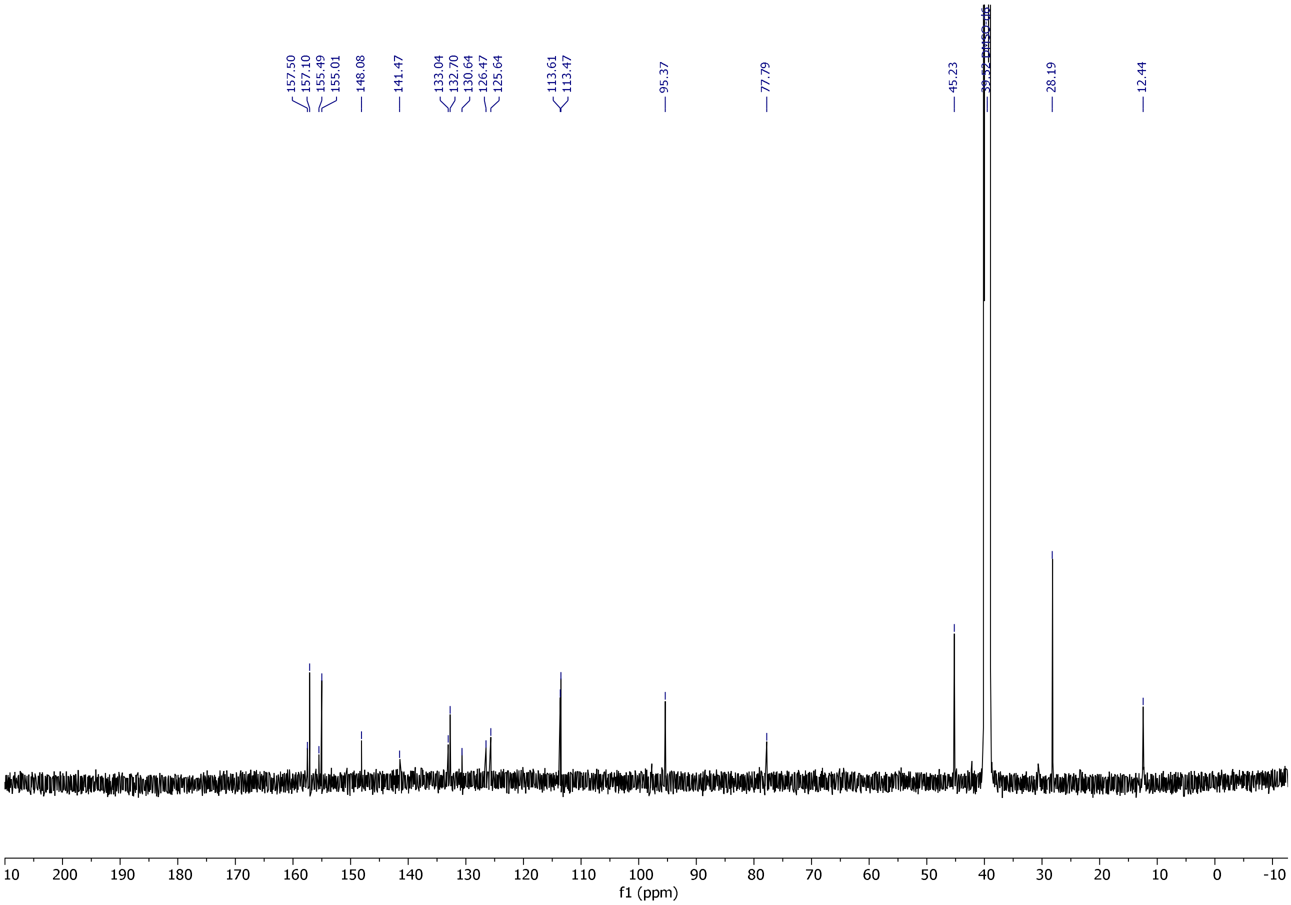
157.50
157.10
155.49
155.01
148.08
141.47
133.04
132.70
130.64
126.47
125.64
113.61
113.47
95.37
77.79
45.23
28.19
12.44
f1 (ppm)

SRB probe

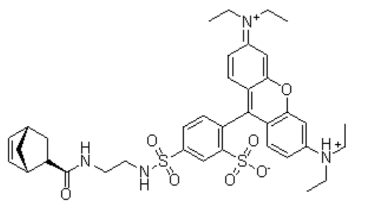

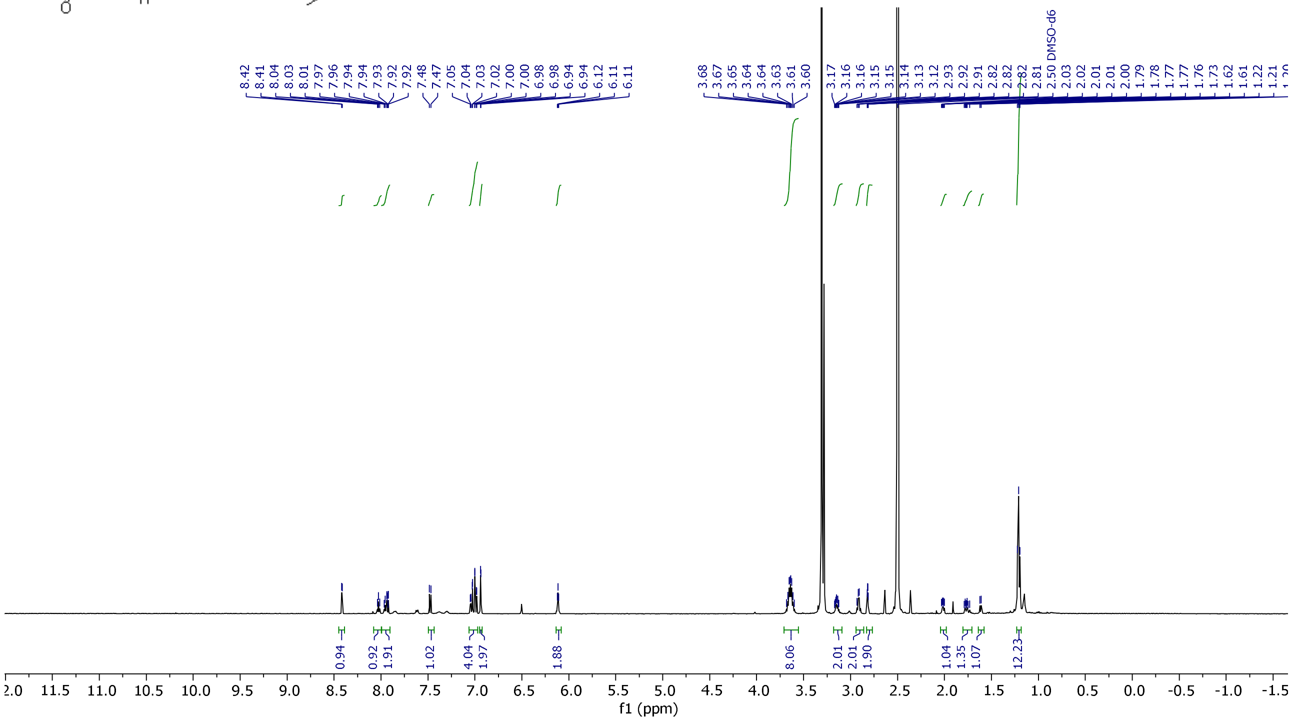

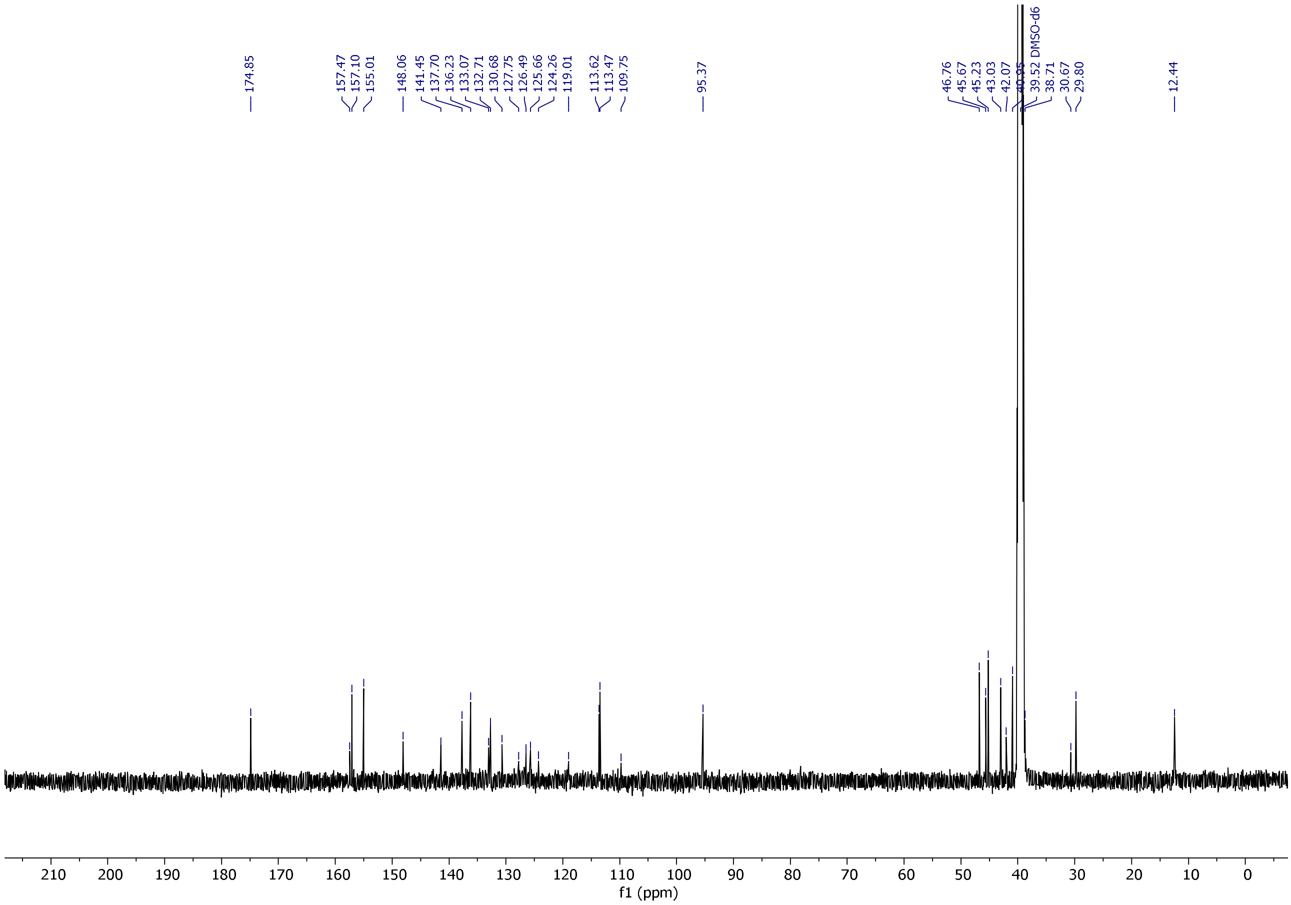

Pro-Dox

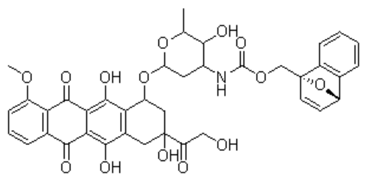

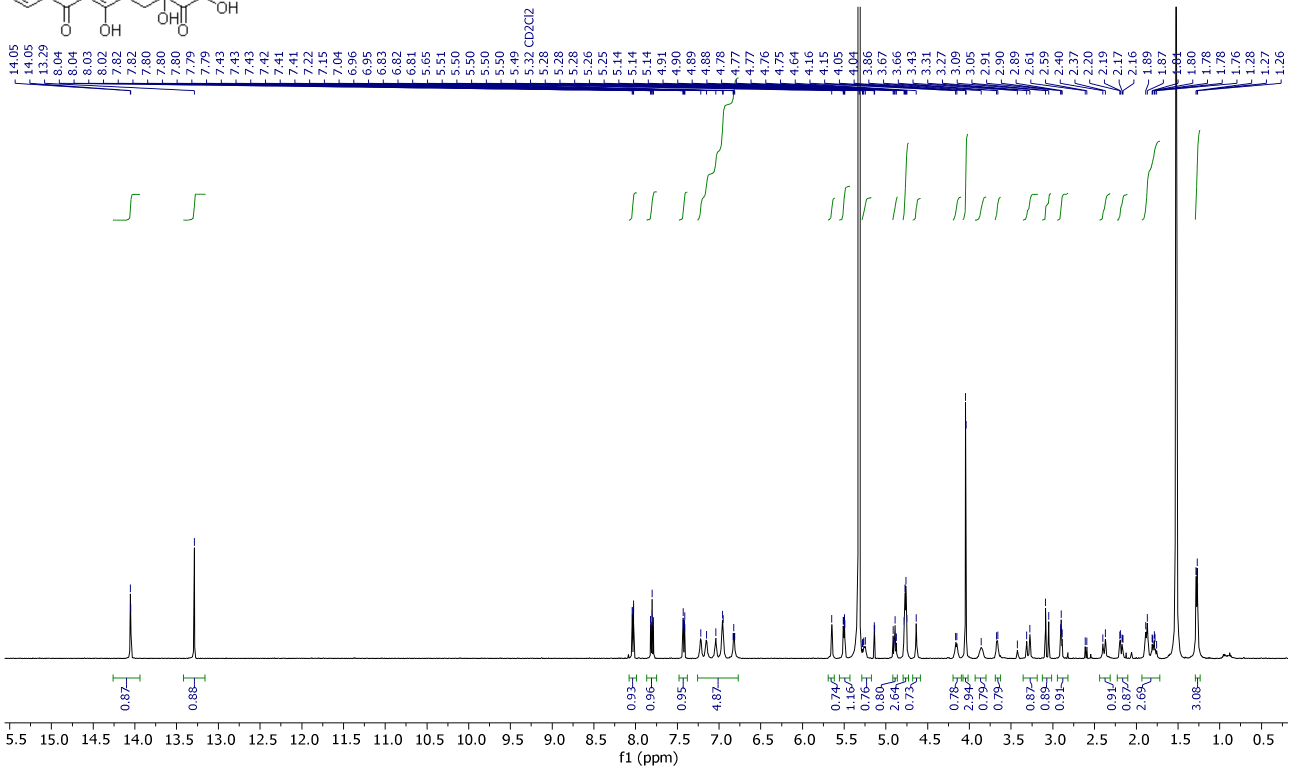

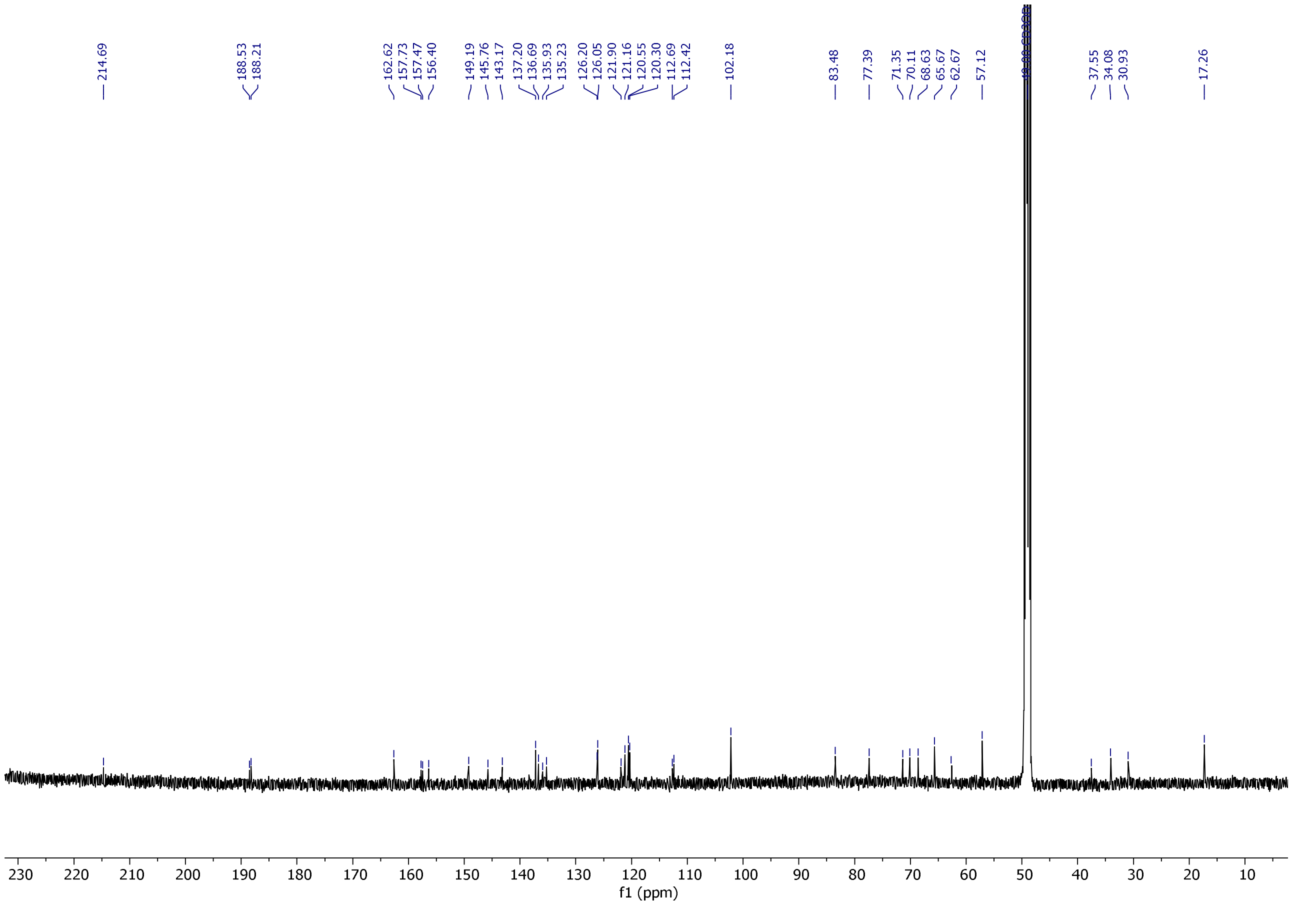

Pro-INK128

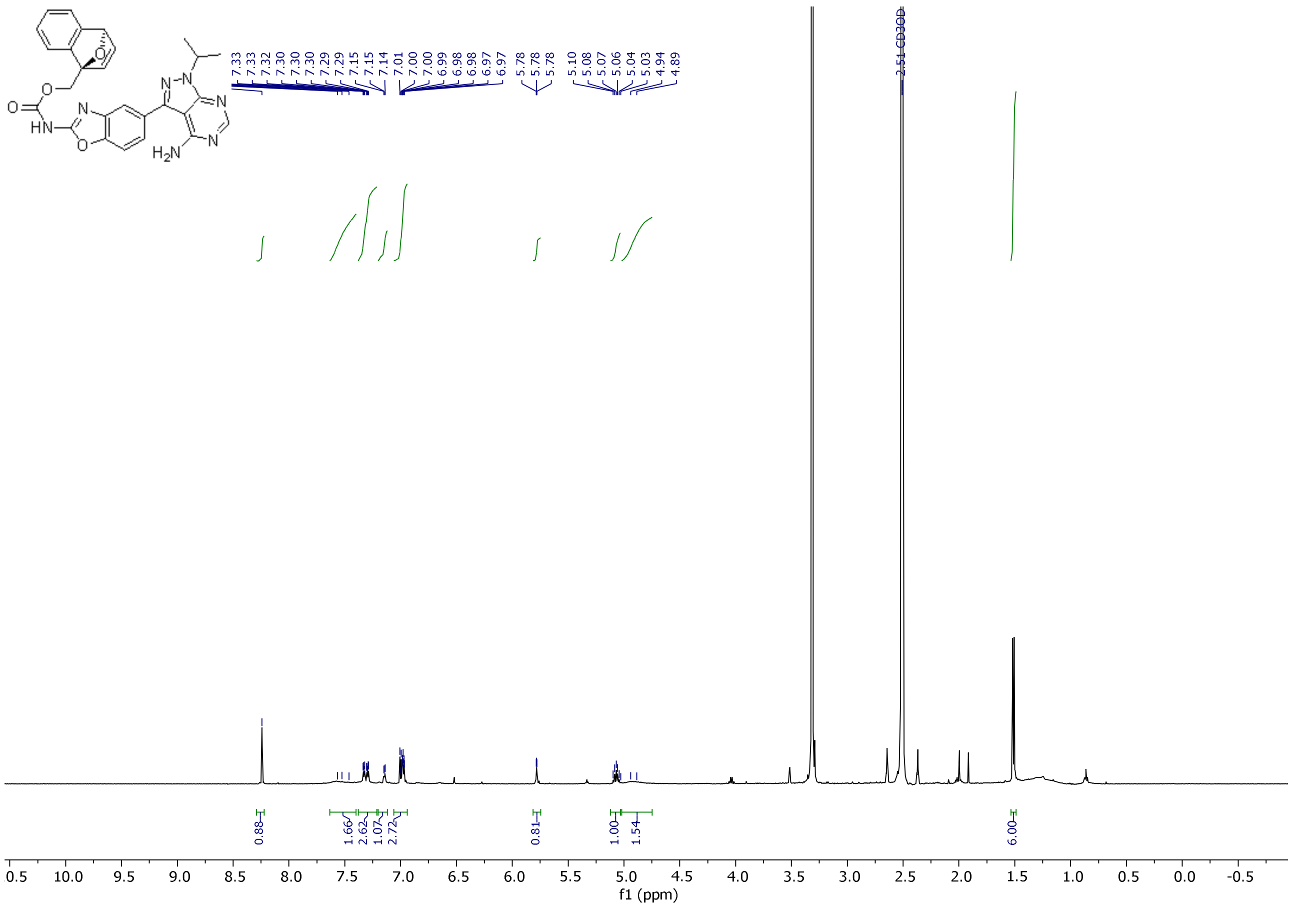
7.33
7.33
7.32
7.30
7.30
7.30
7.29
7.29
7.15
7.15
7.14
7.01
7.00
7.00
6.99
6.98
6.98
6.97
6.97
5.78
5.78
5.78
5.10
5.08
5.07
5.06
5.04
5.03
4.94
4.89
2.51 CD3OD
0.88
1.66
2.62
1.07
2.72
0.81
1.00
1.54
6.00
f1 (ppm)

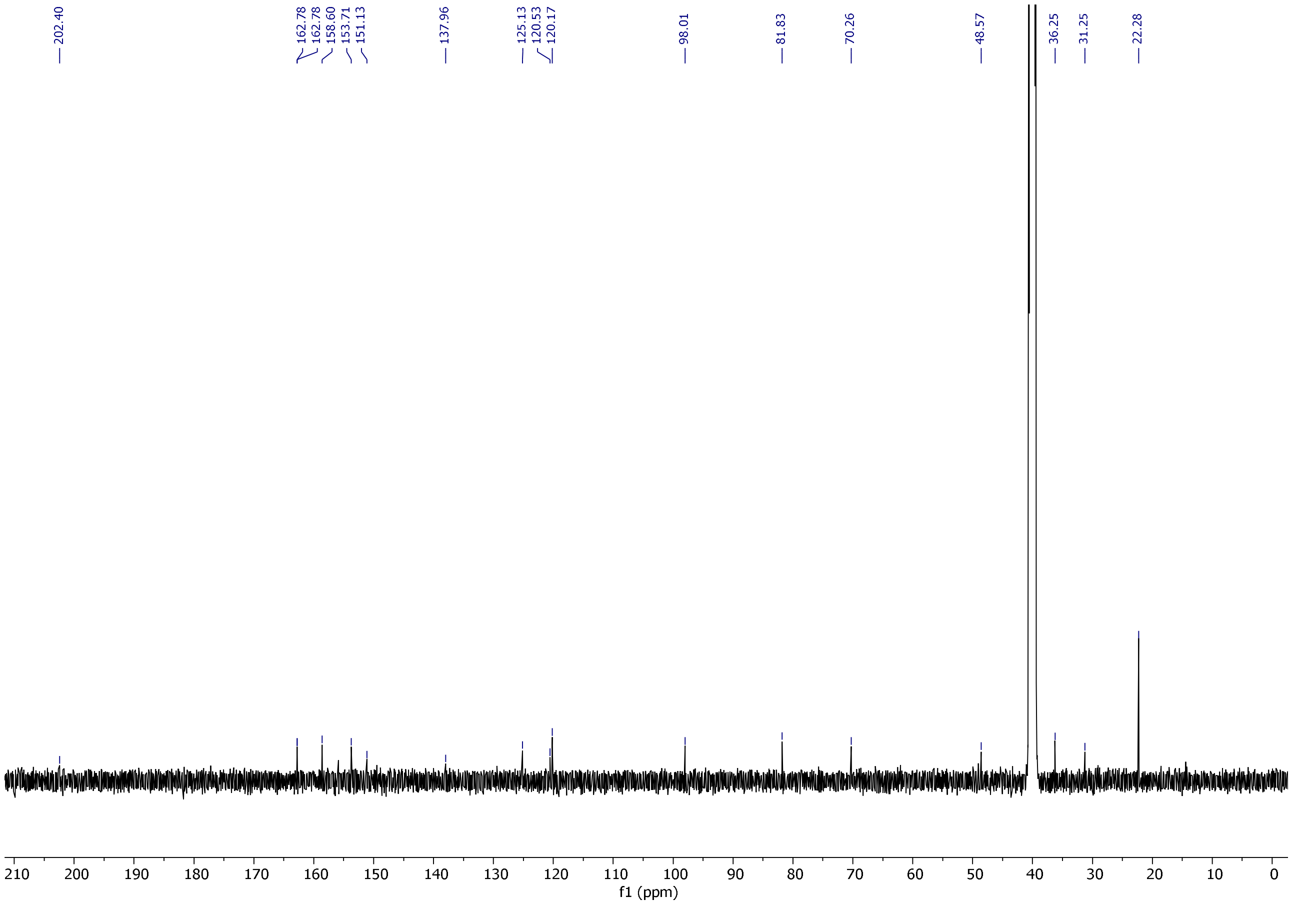
202.40
162.78
162.78
158.60
153.71
151.13
137.96
125.13
120.53
120.17
98.01
81.83
70.26
48.57
36.25
31.25
22.28
f1 (ppm)
